# Abyssal fauna of polymetallic nodule exploration areas, eastern Clarion-Clipperton Zone, central Pacific Ocean: Annelida: Capitellidae, Opheliidae, Scalibregmatidae, and Travisiidae

**DOI:** 10.3897/zookeys.883.36193

**Published:** 2019-10-28

**Authors:** Helena Wiklund, Lenka Neal, Adrian G. Glover, Regan Drennan, Muriel Rabone, Thomas G. Dahlgren

**Affiliations:** 1 Life Sciences Department, Natural History Museum, London SW7 5BD, UK University of Gothenburg Gothenburg Sweden; 2 NORCE Norwegian Research Centre, Bergen, Norway Natural History Museum London United Kingdom; 3 Department of Marine Sciences, University of Gothenburg, Box 463, 40530 Gothenburg, Sweden Gothenburg Global Biodiversity Centre Gothenburg Sweden; 4 Gothenburg Global Biodiversity Centre, Box 463, 40530 Gothenburg, Sweden NORCE Norwegian Research Centre Bergen Norway

**Keywords:** CCZ, deep-sea mining, molecular phylogeny, new species, Polychaeta, Scolecida, 18S, 16S, COI

## Abstract

We present DNA taxonomy of abyssal polychaete worms from the eastern Clarion-Clipperton Zone (CCZ), central Pacific Ocean, using material collected as part of the Abyssal Baseline (ABYSSLINE) environmental survey cruises ‘AB01’ and ‘AB02’ to the UK Seabed Resources Ltd (UKSRL) polymetallic nodule exploration contract area ‘UK-1’, the Ocean Mineral Singapore exploration contract area ‘OMS-1’ and an Area of Particular Environmental Interest, ‘APEI-6’. This is the fourth paper in a series to provide regional taxonomic data with previous papers reporting on Cnidaria, Echinodermata and Mollusca. Taxonomic data are presented for 23 species from 85 records within four polychaete families: Capitellidae, Opheliidae, Scalibregmatidae and Travisiidae, identified by a combination of morphological and genetic data, including molecular phylogenetic analyses. Two taxa (genetically separated from one another) morphologically matched the same known cosmopolitan species, *Ophelina abranchiata* that has a type locality in a different ocean basin and depth from where no genetic data was available. These two species were assigned the open nomenclature ‘cf.’ as a precautionary approach in taxon assignments to avoid over-estimating species ranges. Twelve (12) taxa are here described as new species, *Ammotrypanella keenani***sp. nov.**, *Ammotrypanella kersteni***sp. nov.**, *Ophelina curli***sp. nov.**, *Ophelina ganae***sp. nov.**, *Ophelina juhazi***sp. nov.**, *Ophelina martinezarbizui***sp. nov.**, *Ophelina meyerae***sp. nov.**, *Ophelina nunnallyi***sp. nov.**, *Oligobregma brasierae***sp. nov.**, *Oligobregma tani***sp. nov.**, *Oligobregma whaleyi***sp. nov.** and *Travisia zieglerae***sp. nov.** For the remaining nine taxa, we have determined them to be potentially new species, for which we make the raw data, imagery and vouchers available for future taxonomic study. The CCZ is a region undergoing intense exploration for potential deep-sea mineral extraction from polymetallic nodules. We present these data to facilitate future taxonomic and environmental impact study by making both data and voucher materials available through curated and accessible biological collections.

## Introduction

In the last decades there has been rapid growth in the commercial exploration of the abyssal deep sea for mineral resources (Gollner et al. 2017). One area that has received particular attention is the Clarion-Clipperton Zone (CCZ) in the central Pacific Ocean which is extremely rich in high-grade polymetallic nodules (Baker and Beaudoin 2013; Hein et al. 2013). There is no strict definition of the region, which lies in international waters, but it has come to be regarded as the area between the Clarion and Clipperton Fracture Zones. Exploration licenses issued by the International Seabed Authority (ISA 2017) extend from 115°W (the easternmost extent of the UK-1 claim) to approximately 158°W (the westernmost extent of the COMRA claim). As such we use from hereafter a working definition of the CCZ as the box: 13°N, 158°W; 18°N, 118°W; 10°N, 112°W; 2°N, 155°W. This is an area of almost 6 million km^2^, approximately 1.4% of the ocean’s surface, that is undergoing intense deep-sea mineral exploration for high-grade polymetallic nodules regulated by Sponsoring States (nation states that support a contractor) and the International Seabed Authority.

Annelida is one of the most abundant macrofauna groups on soft bottoms at abyssal depths (e.g. Hessler and Jumars 1974), and in the deep sea the annelid species diversity is generally high even when abundance is low. Quantitative comparisons provide evidence that the central Pacific abyss harbors the highest known deep-sea annelid diversity (Neal et al. 2011), and a recent review of global taxonomic records reported 276 species at depths between 4000 and 5000 m (Paterson et al. 2009). Online database sources prior to 2018 listed only 12 annelid records within or adjacent to the entire 6 million km^2^ of CCZ as defined above (OBIS 2018). Nine of these records are identified to family only as they were observed from remotely operated vehicle (ROV) footage (Amon et al. 2017). Two records have their type locality within the CCZ, *Prionospio branchilucida* Altamira, Glover & Paterson in Paterson et al. 2016 and *Kirkegaardia fragilis* Blake, 2016. The last of these records, collected in 1899 just northwest of CCZ, is *Eunice antillensis* Ehlers, 1887, although the identity of that record is dubious as the type locality for that species is in Gulf of Mexico at 185 m depth (Ehlers 1887).

In terms of recent molecular studies, Janssen et al. (2015) published 556 COI sequences from polychaetes collected in the German and French license areas, but most taxa were identified to family or genus level or in a few cases tentative species names using ‘cf.’ to register similarity but with a precautionary approach. In a paper based on samples collected in the easternmost part of the CCZ, 278 specimens of polynoid polychaetes are reported belonging to ~80 molecular operational taxonomic units (MOTU), including the formal description of 17 new species and four new genera (Bonifácio and Menot 2018). Thus, despite a large number of both mineral exploration and purely scientific expeditions to the CCZ area, not much of the collected macrofauna have been properly identified and entered in publicly accessible museums and biogeographic databases. This is in part due to the fact that most of the species are new to science (e.g. Glover et al. 2002), and the lack of taxonomic guides leads to published species lists that record, for example, only ‘sp. A’, ‘sp. B’, etc., which makes species-level comparisons over a larger area infeasible and presents major issues for the long-term iterative improvement of taxonomic knowledge (Glover et al. 2018).

**Figure 1.**
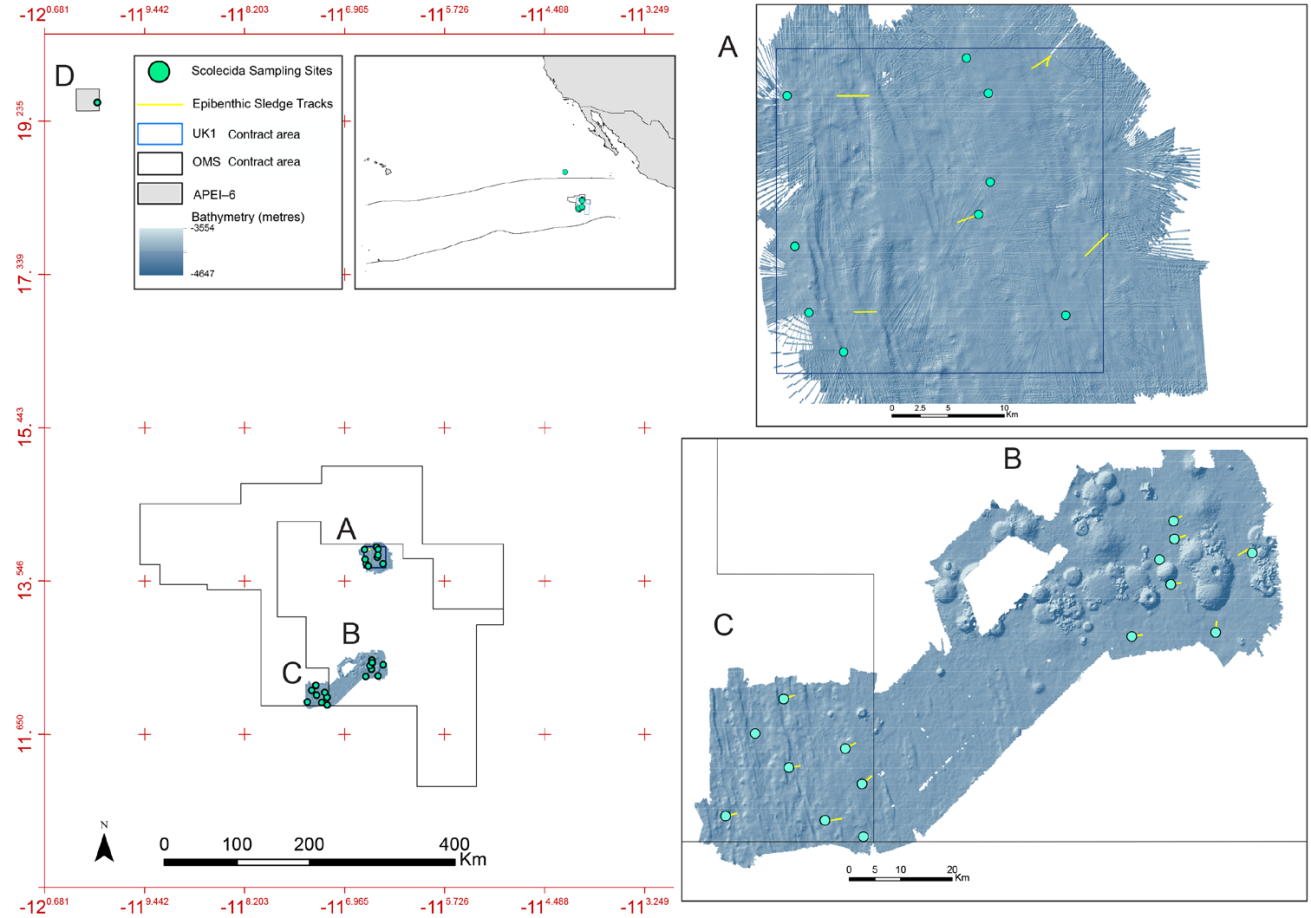
Map over sampling sites. **A** UK-1 Stratum-A **B** UK-1 Stratum-B study areas, both within the UK Seabed Resources UK-1 exploration contract area **C** OMS Stratum-A study area, in the Ocean Mineral Singapore (OMS) polymetallic nodule exploration contract area **D** Area of Particular Interest APEI-6. Inset map showing location of Clarion-Clipperton Fracture Zone. Bathymetric survey and sample localities from the AB01 2013 RV Melville survey cruise and AB02 2015 Thomas G. Thompson survey cruise, data courtesy Craig R. Smith (University of Hawaii), UK Seabed Resources Ltd and Seafloor Investigations, LLC.

The DNA taxonomy part of the UK Seabed Resources (UKSR) program aims to fill some gaps in our knowledge and make data publicly available that will eventually allow for a complete taxonomic synthesis of the CCZ supported by openly available molecular and morphological data. We present results from a DNA taxonomy survey of abyssal benthic annelids collected as part of the UKSR ABYSSLINE cruises ‘AB01’ and ‘AB02’ to the UK Seabed Resources Ltd (UKSRL) polymetallic nodule exploration contract area ‘UK-1’, Ocean Mineral Singapore contract area ‘OMS-1’, and an Area of Particular Environmental Interest, ‘APEI-6’, (Fig. 1) in the eastern CCZ (Smith et al. 2013, 2015). Here we provide the first part of the Annelida taxonomic synthesis consisting of taxon records, images, genetic data and morphological descriptions from the first research cruise (AB01) aboard the RV *Melville* in October 2013 and the second (AB02) aboard the RV *Thomas G. Thompson* in February and March 2015. This part contains the families Capitellidae, Opheliidae, Scalibregmatidae and Travisiidae, and includes 12 new species descriptions. The etymology of the new taxon names is based on a randomised list of members (crew and scientists) of the two research cruises to recognise the team effort involved in this extensive sampling program. This publication is supported by similar data publications on other taxa from the CCZ. The published papers include Echinodermata (Glover et al. 2016b), Cnidaria (Dahlgren et al. 2016), and Mollusca (Wiklund et al. 2017), while other taxa are in preparation, forming a suite of taxonomic syntheses of biodiversity in the region, supported by a contract between the UKSRL, the Natural History Museum, London and NORCE Norwegian Research Centre, Bergen.

## Materials and methods

### Fieldwork

The first UKSR ABYSSLINE cruise (AB01), sampling the UK-1 exploration contract area, took place in October 2013 aboard the RV *Melville*, and the second cruise (AB02), sampling the UK-1 and OMS-1 exploration contract areas and APEI-6, took place in February-March 2015 onboard RV *Thomas G. Thompson*.

A comprehensive description of our DNA taxonomy pipeline is provided in Glover et al. (2016a). In summary, deep-sea benthic specimens from the UK-1, OMS-1 and APEI-6 areas were collected using a range of oceanographic sampling gear including box core, epibenthic sledge (EBS), ROV and multiple core. Geographic data from sampling activities were recorded on a central GIS database (Fig. 1). Live-sorting of specimen samples was carried out aboard both vessels in a ‘cold-chain’ pipeline, in which material was constantly maintained in chilled, filtered seawater held at 2–4 °C. Specimens were preliminarily identified at sea and imaged live using stereo microscopes with attached digital cameras (Glover et al. 2016a). The specimens were then stored in individual microtube vials containing an aqueous solution of 80% non-denatured ethanol, numbered and barcoded into a database and kept chilled until return to the Natural History Museum, London, UK.

### Laboratory work

In the laboratory, specimens were re-examined using stereo and compound microscopes, identified and described to the best possible taxonomic level with key morphological features photographed with digital cameras and a small tissue-sample taken for DNA extraction. Shirlastain A and Methyl Green stain were used during the morphological examination on some specimens, in order to better observe certain characters. Methyl Green stain was limited to Capitellidae, as the staining pattern is considered of value in capitellid taxonomy (e.g. Blake 2009). The other taxa were stained exclusively with Shirlastain A. Scanning electron microscopy (SEM) using a SEM FEI Quanta 650 was conducted on several whole specimens, following a preparation of graded ethanol dehydration, critical point drying, and gold coating. Figures were assembled using Adobe Photoshop CS6 software. A fine white or black line was used to outline and highlight particular morphological features where such features were unclear from images alone.

Extraction of DNA was done with DNeasy Blood and Tissue Kit (Qiagen) using a Hamilton Microlab STAR Robotic Workstation. About 1800 bp of 18S, 450 bp of 16S, and 650 bp of cytochrome c oxidase subunit I (COI) were amplified using primers listed in Table 1. PCR mixtures contained 1 µl of each primer (10 µM), 2 µl template DNA and 21 µl of Red Taq DNA Polymerase 1.1X MasterMix (VWR) in a mixture of total 25 µl. The PCR amplification profile consisted of initial denaturation at 95 °C for 5 min, 35 cycles of denaturation at 94 °C for 45 s, annealing at 55 °C for 45 s, extension at 72 °C for 2 min, and a final extension at 72 °C for 10 min. PCR products were purified using Millipore Multiscreen 96-well PCR Purification System, and sequencing was performed on an ABI 3730XL DNA Analyser (Applied Biosystems) at The Natural History Museum Sequencing Facility, using the same primers as in the PCR reactions plus two internal primers for 18S (Table 1). Overlapping sequence fragments were merged into consensus sequences using Geneious (Kearse et al. 2012) and aligned using MAFFT (Katoh et al. 2002) for 18S and 16S, and MUSCLE (Edgar 2004) for COI, both programs used as plugins in Geneious, with default settings. Bayesian phylogenetic analyses were conducted with MrBayes 3.2.6 (Ronquist et al. 2012). Analyses were run for 10–30 million generations, of which the first 25% generations were discarded as burn-in.

**Table 1.** Primers used for PCR and sequencing of 18S, COI and 16S.

Primer	Sequence 5’-3’	Reference
**18S**		
18SA	AYCTGGTTGATCCTGCCAGT	Medlin et al. 1988
18SB	ACCTTGTTACGACTTTTACTTCCTC	Nygren and Sundberg 2003
620F	TAAAGYTGYTGCAGTTAAA	Nygren and Sundberg 2003
1324R	CGGCCATGCACCACC	Cohen et al. 1998
**COI**		
LCO1490	GGTCAACAAATCATAAAGATATTGG	Folmer et al. 1994
HCO2198	TAAACTTCAGGGTGACCAAAAAATCA	Folmer et al. 1994
COI-E	TATACTTCTGGGTGTCCGAAGAATCA	Bely and Wray 2004
polyLCO	GAYTATWTTCAACAAATCATAAAGATATTGG	Carr et al. 2011
polyHCO	TAMACTTCWGGGTGACCAAARAATCA	Carr et al. 2011
**16S**		
ann16SF	GCGGTATCCTGACCGTRCWAAGGTA	Sjölin et al. 2005
16SbrH	CCGGTCTGAACTCAGATCACGT	Palumbi 1996

### Data handling

The field and laboratory work created a series of databases and sample sets that are integrated into a data-management pipeline. This includes the transfer and management of data and samples between a central collections database, a molecular collections database and external repositories (GenBank, WoRMS, OBIS, GBIF, GGBN, ZooBank) through DarwinCore archives and usage of the GGBN data standard (Droege et al. 2014). This provides a robust data framework to support DNA taxonomy, in which openly available data and voucher material is key to quality data standards. A further elaboration of the data pipeline is published in Glover et al. (2016a).

### Taxonomic assignments

Future studies of biogeographic and bathymetric ranges, gene-flow, extinction risks, natural history, reproductive ecology, functional ecology and geochemical interactions of CCZ species are dependent on accurate taxonomic identifications. This taxonomy is dependent on a sound theoretical underpinning—a species concept—coupled with the availability of both raw data and voucher samples. Here we use a phylogenetic species concept sensu Donoghue (1985) with species determined by DNA-based phylogenetic analysis. For those taxa where the morphological data that allows description of a new species is missing, we provide the lowest-level taxonomic name possible aided by phylogenetic information. In these cases, we use an informal naming system where the best available voucher specimen number is used as the informal species name, for example *Notomastus* sp. (NHM_162) is the informal species name for all specimens that are the same species as the specimen number NHM_162. This avoids confusion with the use of sp. A, B, C, etc. where informal and confusing synonyms can easily arise. Type material, DNA specimen vouchers and DNA extractions are deposited at the Natural History Museum, London. A full list of all taxa including Natural History Museum Accession Numbers (NHMUK), NHM Molecular Collection Facility (NHM-MCF), and NCBI GenBank accession numbers is provided in Table 2.

**Table 2.** Taxon treatments presented in this paper. Includes family, DNA taxonomy ID (a species-level identification based on combined DNA and morphological evidence), GUID (Global Unique Identifier link to data record at http://data.nhm.ac.uk), ABYSSLINE record number, NHM accession number, NHM Molecular Collection Facility (MCF) sample ID number (NHMUK_MCF#) and NCBI GenBank accession number (Genbank#) for successfully sequenced genetic markers. GenBank numbers for phylogenetic analysis data downloaded from GenBank are presented in Supplementary material 1.

Family	DNA taxonomy ID	ABYSSLINE record	GUID	NHMUK Record no.	NHMUK MCF no.	Genbank no.
Capitellidae	Capitellidae sp. (NHM_1486)	NHM_613	be34eb86-fc0c-411c-8eb9-e86774c6515a	2019.7105	0109494702	MN217415
						MN217495
		NHM_1486	05d46c60-8b4d-4623-b7ff-2e8089453d7e	2019.7106	0109494649	MN217416
						MN217496
	*Notomastus* sp. (NHM_162)	NHM_162	f34e2921-7b6d-4c14-b217-690d9315f073	2019.7100	0109494624	MN217417
						MN217497
		NHM_915B	98291a2a-c89a-4f62-bde5-91171368c749	2019.7101	0109494712	MN217418
		NHM_1025D	3e34e783-a51b-4a84-bc2e-6a19bac82b4e	2019.7102	0109494679	MN217419
		NHM_1200	2612dd53-cce5-47b9-aeed-c321bda6c3d8	2019.7103	0109494687	MN217420
		NHM_1948J	24374a21-17b6-4de5-8436-18c160aa5c8d	2019.7104	0109494636	MN217421
Opheliidae	*Ammotrypanella keenani* sp. nov.	NHM_1166C	483c6faa-0338-4cf5-a21d-6f448c72f4aa	2019.7109	0109494704	MN217408
						MN217513
		NHM_1250 (holotype)	1514d25d-b485-4b90-8981-3e84381bf250	2019.7110	0109494644	MN217409
						MN217491
						MN217514
		NHM_1871 (paratype)	d93680b5-a3a3-4623-afc4-17062e1a1a58	2019.7111	0109494707	MN217410
		NHM_1949	cff2696d-06ab-42a1-843e-6ef894872f32	2019.7112	0109494683	MN217411
	*Ammotrypanella kersteni* sp. nov.	NHM_254 (holotype)	3441cd68-7432-4dee-8415-966b104c3077	2019.7107	0109494672	MN217412
						MN217492
						MN217515
	*Ammotrypanella* sp. (NHM_1653)	NHM_1653	a2f7ed04-7275-4a57-a058-bd750cacc715	2019.7108	0109494685	MN217413
						MN217493
						MN217516
	*Ammotrypanella* sp. (NHM_2114)	NHM_2114	f4492dd1-8088-47c6-97d9-32e43ae99552	2019.7113	0109494699	MN217414
						MN217494
	*Ophelina curli* sp. nov.	NHM_2112 (holotype)	c1554f01-2324-4d8d-b775-dca42f5918e7	2019.7131	0109494716	MN217435
						MN217502
	*Ophelina ganae* sp. nov.	NHM_245	3a34b9cb-504b-48a3-a8e9-93077ec69520	2019.7140	0109494696	MN217436
						MN217503
						MN217521
		NHM_248	e67f7724-8c9f-4463-943e-7cda20441728	2019.7141	0109494197	MN217437
		NHM_473	79dcab18-936b-430e-b770-6aab60d285c5	2019.7142	0109494671	MN217438
						MN217522
		NHM_598 (paratype)	2fa20a59-8bb3-4ef8-b2e9-efccbe2c9414	2019.7143	0109494713	MN217439
		NHM_708	2077c6c6-0e3e-4dfa-97a0-16d6c386ff07	2019.7144	0109494665	MN217440
		NHM_1098	5661fb64-83a2-4e9a-b3c3-a8405705ed1a	2019.7145	0109494631	MN217441
		NHM_1137 (holotype)	11616c16-bdb5-4813-9d17-7170bb62702b	2019.7146	0109494711	MN217442
		NHM_1309 (paratype)	1ff41b52-9801-4b2e-8e01-ea34597b708d	2019.7147	0109494639	MN217523
Opheliidae	*Ophelina juhazi* sp. nov.	NHM_1073 (holotype)	f7330230-224b-49e7-aa80-41e8654ea087	2019.7132	0109494655	MN217443
						MN217504
	*Ophelina martinezarbizui* sp. nov.	NHM_681 (holotype)	0de17415-a8bf-4461-a663-dea9a3e6a2b9	2019.7116	0109494717	MN217444
						MN217505
						MN217524
		NHM_718	f6e2fa9b-a479-4e0d-aec6-57efff6987b2	2019.7117	0109494693	MN217445
						MN217525
		NHM_883	d9a3a3b3-c16e-4359-8eb0-f09deed98401	2019.7118	0109494627	MN217446
						MN217526
		NHM_994	4f6d2b7a-169f-46a9-8b3b-5d91a021aa34	2019.7119	0109494626	MN217447
		NHM_1066	972f9cb1-79d7-4296-a6d6-e04543c9105c	2019.7120	0109494664	MN217448
		NHM_1766 (paratype)	dc754b1c-e66b-4a58-a93e-796ebfd32f6a	2019.7121	0109494658	MN217449
						MN217527
		NHM_1870	b6247e8d-d155-4646-87d7-e5358ada5352	2019.7122	0109494708	MN217450
		NHM_2088	7dd04f2c-435b-44b1-a85f-3b05dd3014d7	2019.7123	0109494669	MN217451
		NHM_2092	1a095836-fa97-48b8-ad4c-07ed28356ecb	2019.7124	0109494650	MN217528
		NHM_2102	93e91313-61a3-4cd7-8221-66bf20232f14	2019.7125	0109494645	MN217452
						MN217529
		NHM_2116 (paratype)	d439156e-657d-4dd5-8bb5-3531e150961e	2019.7126	0109494692	MN217453
						MN217530
		NHM_2144	79767cab-eb56-4ef1-acd0-5067ec3736de	2019.7127	0109494668	MN217454
		NHM_2149	1caa9eb3-3281-4ed6-8424-dfaebcf1e20b	2019.7128	0109494675	MN217455
		NHM_2150	993f577c-ee86-4660-b2d9-af0146606f92	2019.7129	0109494651	MN217456
						MN217531
	*Ophelina meyerae* sp. nov.	NHM_1241 (holotype)	920d8670-507e-4126-a42b-6e208bbe66d3	2019.7130	0109494220	MN217457
						MN217506
	*Ophelina nunnallyi* sp. nov.	NHM_683 (holotype)	220fa671-4576-45b7-930d-efde148f223f	2019.7133	0109494235	MN217458
						MN217507
						MN217532
		NHM_700 (paratype)	63115f48-bcf1-4b3b-9c2e-c339b97845bd	2019.7134	0109494678	MN217459
		NHM_783F	376a42db-0497-4b4a-851b-c1d5e07bd2b6	2019.7135	0109494630	MN217460
		NHM_1273 (paratype)	a3540563-8a0c-475b-96b5-12969fb8c2ba	2019.7136	0109494663	MN217461 MN217533
		NHM_1309A	25066e63-ecc9-439a-9907-eaeaeb72e78c	2019.7137	0109494656	MN217462
						MN217534
	Ophelina cf. abranchiata sp. (NHM_1769)	NHM_1769	8a2cbe4f-277d-4355-a34f-0b53c797bef0	2019.7148	0109494637	MN217433
						MN217501
						MN217520
	Ophelina cf. abranchiata sp. (NHM_2017)	NHM_2017	9ebcd947-c53b-4616-81d4-da42afaeca03	2019.7149	0109494660	MN217434
	*Ophelina* sp. (NHM_689)	NHM_689	6755d584-a20a-4ce5-a4f1-32ce0965128e	2019.7114	0109494689	MN217463
						MN217508
	*Ophelina* sp. (NHM_1068)	NHM_1068	b28fd52f-5717-45e3-b0cc-369172a690e5	2019.7138	0109494646	MN217464
						MN217509
		NHM_1874	c3ffe5f4-6ca3-4816-966c-25ec98bbb003	2019.7139	0109494684	MN217466
	*Ophelina* sp. (NHM_1331)	NHM_1331	06d48d7f-7339-4cc5-8445-b51a980e4e0f	2019.7115	0109494710	MN217465
						MN217510
Scalibregmatidae	*Oligobregma brasierae* sp. nov.	NHM_032	43545746-b8ad-43a8-92b7-53637dd131d6	2019.7150	0109494647	MN217422
		NHM_404	5fda0cac-0a77-4ec7-a2fa-5cd529548a19	2019.7151	0109494694	MN217423
		NHM_684 (paratype)	d84c37ed-138e-4064-a11d-a11a2470dfdf	2019.7152	0109494698	MN217424
						MN217498
						MN217517
		NHM_823 (holotype)	74781dbb-1f65-4839-a766-24d6cde63ed0	2019.7153	0109494676	MN217425
		NHM_1423 (paratype)	d949e987-6e03-4092-8492-c51dd7fcf4d7	2019.7154	0109494681	MN217426
		NHM_1895	02aaa9c0-837a-4836-8b34-5e68296c958e	2019.7155	0109494643	MN217427
	*Oligobregma tani* sp. nov.	NHM_773A (paratype)	4b673a6a-9090-4c24-a4eb-231190507b60	2019.7156	0109494629	MN217428
						MN217499
						MN217518
		NHM_1454 (holotype)	67d3f58a-9c13-423e-93b7-3ddcf98a361e	2019.7157	0109494662	MN217429
		NHM_1480J	d47f17aa-c0c1-44f0-a448-d3f3c395fc47	2019.7158	0109494633	MN217430
		NHM_1665 (paratype)	eca166ae-3fe0-4367-860f-08c7410165dd	2019.7159	0109494661	MN217431
						MN217519
	*Oligobregma whaleyi* sp. nov.	NHM_822 (holotype)	dde1c8f9-f87a-430b-be9d-5e34685772bb	2019.7160	0109494667	MN217432
						MN217500
	Scalibregmatidae sp. (NHM_2308)	NHM_2308	7b9d4ab8-4b7b-45c4-9cf4-6fd6b1229f48	2019.7161	0109494623	MN217467
Travisiidae	*Travisia zieglerae* sp. nov.	NHM_140 (paratype)	ed10356b-32a0-4b45-9fe3-c56fbc696e87	2019.7162	0109494719	MN217470
						MN217512
		NHM_188	c8a0ef70-e7f7-4605-bf78-dc54ed9151eb	2019.7170	0109494718	MN217471
		NHM_241	5c0ac0b7-60cc-473e-a23b-2f49a40540f4	2019.7163	0109494648	MN217472
		NHM_356	8d2cbf0e-6522-403d-a58a-905fb13c70d6	2019.7164	0109494697	MN217473
		NHM_364	ef6e520f-7ef5-4ff9-87b5-985b8576271f	2019.7165	0109494673	MN217474
		NHM_748B (paratype)	db527676-1030-4bf0-b28d-2382825bc6bf	2019.7166	0109494654	MN217475
		NHM_753	393203b1-cb80-4185-9e40-fca6e1b6fe34	2019.7167	0109494715	MN217476
		NHM_760	d3e8ec3c-d7f3-4908-b315-84f3758aecc1	2019.7168	0109494691	MN217477
		NHM_792	5d30a61b-5894-484f-b79a-df1cd4268ec1	2019.7169	0109494641	MN217478
		NHM_909 (paratype)	5f570dab-4b56-4f74-b126-ed6ceab344e3	2019.7171	0109494670	MN217479
		NHM_970	4ccb364c-35f4-458c-9c71-6f77e71493ca	2019.7172	0109494703	MN217480
		NHM_1097	939ba16d-b844-49ca-a740-bb42f039cc11	2019.7173	0109494625	MN217481
		NHM_1310	16844478-de27-448c-9acb-057835026447	2019.7174	0109494690	MN217482
		NHM_1311	192cbbb3-680b-4bcd-9cc4-a420f42af578	2019.7175	0109494700	MN217483
		NHM_1431 (holotype)	fd6bab0e-0cda-4b42-808f-a6006d409535	2019.7176	0109494211	MN217484
		NHM_1543 (paratype)	c78cc5fd-ca98-43b0-a0fb-8804fb606c71	2019.7177	0109494628	MN217485
Travisiidae	*Travisia zieglerae* sp. nov.	NHM_1873	24409a12-2a50-4689-80dc-902cdeb5af69	2019.7178	0109494642	MN217486
		NHM_1883	9e8c22f7-a94b-45ed-a1d0-cae287a7ac2d	2019.7179	0109494666	MN217487
		NHM_1911	489dd5a6-2c68-416b-9a06-ed773d4791d6	2019.7180	0109494632	MN217488
		NHM_2019	2684a5f8-b4d4-4bcb-b386-65775506cf87	2019.7181	0109494659	MN217489
		NHM_2024	cf54f81e-5836-4684-94dc-151f589ebab4	2019.7182	0109494653	MN217490
	*Travisia* sp. (NHM_1244)	NHM_1244	f6906eae-67ec-4d37-83c6-590f3c53df76	2019.7183	0109494714	MN217468
		NHM_1863	fa708aca-6dd1-4b53-8d54-c76a93f43363	2019.7184	0109494652	MN217469
						MN217511

## Systematics

### Annelida

#### Capitellidae

Taxon classificationAnimaliaCapitellidaCapitellidae

Grube, 1862

DC9E7D71-1173-52D8-B381-1EE7FF102B32

##### Notes.

Capitellidae represent an important group of polychaetes owing to their use as indicators of environmental health (e.g., Tomassetti and Porrello 2005). Despite their importance, capitellids have a confused and unresolved taxonomy, with a large number of often monotypic genera and the presence of species complexes. Capitellid genera are distinguished largely on chaetal distribution in anterior segments and the number of thoracic segments (see e.g., Blake 2000a; Fauchald 1972). The need for revision of the family has been deemed necessary by several authors (e.g., Fauchald 1977; Ewing 1991; Blake 2000a), as it has been observed that chaetal distribution, particularly in the posterior thorax, may change with age (e.g., Ewing 1982; 1984; Blake 2000a). Green (2002) provided a useful overview of characters used in capitellid taxonomy.

At least two species were recognised in the UKSR material, five poorly preserved representatives of a species in the diverse genus *Notomastus*, and two specimens of a species representing a new genus based on morphological and genetic data. However, given the caveats of generic definitions given above, we choose not to provide the formal description of this species and genus, and make these data and vouchers available for future revision.

#### Capitellidae

Taxon classificationAnimaliaCapitellidaCapitellidae

sp. (NHM_1486)

CE792592-061A-5F2B-A4A0-6F798C76CC2D

Fig. 2A–G

##### Material examined.

NHM_613 NHMUK ANEA 2019.7105, coll. 17 Feb. 2015, 12°23.175N, 116°32.92W, 4202 m http://data.nhm.ac.uk/object/be34eb86-fc0c-411c-8eb9-e86774c6515a; NHM_1486 NHMUK ANEA 2019.7106, coll. 04 Mar. 2015, 12°29.70N, 116°39.01W, 4260 m http://data.nhm.ac.uk/object/05d46c60-8b4d-4623-b7ff-2e8089453d7e.

##### Description.

**Figure 2.**
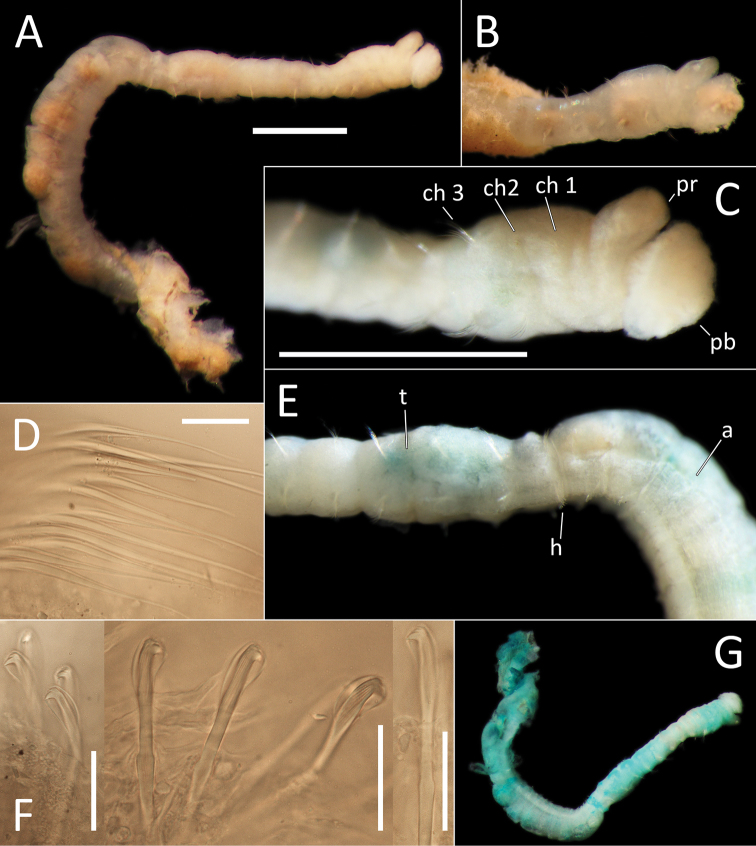
Capitellidae sp. NHM_1486 (specimen NHM_1486). **A** Lab image, whole specimen **B** Live image, anterior **C** Lab image, anterior, highlighting first three chaetigers (faded stain, pr = prostomium, pb = proboscis) **D** Lab image, thoracic capillary chaetae **E** Lab image, transitionary chaetigers between thorax and abdomen (faded stain) (t = thorax, h = hooks, a = abdomen) **F** Lab image, hooks **G** Lab image, whole specimen (methyl green stain). Scale bars: 1 mm (**A, C**); 25 μm (**D, F**).

Species represented by one anterior fragment and one body fragment only. Specimen NHM_1486 posteriorly incomplete, 8 mm long and 0.6 mm wide for about 22 chaetigers (posterior part of fragment is damaged). Preserved specimens creamy white in ethanol (Fig. 2A); live specimens creamy yellow to white semi-translucent (Fig. 2B). Epithelium smooth (Fig. 2).

Prostomium conical, anteriorly broadly rounded, slightly longer than wide (Fig. 2C). Eyespots not observed. Nuchal organs everted, lightly pigmented, located at posterior border of prostomium. Peristomium as a narrow, smooth, achateous ring.

Chaetigers 1–10 (= thorax) with capillaries only. First chaetiger with chaetae in notopodia only, subsequent nine chaetigers with chaetae in both noto- and neuropodia. All thoracic chaetae slender, bilimbate capillaries (Fig. 2D), arranged in two rows, with alternating longer and shorter capillaries, about 10 chaetae per ramus. Genital pores or lateral organs not observed under light microscopy.

Chaetigers 11–12 are considered transitional between thorax and abdomen marked by appearance of hooded hooks only in neuropodia, but segments are of similar thickness to those in anterior part of body (Fig. 2E).

Abdominal segments enlarged (inflated), without lobe (Fig. 2A). All abdominal chaetigers with capillary chaetae only in notopodia and hooded hooks only in neuropodia. Abdominal capillaries similar to those in thorax, about 15 per ramus. Hooded hooks with long and slender shaft, with swelling around mid-point of shaft; with a main fang and about three rows of small teeth (Fig. 2F); about 15 hooks per ramus. Remainder of body unknown.

##### Methyl green stain.

Prostomium, chaetigers 4–6 and abdominal chaetigers do not stain (or at best stain very lightly). Peristomium, chaetigers 1–3 and 7–12/13 stain more strongly (Fig. 2G).

##### Genetic data.

GenBank MN217415-MN217416 for 16S and MN217495-MN217496 for 18S. COI was unsuccessful for this species, no identical matches on GenBank for 16S or 18S. The species is distinct from all other specimens in this study and in our phylogenetic analyses forms an unresolved clade with *Barantolla lepte* Hutchings, 1974, three *Notomastus* M. Sars, 1851 and one *Heteromastus* Eisig, 1887 species (Fig. 4).

##### Remarks.

This species is unusual amongst Capitellidae due to its large number of mixed segments (with capillaries only in notopodia and hooded hooks only in neuropodia). Usually, the abdominal chaetigers in Capitellidae bear hooded hooks only, or there are a small number of posterior thoracic and/or anterior abdominal segments that bear both capillaries and hooks. Of the known genera, only *Promastobranchus* Gallardo, 1968 shows such chaetal distribution. However, it can be distinguished from the other UKSR-collected species in having the first chaetiger with both noto- and neurochaetae and possessing 12 to 13 instead of 10 chaetigers with capillary chaetae in both rami. Therefore, this material represents not only a new species, but based on current morphological criteria supported also by genetic data, a new genus as well. Because the material is not complete (morphology for the posterior end is missing) the species is here not formally described.

##### Ecology.

Found in the eastern polymetallic nodule province of the CCZ.

### *Notomastus* M. Sars, 1851

#### Notomastus

Taxon classificationAnimaliaCapitellidaCapitellidae

sp. (NHM_162)

A79A9A5B-DFCC-53D5-B2FD-99068223CE07

Fig. 3A–I

##### Material examined.

NHM_162 NHMUK ANEA 2019.7100, coll. 13 Oct. 2013, 13°57.794N, 116°34.093W, 4084 m http://data.nhm.ac.uk/object/f34e2921-7b6d-4c14-b217-690d9315f073; NHM_915B NHMUK ANEA 2019.7101, coll. 23 Feb. 2015, 12°34.28N, 116°36.63W, 4198 m http://data.nhm.ac.uk/object/98291a2a-c89a-4f62-bde5-91171368c749; NHM_1025D NHMUK ANEA 2019.7102, coll. 24 Feb. 2015, 12°08.02N, 117°17.52W, 4122 m http://data.nhm.ac.uk/object/3e34e783-a51b-4a84-bc2e-6a19bac82b4e; NHM_1200 NHMUK ANEA 2019.7103 coll. 27 Feb. 2015, 12°00.567N, 117°10.687W, 4144 m http://data.nhm.ac.uk/object/2612dd53-cce5-47b9-aeed-c321bda6c3d8; NHM_1948J NHMUK ANEA 2019.7104, coll. 13 Mar. 2015, 12°02.49N, 117°13.03W, 4094 m http://data.nhm.ac.uk/object/24374a21-17b6-4de5-8436-18c160aa5c8d.

##### Description.

**Figure 3.**
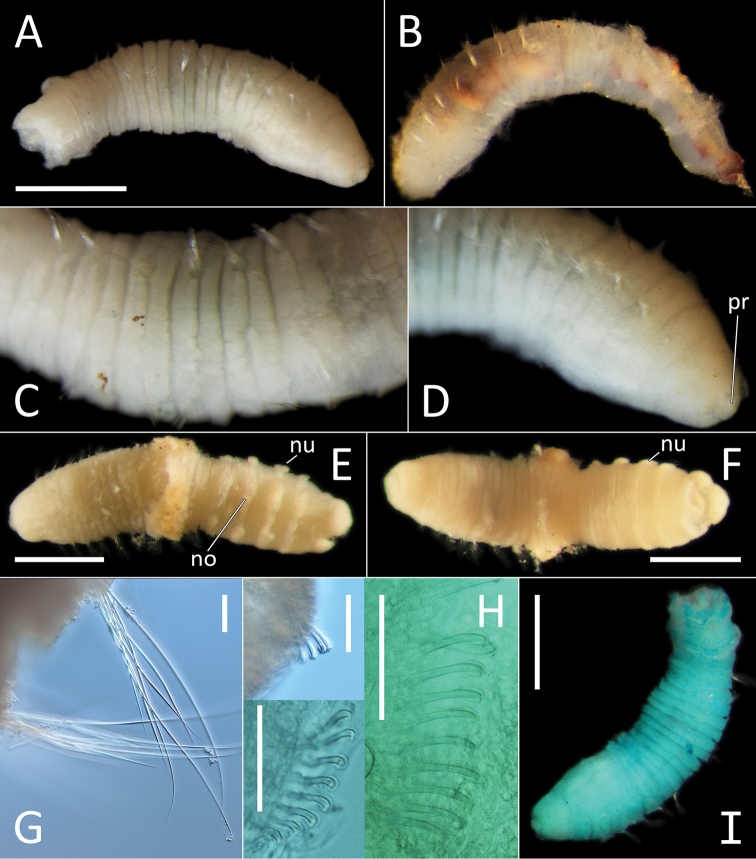
*Notomastus* sp. NHM_162. **A** Lab image, whole specimen (specimen NHM_162) **B** Live image, whole specimen (specimen NHM_162) **C** Lab image, biannulated chaetigers (specimen NHM_162) **D** Lab image, prostomium (specimen NHM_162, pr = prostomium) **E** Lab image, whole specimen, dorsal (specimen NHM_915B, no = notopodial lobe, nu = neuropodial lobe) **F** Lab image, whole specimen, ventral (specimen NHM_915B, nu = neuropodial lobe) **G** Lab image, thoracic chaetae, (specimen NHM_162) **H** Lab image, thoracic hooks (specimen NHM_162) **I** Lab image, whole specimen (specimen NHM_162, methyl green stain). Scale bars: 1 mm (**A, E, F, I**); 50 μm (**G, H**).

All specimens short anterior fragments, posteriorly incomplete with thorax only or thorax and 2–5 abdominal segments only. Small species, 2–4 mm long and 0.3–0.8 mm wide for 11–16 chaetigers. Preserved specimens creamy white in ethanol; live specimens white to light brown semi-opaque/translucent. Epithelium of peristomium and first two chaetigers smooth or at best weakly annulated, on chaetigers 3–11 epithelium tessellated, distinctly bi-annulated (Fig. 3A–D).

Prostomium low, rounded mound (Fig. 3D). Eyespots not observed. Nuchal organs inconspicuous. Peristomium as a broad, non-tessellated, achateous ring.

Thorax with 11 chaetigers, first with notochaetae only. All thoracic chaetae long, slender, bilimbate capillaries (Fig. 3G). Genital pores and lateral organs not observed under light microscopy. Transition between thorax and abdomen marked abruptly by the segment size, neuropodia development and chaetal type.

Abdominal segments with hooded hooks only in both rami. Noto- and neuropodia free laterally, notopodia widely separated dorsally. Abdominal notopodia coalesce into lobe, which protrudes from dorsum (Fig. 3E). Superior edges of the neuropodia taper to broadly rounded lobes (Fig. 3F, G). All abdominal chaetae hooded hooks, about 45 per ramus (Fig. 3H). All hooded hooks similar in shape on noto- and neuropodia; hooks with long shaft, with swelling at mid-point; with a main fang and rows of small teeth. The rest of the body unknown.

##### Methyl green stain.

Anterior fragment with 13 chaetigers staining more or less uniformly; more pronounced in chaetigers 6–11 (Fig. 3I).

##### Genetic data.

**Figure 4.**
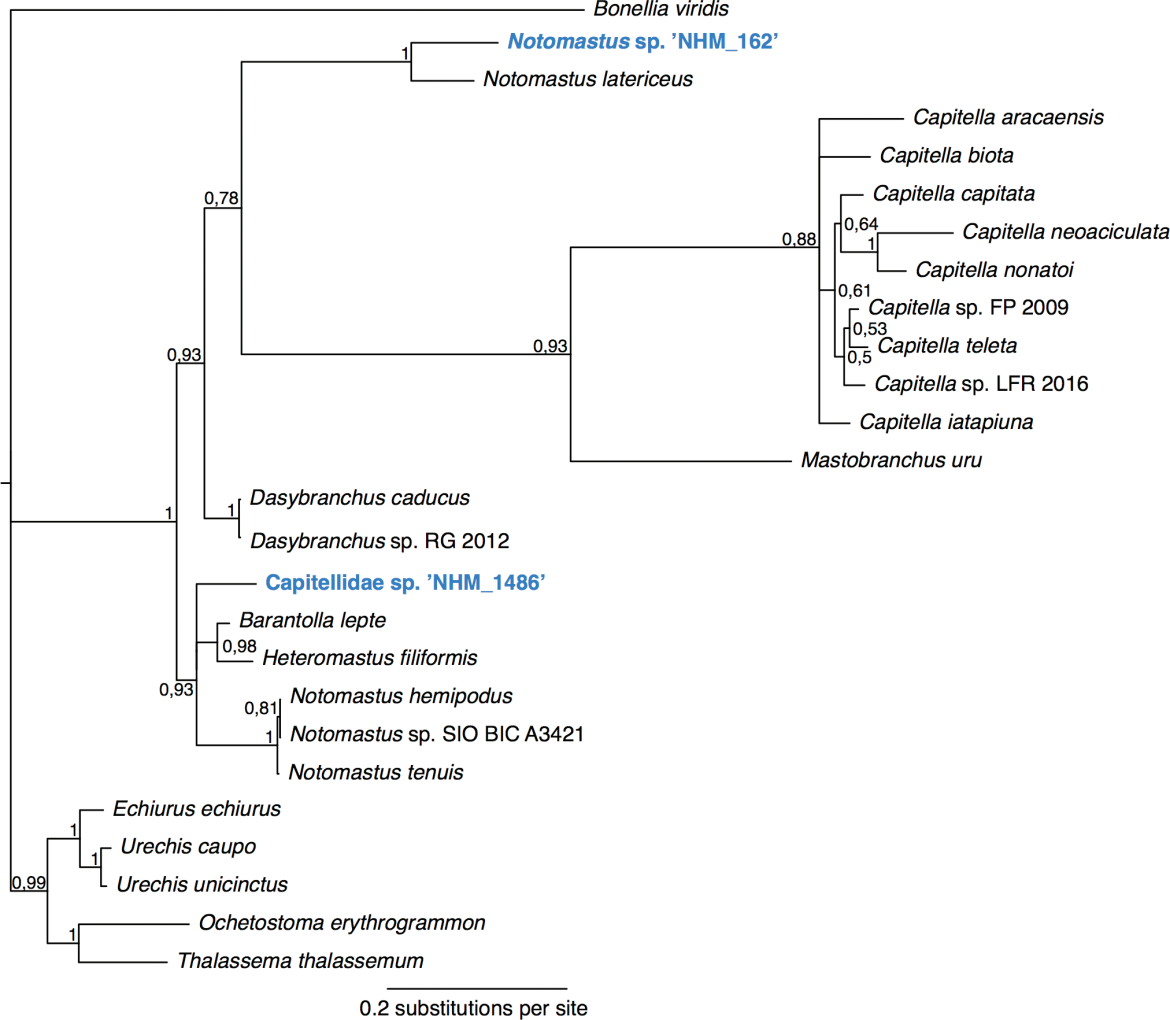
Phylogenetic analysis of Capitellidae. 50% majority rule tree from the Bayesian analyses using 18S and 16S, with posterior probability values on nodes. Twenty-four taxa from GenBank were included, and Echiura was chosen as outgroup following the annelid phylogeny of Weigert and Bleidorn (2016).

GenBank MN217417-MN217421 for 16S, MN217497 for 18S. COI was unsuccessful for this species, no identical matches on GenBank for 16S or 18S. In our phylogenetic tree *Notomastus* sp. (NHM_162) is sister to *Notomastus latericeus* M. Sars, 1851, but the genus *Notomastus* is not monophyletic in our tree (Fig. 4).

##### Remarks.

This species is consistent with the genus *Notomastus* in possessing 11 chaetigers with notochaetae only, followed by abdominal chaetigers with hooded hooks only. As the specimens are poorly preserved with the thorax only or with 2–5 abdominal segments observed, this species cannot be meaningfully compared with other known species in this genus and is therefore not formally described.

##### Ecology.

Found in the eastern polymetallic nodule province of the CCZ.

#### Opheliidae

Taxon classificationAnimaliaCapitellidaCapitellidae

Malmgren, 1867

66B3EF51-6262-56B2-81DC-BED06BD9E7CC

##### Notes.

Due to their simple morphology, there is much confusion in the taxonomic literature dealing with Opheliidae, and many species and genera are currently considered invalid (Read and Fauchald 2018c). Useful recent studies clarifying some of the confusion are Kongsrud et al. (2011) and Parapar et al. (2011) based on material collected from North-East Atlantic. It would appear that several previous descriptions were based on what are in fact different, if morphologically very similar, species. Characters such as the shape of the prostomium and associated palpode are often reported in descriptions and used to distinguish species, but the UKSR material showed that the shape in preserved specimens may be variable and the shape and size of the associated palpode can also vary with preservation. Additionally, the shape of the prostomium in live specimens can be of different shape to that observed in preserved specimens (L. Neal pers. obs.) and thus this character might not be useful. Mouth structures in opheliids are rarely observed and reported but considered important by some authors (Tzetlin and Zhadan 2009). The number of chaetigers in a specimen has been deemed as a useful character by e.g. Kongsrud et al. (2011) and Blake (2000b). The chaetae of the opheliids are relatively uniform smooth capillaries of limited taxonomical importance. However, Sarda et al. (2009) reported the presence of hirsute capillaries in *Ophelina margaleffi* Sarda et al., 2009 observed under SEM. Opheliid branchiae are very fragile structures which are easily lost and thus their distribution and number can be difficult to observe. Kongsrud et al. (2011) illustrated the importance of examining a relatively large number of specimens in order to correctly establish the characters of these structures. The anal tube is also a very fragile structure that is easily lost, and true absence is difficult to distinguish from loss due to damage. Kongsrud et al. (2011) again illustrated the importance of examining a relatively large number of specimens in order to correctly establish the presence and form of this structure.

Unfortunately, the ABYSSLINE material provides very few specimens (often just one) per species, which complicates the morphological interpretation. Nevertheless, in combination with DNA data, we believe it is important to provide the currently best possible morphology, which can be amended as more and better-preserved examples become available in the future. As a result, only 8 out of 15 opheliid species found in the ABYSSLINE material are here formally described. Morphologically, the ABYSSLINE material can be assigned to two known genera, *Ammotrypanella* McIntosh, 1878 and *Ophelina* Örsted, 1843.

#### Ammotrypanella

Taxon classificationAnimaliaCapitellidaCapitellidae

McIntosh, 1878

21665342-F47D-581C-AD5A-0CF152B0C865

Fig. 5A–C

##### Notes.

**Figure 5.**
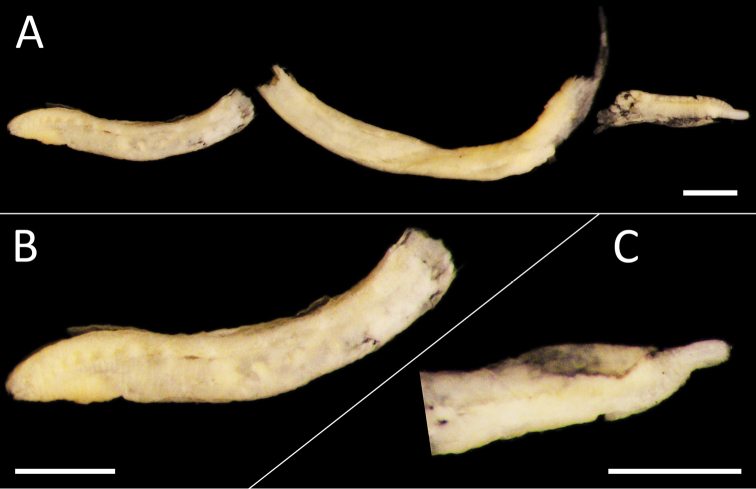
*Ammotrypanella arctica* holotype (BMNH 1921.12392). **A** Whole specimen (fragmented) **B** Anterior-most fragment **C** Posterior-most fragment. Scale bars: 1 mm (**A, B, C**).

The confused taxonomic history of *Ammotrypanella* and its type species, *Ammotrypanella arctica* McIntosh, 1878 has been summarized by Parapar et al. (2011) and attributed to the short description and drawings provided by McIntosh (1878). Støp-Bowitz (1945) proposed that *Ammotrypanella* should be considered as synonymous with *Ophelina*, while Fauchald (1977) treated *Ammotrypanella* as a valid genus characterized by having the branchiae limited to the posterior part of body. Schüller (2008) provided a re-diagnosis of *Ammotrypanella*, following the examination of the type material of *A. arctica*, and while she pointed out that the holotype (BMNH.1921.1.2392) is in a poor state, she confirmed the presence of branchiae in the posterior part of the body only. Based on this observation Schüller (2008) then provided descriptions of three new species from abyssal Southern Ocean (*A. cirrosa*, *A. mcintoshi* and *A. princessa*), bringing the currently valid number of *Ammotrypanella* species to four. The holotype of *A. arctica* has also been examined as part of this study (Fig. 5A–C) but is in too poor condition (now in three fragments) to provide meaningful information.

As a taxonomic revision is beyond the scope of this study, we follow the definition of *Ammotrypanella* given by Schüller (2008), with one amendment. Schüller (2008) considered that anal tube may be absent, while here we suggest that it was likely missing due to damage.

##### Diagnosis.

Body long and thin, with ventral groove along whole length of body. Prostomium bluntly rounded to conical with small palpode, peristomium indistinct. Eyes absent. Parapodia embedded into lateral groove in median region, becoming more distinct in posterior region. Parapodia with branchiae in third quarter of body. All chaetae simple. Branchiae flat, wide at base, tapering to top. Anal tube present.

Several morphotypes with branchiae restricted to the posterior part of the body were encountered in the UKSR material, which is consistent with genus *Ammotrypanella* as discussed above. The UKSR-collected species can be distinguished from four known species assigned to this genus mainly by the form of anal tube:

*Ammotrypanella arctica* McIntosh, 1878 has an elongated anal tube about same length as posterior abranchiate region and provided with a deciduous anal cirrus and terminal anus (see Schüller et al. 2008; Parapar et al. 2011).

*Ammotrypanella cirrosa* Schüller, 2008 has an elongated anal tube, its length equals to length of last 5–8 chaetigers, posterior margin with numerous cirri.

*Ammotrypanella mcintoshi* Schüller, 2008 lacks an anal tube. Although the absence of an anal tube was considered real and a distinguishing feature of this species by Schüller (2008), it is not clear if the anal tube was in fact missing (fallen off) (see comment in Parapar et al. 2011).

*Ammotrypanella princessa* Schüller, 2008 has a prostomium which mimics the shape of a royal crown (Schüller 2008).

Additionally, *Ophelina opisthobranchiata* Wirén, 1901 described from the deep sea of Spitsbergen, also has a posterior distribution of branchiae. In his recent re-description Kongsrud et al. (2011) preferred not to recognize this species as *Ammotrypanella* due to lack of phylogenetic analysis and variation of morphology in *Ophelina*.

Our molecular analysis revealed the presence of four distinct CCZ species, forming a well-supported clade. Three of those species (*Ammotrypanella keenani* sp. nov., *Ammotrypanella kersteni* sp. nov. and *Ammotrypanella* sp. NHM_1653) are represented by reasonably well-preserved specimens. Unfortunately, species NHM_2114 is represented by a single specimen with all branchiae now lost and it is therefore assigned to this genus only based on molecular data.

#### Ammotrypanella keenani sp. nov.

Taxon classificationAnimaliaCapitellidaCapitellidae

30C7978C-817C-5E17-8BC2-0C19EFD872B9

http://zoobank.org/BF6FB02D-DF8E-4781-A76D-9B209FCC086B

Fig. 6A–J

##### Material examined.

NHM_1166C NHMUK ANEA 2019.7109, coll. 26 Feb. 2015, 12°06.93N, 117°09.87W, 4100 m http://data.nhm.ac.uk/object/483c6faa-0338-4cf5-a21d-6f448c72f4aa; NHM_1250 **(holotype**) NHMUK ANEA 2019.7110, coll. 01 Mar. 2015, 12°15.44N, 117°18.13W, 4302 m http://data.nhm.ac.uk/object1514d25d-b485-4b90-8981-3e84381bf250; NHM_1871 (**paratype**) NHMUK ANEA 2019.7111, coll. 13 Mar. 2015, 12°02.49N, 117°13.03W, 4094 m http://data.nhm.ac.uk/object/d93680b5-a3a3-4623-afc4-17062e1a1a58; NHM_1949 NHMUK ANEA 2019.7112, coll. 14 Mar. 2015, 12°11.406N, 117°22.282W, 4182 m http://data.nhm.ac.uk/object/cff2696d-06ab-42a1-843e-6ef894872f32.

##### Type locality.

Pacific Ocean, CCZ, 12°15.44N, 117°18.13W, depth 4302 m, in mud between polymetallic nodules.

##### Description.

This is a small to medium-sized species (6–16 mm long), represented by four specimens. NHM_1250 and NHM_1871 are complete specimens in good condition 12 mm long and 0.8 mm wide for 38 chaetigers and 16 mm long and about 1 mm wide respectively for 38 chaetigers. NHM_1166C and NHM_1949 are complete, but much smaller specimens in poor condition, 6–7mm long, with mid-body region twisted and damaged, therefore the exact number of chaetigers cannot be established, but at least 34 chaetigers observed in both specimens.

**Figure 6.**
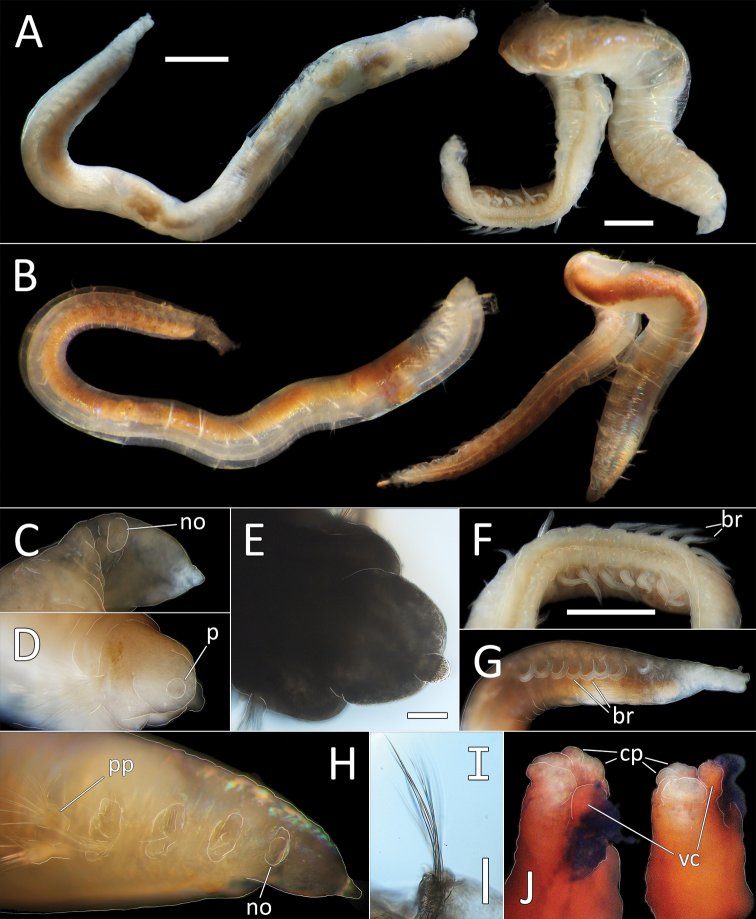
*Ammotrypanella keenani* sp. nov. **A** Lab images, whole specimens (holotype [specimen NHM_1250], post-staining, faded stain [left], paratype NHM_1871, unstained [right]) **B** Live images, whole specimens (holotype [left], paratype NHM_1871 [right]) **C** Lab image, prostomium (paratype NHM_1871, damaged, no = everted nuchal organ) **D** Lab image, prostomium (holotype, p = palpode) **E** Lab image, detail of palpode (holotype) **F** Lab image, posterior branchiae (paratype NHM_1871, br = branchiae) **G** Lab image, posterior branchiae (holotype, br = branchiae) **H** Live image, anterior (paratype NHM_1871, pp = parapodia, no = nuchal organ) **I** Lab image, detail of capillary chaetae (holotype) **J** Lab image, detail of anal funnel, ventro-distal (left) and latero-distal (right) views (holotype, stained, cp = cushioned pad, vc = ventral cirrus). Morphological features in plates **C–H, J** have been outlined with a fine white line to improve clarity of those features. Scale bars: 1 mm (**A, F,G**); 100 μm (**E, I**).

Body cylindrical, iridescent and smooth, no annulation detectable. Ventral groove along the entire body length. Preserved specimen pale yellow in ethanol (Fig. 6A). Live specimens translucent with orange gut (Fig. 6B). First 5–8 and posterior (branchial and 7 postbranchial) chaetigers crowded, chaetigers in midbody elongated.

Prostomium conical with distinct, slightly elongated palpode (NHM_1871, NHM_1166C) (Fig. 6C, H) or broad with short, button-like palpode (Fig. 6D, E). (NHM_1250, NHM_1949). Nuchal organs everted in NHM_1871, not pigmented (Fig. 6C, H).

Branchiae present, but limited to posterior region only, where at least 10 or 11 pairs present in chaetigers 22(23)–32, but only seven pairs were observed in smaller specimens (NHM_1166C and NHM_1949). All branchiae cirriform; first two pairs observed in NHM_1781 reduced in size, with the first pair (ch. 22) smallest (Fig. 6F); first (ch. 23) and last pair observed in NHM_1250 reduced in size (Fig. 6G); all branchiae of similar size in NHM_1166C and NHM_1949.

Parapodia distinct, biramous; observed as broad lobes in chaetigers 1–5 (Fig. 6H), becoming smaller in subsequent chaetigers; parapodia embedded in distinct lateral grooves. Chaetae are capillaries (Fig. 6I), anterior 5–8 crowded chaetigers with numerous chaetae in bundles, fewer chaetae in following chaetigers; chaetae longest in the anterior crowded chaetigers.

Anal tube the length of about half of the length of abranchiate posterior region, elongated, cylindrical; distal end with circlet of about four tightly packed cushion-like pads and thickened ventral pad observed in specimen NHM_1250 (damaged in other specimens) (Fig. 6J), ventral cirrus not observed.

##### Genetic data.

GenBank MN217408-MN217411 for 16S, MN217491 for 18S and MN217513–MN217514 for COI. This species is genetically identical or very similar to COI sequences attributed to “Opheliidae sp. 2” in Janssen et al. (2015) collected in the German and French exploration contract areas, with K2P values ranging from 0.0–0.008 between *A. keenani* sp. nov. and specimens with accession numbers KJ736399–KJ736403. In our phylogenetic analyses *A. keenani* sp. nov. is basal in a well-supported clade containing the three other *Ammotrypanella* species from this study.

##### Remarks.

Posterior distribution of branchiae and variously preserved cylindrical tube was observed in all specimens examined, irrespective of their size. These specimens represent one of several species consistent with genus *Ammotrypanella* recognized from the UKSR material. This species is most similar to *Ammotrypanella* sp. NHM_1653 in the relatively small body size and possession of an elongated (cylindrical) anal tube. This species can be distinguished from other *Ammotrypanella* material in this study and known *Ammotrypanella* species in having an anal tube distally with 4 or 5 cushion-like pads rather than distinct cirri, although this observation is based on only single specimen.

##### Ecology.

Found in the eastern part of polymetallic nodule province in the CCZ.

##### Etymology.

Named in honor of Edward Keenan, boatswain onboard RV Melville on the AB01 ABYSSLINE cruise in 2013.

#### Ammotrypanella kersteni sp. nov.

Taxon classificationAnimaliaCapitellidaCapitellidae

8419C805-2D47-51FA-8950-ACC85C84D033

http://zoobank.org/79227777-2043-4206-AF84-B51FF7293231

Figs 7A, B , 8A–F

##### Material examined.

NHM_254 (**holotype**) NHMUK ANEA 2019.7107, coll. 17 Oct. 2013, 13°45.21N, 116°29.12W, 4128 m http://data.nhm.ac.uk/object/3441cd68-7432-4dee-8415-966b104c3077.

##### Type locality.

Pacific Ocean, CCZ, 13°45.21N, 116°29.12W, depth 4128 m, in mud between polymetallic nodules.

##### Description.

**Figure 7.**
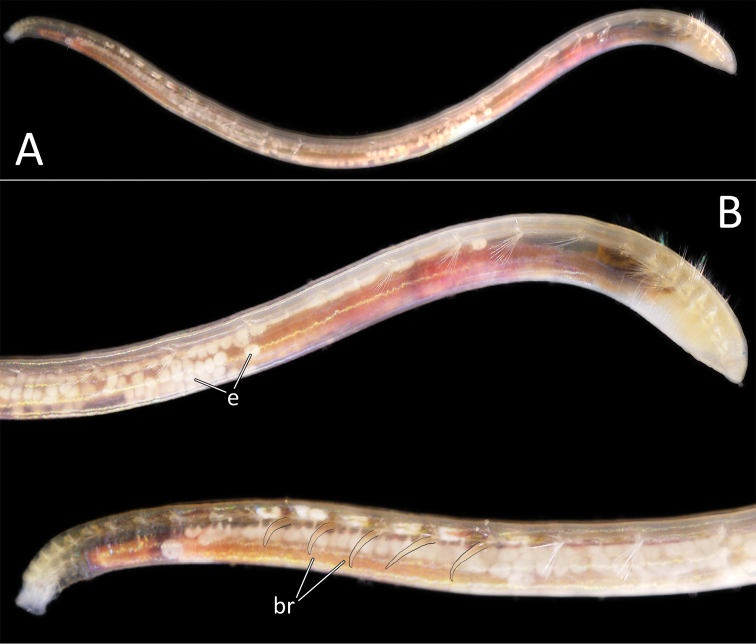
*Ammotrypanella kersteni* sp. nov. holotype (specimen NHM_254) live images. **A** Live image, whole specimen **B** Live image, detail of anterior (upper) and posterior (lower), with branchiae outlined in a fine black line (e = eggs, br = branchiae).

**Figure 8.**
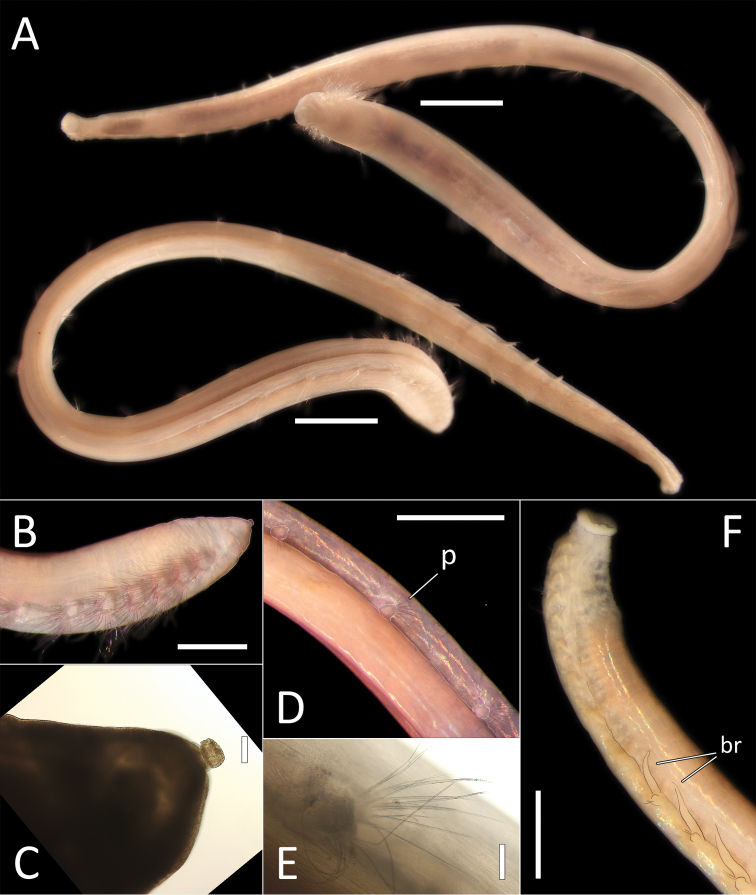
*Ammotrypanella kersteni* sp. nov. holotype (specimen NHM_254). **A** Lab images, whole specimens, dorsal (upper) and ventral (lower) **B** Lab image, anterior **C** Lab image, detail of palpode **D** Lab image, mid-body parapodia (p = parapodium) **E** Lab image, detail of parapodium **F** Lab image, posterior and anal funnel. Morphological features in plates **B, D, F** have been outlined with a fine white or black line to improve clarity of those features. Scale bars: 2 mm (**A**); 1 mm (**B, D, F**); 100 μm (**C, E**).

This species is represented by a single specimen in very good condition, although now split into two fragments, following tissues sampling for molecular analysis. Specimen (when complete) 31 mm long and 1.5 mm wide for 36 chaetigers. Body cylindrical, iridescent and smooth, no annulation detectable (Fig. 7). Ventral groove along the entire body length. Preserved specimen pale yellow in ethanol (Fig. 8). First seven and posterior (branchial and last six post branchial) chaetigers crowded, chaetigers in midbody elongated.

Prostomium of preserved specimen oval and broad (about as long as wide) and anteriorly blunt, somewhat truncated and bearing very distinct short, button-like palpode (Fig. 8C). Nuchal organs observed as narrow slits laterally on posterior part of prostomium.

Branchiae present, but limited to posterior region only, where present in chaetigers 22–28, seven pairs. All branchiae cirriform, of similar length, with red pigment in live specimen (Fig. 7B).

Parapodia distinct, biramous; observed as a broad lobe in chaetigers 1–7 (Fig. 8B), becoming smaller in subsequent chaetigers; parapodia embedded in distinct lateral grooves. Chaetae are capillaries (Fig. 8E), first seven chaetigers with numerous chaetae in bundles, fewer chaetae in following chaetigers; chaetae long, becoming progressively longer over the first seven chaetigers and then getting progressively shorter towards posterior part of the body.

Posterior achateous end (it is unclear if it represents anal tube) the length of two posterior chaetigers, a funnel-shaped structure with broad distal opening, distal margin smooth (Fig. 8F).

##### Reproductive information.

Ovigerous specimen with eggs of 200–250 µm in size clearly observed in mid through to posterior part of the body (Fig. 7B).

##### Genetic data.

GenBank MN217412 for 16S, MN217492 for 18S and MN217515 for COI. No identical matches on GenBank for COI, 16S or 18S. This taxon does not match any previous COI sequences, and we only have one specimen from the current study, which may indicate that this represents a rare species. In our phylogenetic analyses it forms a monophyletic clade with *Ammotrypanella keenani* sp. nov., *Ammotrypanella* sp. (NHM_2114) and *Ammotrypanella* sp. (NHM_1653) (Fig. 23).

##### Remarks.

*Ammotrypanella princessa* Schüller, 2008 is most similar to our species because of the shape of prostomium; however, this may be a preservation artefact (see earlier comments), which mimics the shape of a royal crown (Schüller 2008). However, *A. princessa* is a much smaller species (5–11 mm long) with fewer body segments (33–35).

The anal tube commonly becomes detached in opheliids and when short anal tubes have been described in the past, it is important to be mindful that the anal tube may in fact be missing. The posterior achateous end in UKSR species is rather short, but it appears to have a distinct form, and therefore we suggest it may possibly represent anal tube rather than damaged posterior end. However, other *Ammotrypanella* species possess an elongated cylindrical anal tube, which could suggest that the anal tube in *A. kersteni* sp. nov. is in fact missing. At the same time, the anal tubes of Opheliidae species show a variety of forms, and it is not impossible to speculate that similar variability can be found in *Ammotrypanella* as more species are discovered. With the current evidence based on single specimen we cannot clarify if the funnel-shaped posterior end represents the anal tube.

##### Ecology.

Found in the eastern polymetallic nodule province of the CCZ.

##### Etymology.

Named in honor of Oliver Kersten, member of the science party of both ABYSSLINE cruises.

#### Ammotrypanella

Taxon classificationAnimaliaCapitellidaCapitellidae

sp. (NHM_1653)

CB18775D-211B-504D-9AB0-D762D692ADDE

Fig. 9A–H

##### Material examined.

NHM_1653 NHMUK ANEA 2019.7108, coll. 10 Mar. 2015, 12°21.81N, 116°40.86W, 4233 m http://data.nhm.ac.uk/object/a2f7ed04-7275-4a57-a058-bd750cacc715.

##### Description.

**Figure 9.**
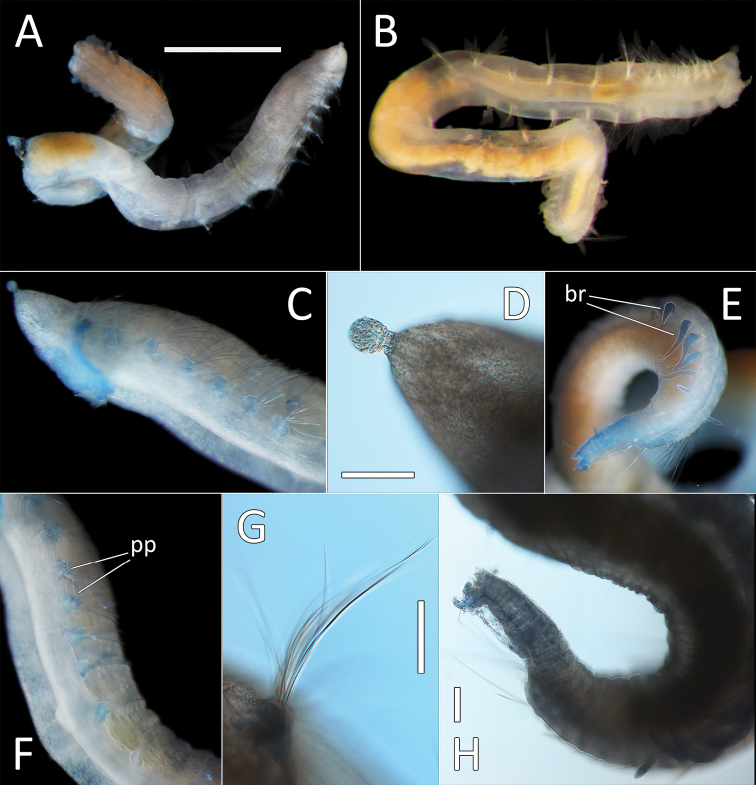
*Ammotrypanella* sp. NHM_1653 (specimen NHM_1653). **A** Lab image, whole specimen (faded stain **B** Live image, whole specimen **C** Lab image, anterior (faded stain) **D** Lab image, detail of palpode **E** Lab image, posterior (faded stain, br = branchiae) **F** Lab image, anterior-midbody (faded stain, pp = parapodia) **G** Lab image, detail of capillary chaetae **H** Lab image, detail of posterior and anal tube. Morphological features in plates **C, E, F, G** have been outlined with a very fine white to improve clarity of those features. Scale bars: 1 mm (**A**); 100 μm (**D, G, H**).

This small species is represented by a single complete specimen in reasonable condition, except for some damage to anal tube (Fig. 9). Complete specimen 7.5 mm long and 0.5 mm wide for 34 chaetigers. Body cylindrical, with slight annulation detectable. Ventral groove along the entire body length. Preserved specimen pale pink in ethanol; live specimen semi-translucent with orange gut (Fig. 9B). First seven and posterior (branchial and last six postbranchial) chaetigers crowded, chaetigers in midbody elongated.

Prostomium conical (longer than wide) anteriorly tapering into blunt tip and bearing very distinct, round palpode (Fig. 9C, D). Nuchal organs observed as narrow, lightly pigmented slits, laterally on posterior part of prostomium.

Branchiae present, but limited to posterior region only, where present in chaetigers 22–28, seven pairs. All branchiae cirriform; large, of similar length except for the last branchial pair, which is reduced (Fig. 9E).

Parapodia distinct, biramous; observed as a small lobe in chaetigers 1–7, becoming smaller in subsequent chaetigers; parapodia embedded in distinct lateral grooves (Fig. 9F). Chaetae are capillaries (Fig. 9G), first seven chaetigers with numerous chaetae in bundles, fewer chaetae in following chaetigers; chaetae long in first seven and last six chaetigers, shorter in midbody.

Anal tube the length of three posterior chaetigers (Fig. 9H); cylindrical, distally slightly narrowing; due to some damage, the form of distal end cannot be established with certainty; short thick ventral cirrus attached near the distal end.

##### Genetic data.

GenBank MN217413 for 16S, MN217493 for 18S and MN217516 for COI. This species is genetically identical or very similar to COI sequences collected in the German and French exploration contract areas and published in Janssen et al. (2015), with K2P values ranging from 0.0–0.002 between *Ammotrypanella* sp. (NHM_1653) and the already published sequences with accession numbers KJ736387–KJ736392. In our phylogenetic analyses is forms a monophyletic clade with *Ammotrypanella keenani* sp. nov., *A. kersteni* sp. nov. and *Ammotrypanella* sp. (NHM_2114) (Fig. 23).

##### Remarks.

This is another species with branchiae limited to the posterior end consistent with the genus *Ammotrypanella*. While this species is similar to *Ammotrypanella kersteni* sp. nov., it can be clearly distinguished from it by a much smaller body size, shape of prostomium and bearing narrow, elongated, cylindrical anal tube. This form of tube is however similar to other known anal tube-bearing species of *Ammotrypanella* and due to some damage to this feature in the UKSR specimen, its form cannot be established with certainty.

##### Ecology.

Found in polymetallic nodule province.

#### Ammotrypanella

Taxon classificationAnimaliaCapitellidaCapitellidae

sp. (NHM_2114)

B31D2D76-C76D-538B-B561-B04C5533015F

Fig. 10A–E

##### Material examined.

NHM_2114 NHMUK ANEA 2019.7113, coll. 20 Mar. 2015, 19°27.874N, 120°01.525W, 4026 m, http://data.nhm.ac.uk/object/f4492dd1-8088-47c6-97d9-32e43ae99552.

##### Description.

**Figure 10.**
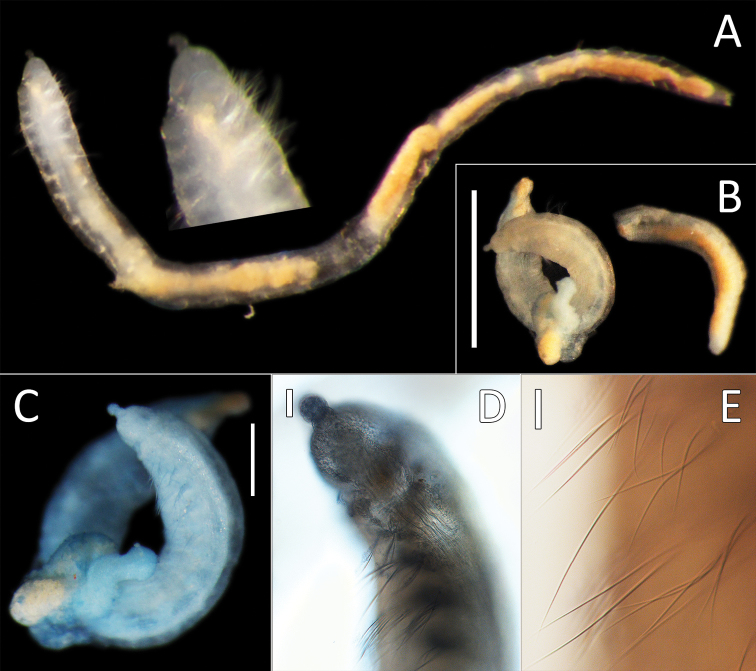
*Ammotrypanella* sp. NHM_2114 (specimen NHM_2114). **A** Live images, whole specimen with detail of anterior **B** Lab images, fragmented whole specimen, post-staining, (faded stain, anterior fragment [left], posterior fragment [right],) **C** Lab image, anterior fragment, post-staining, faded stain **D** Lab image, detail of anterior and palpode **E** Lab image, detail of capillary chaetae. Scale bars: 1 mm (**B**); 250 μm (**C**) 50 μm (**D**); 25 μm (**E**).

Single, minute, damaged specimen; now with posterior part of the body removed for molecular analysis. Anterior fragment 1.6 mm long 0.2 mm wide for about 16 chaetigers (chaetae observed on only 11 of these, the rest of the fragment damaged with chaetae missing). Ventral groove along the entire length of the fragment. First six chaetigers crowded. Preserved specimens pale yellow in ethanol; live specimen translucent with orange gut (Fig. 10A). Prostomium broad (slightly longer than wide) and anteriorly bluntly rounded, bearing distinct bi-articulated palpode, with globular distal articulation (Fig. 10B–D). Parapodia distinct, biramous; as a small lobe in chaetigers 1–9, becoming indistinct in the rest of the fragment. All chaetae observed simple capillaries (Fig. 10E).

##### Additional morphological observations from live specimen.

Upon collection the live specimen was imaged and appears to be complete. Presence and distribution of branchiae cannot be established from the image. The anal tube was probably missing upon collection of the specimen.

##### Genetic data.

GenBank MN217414 for 16S and MN217494 for 18S. COI was unsuccessful for this specimen, no identical GenBank matches for 16S or 18S. *Ammotrypanella* sp. (NHM_2114) cluster with the *Ammotrypanella keenani* sp. nov., *Ammotrypanella* sp. (NHM_1653) and *Ammotrypanella kersteni* sp. nov. in our phylogenetic analyses (Fig. 23).

##### Remarks.

Although important diagnostic features cannot be fully confirmed in this specimen, in the phylogenetic tree it falls into a well-supported clade containing *Ammotrypanella* species and likely represents another species in this genus reported from UKSR material. At present, morphological information obtained from this single representative is limited which prevent us from providing a formal description.

#### Ophelina

Taxon classificationAnimaliaCapitellidaCapitellidae

Ørsted, 1843

55273292-CC95-538A-AE37-FB58128D5207

##### Notes.

Ørsted (1843) erected *Ophelina* for *O. acuminata* from Danish coasts. It is now represented by around 60 species (Read and Fauchald 2019), although the numbers vary according to different workers due to its confused taxonomic history (Maciolek and Blake 2006; Parapar et al. 2011). *Ophelina* is the most diverse genus of the family Opheliidae, although it likely represents a paraphyletic grouping (Law et al. 2014). This genus is represented in UKSR-collected material by 11 species as revealed by molecular analysis.

The diagnosis of *Ophelina* presented here follows that given by Maciolek and Blake (2006).

##### Diagnosis.

Body elongate, with deep ventral groove and two lateral grooves along entire length of body. Prostomium conical, sometimes with terminal palpode; eyes present or absent. Branchiae present or absent; if present, beginning on chaetiger 2, continuing to posterior end, sometimes absent from middle or far posterior chaetigers; branchiae single, cirriform. Segmental lateral eyes absent. Noto- and neuropodia with small fascicles of capillary chaetae; small ventral cirrus present. Pygidium with anal funnel sometimes bearing long unpaired cirrus and additional lateral cirri.

#### Ophelina curli sp. nov.

Taxon classificationAnimaliaCapitellidaCapitellidae

6B05C180-8584-5BA3-AFC7-E99A5B6FC21A

http://zoobank.org/0929D0E7-E391-42F9-B7CE-D3034C7F70FB

Fig. 11A–H

##### Material examined.

NHM_2112 (**holotype**) NHMUK ANEA 2019.7131, coll. 20 Mar. 2015, 19°27.874N, 120°01.525W, 4026 m, http://data.nhm.ac.uk/object/c1554f01-2324-4d8d-b775-dca42f5918e7.

##### Type locality.

Pacific Ocean, CCZ, 19°27.874N, 120°01.525W, depth 4026 m, in mud between polymetallic nodules.

##### Description.

This species is represented by a single specimen 30 mm long and 1 mm wide for 28 chaetigers.

**Figure 11.**
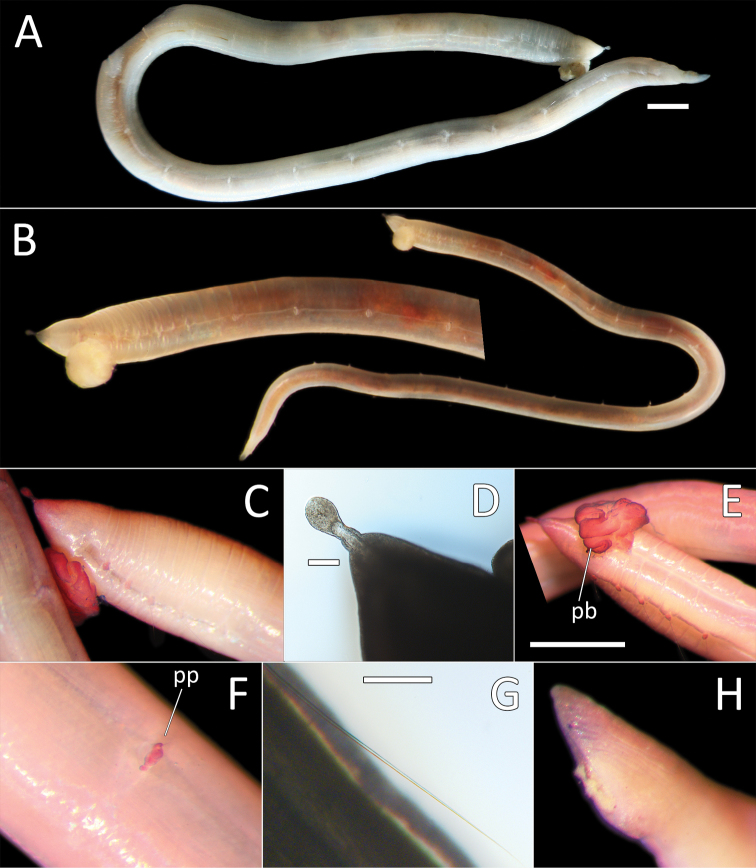
*Ophelina curli* sp. nov. holotype (specimen NHM_2112). **A** Lab image, whole specimen (post-staining, very faded stain) **B** Live image, with detail of anterior **C** Lab image, anterior and proboscis, lateral view, stained **D** Lab image, detail of palpode **E** Lab image, anterior and proboscis, ventral view (stained, pb = proboscis) **F** Lab image, mid-body parapodium (stained, pp = parapodia) **G** Lab image, detail of capillary chaeta **H** Lab image, anal funnel. Scale bars: 1 mm (**A, E**); 100 μm (**D, G**)

Ventral and lateral grooves distinct along whole length of body. Colour in alcohol yellow to light tan (Fig. 11A); live specimen semi-translucent, with red gut (Fig. 11B). Body very smooth, iridescent, segmental furrows and annulations indistinct (best observed in the anterior-most chaetigers). First five chaetigers slightly crowded, the subsequent chaetigers elongated.

Prostomium conical (longer than wide), with distinct, tear-shaped terminal palpode (Fig. 11C, D). Eyes not observed. Nuchal organs observed as slits, laterally on posterior part of prostomium; without pigmentation. Proboscis fully everted; dorso-ventrally flattened multilobed structure with ventral groove (Fig. 11E).

Branchiae absent. Parapodia biramous, embedded in lateral grooves; parapodia small conical lobes, best observed on anterior seven chaetigers; no distinct pre- or postchaetal lobes observed (Fig. 11F).

Chaetae all slender, smooth capillaries (Fig. 11G), very few in both rami. Notochaetae mostly longer than neurochaetae.

Anal tube attached; narrow and smooth; no cirri observed (Fig. 11H). The proximal half cylindrical; distal half (in ventral view) as flattened sheath (if opened up), but observed with lateral margins curled up, ventrally slit; distal half (in lateral view) distinctly asymmetrical with dorsal margin longer than ventral one.

##### Genetic data.

GenBank MN217435 for 16S and MN217502 for 18S. COI was unsuccessful for this specimen, no identical GenBank matches for 16S or 18S. *Ophelina curli* sp. nov. is sister to *Ophelina juhazi* sp. nov. in our phylogenetic analyses (Fig. 23).

##### Remarks.

Morphologically this species is very similar to *Ophelina nematoides* (Ehlers, 1913) and to UKSR *Ophelina juhazi* sp. nov. in being abranchiate and having 28–30 chaetigers. Two other abranchiate species that are morphologically similar to *Ophelina abranchiata* Støp-Bowitz, 1948 are also reported in this material, but these differ in having much smaller body size (4.5–8 mm) and having only 17 or 18 chaetigers.

Ehlers (1913) provided description (but no drawings) of *O. nematoides* (as *Ammotrypane nematoides*) based on specimens from 2725 m depth in the Indian Ocean sector of the Southern Ocean (65°32'0"S, 85°30'0"E). Other reports from the Southern Ocean have been shallower (Read and Fauchald 2019). Brief diagnosis based on translation of Ehlers (1913): “Specimen 30 mm long and 1 mm wide, with 30 chaetigers. Conical prostomium with palpode. No branchiae. Basal segment (= anal tube?) appears segmented in a brightened condition is a thick sheet, when stretched, of the length of the last two segments, the margins of which are curled against each other to form on the ventral surface the narrow entrance to a gutter emanating at the back. I did not see any attachments or papillae.”

The main difference between *O. nematoides* and *Ophelina curli* sp. nov. is the number of chaetigers, 30 in the former versus 28 in the latter. The shape of anal tube appears to be similar, but without drawing or access to Ehlers’ type specimen, this structure cannot be meaningfully compared using Ehlers’ description alone.

The morphologically similar species *Ophelina juhazi* sp. nov. also found in the UKSR material can be distinguished by its smaller size, 17 mm compared to the 30 mm *O. curli* sp. nov., and the shape of anal tube, which in *O. juhazi* sp. nov. is cylindrical throughout, entire (no ventral slit), distally slightly narrowing and symmetrical.

##### Ecology.

Found in polymetallic nodule province of the eastern CCZ.

##### Etymology.

Named in honor of Cassidy Curl, Ordinary Seaman onboard RV Melville on the AB01 ABYSSLINE cruise in 2013.

#### Ophelina ganae sp. nov.

Taxon classificationAnimaliaCapitellidaCapitellidae

DBD967D4-2883-59F4-A0E9-EF7E519F2AF8

http://zoobank.org/4B05DE1F-CD07-40CF-A87D-9BC1B45F5477

Fig. 12A–I

##### Material examined.

NHM_245 NHMUK ANEA 2019.7140, coll. 16 Oct. 2013, 13°48.70N, 116°42.60W, 4076 m http://data.nhm.ac.uk/object/3a34b9cb-504b-48a3-a8e9-93077ec69520; NHM_248 NHMUK ANEA 2019.7141, coll. 16 Oct. 2013, 13°48.70N, 116°42.60W, 4076 m http://data.nhm.ac.uk/object/e67f7724-8c9f-4463-943e-7cda20441728; NHM_473 NHMUK ANEA 2019.7142, coll. 22 Oct. 2013, 13°43.597N, 116°40.20W, 4160 m http://data.nhm.ac.uk/object/79dcab18-936b-430e-b770-6aab60d285c5; NHM_598 (SEM) (**paratype**) NHMUK ANEA 2019.7143, coll. 17 Feb. 2015, 12°23.174N, 116°32.92W, 4202 m http://data.nhm.ac.uk/object/2fa20a59-8bb3-4ef8-b2e9-efccbe2c9414; NHM_708 NHMUK ANEA 2019.7144, coll. 20 Feb. 2015, 12°32.23N, 116°36.25W, 4425 m http://data.nhm.ac.uk/object/2077c6c6-0e3e-4dfa-97a0-16d6c386ff07; NHM_1098 NHMUK ANEA 2019.7145, coll. 26 Feb. 2015, 12°06.93N, 117°09.87W, 4100 m http://data.nhm.ac.uk/object/5661fb64-83a2-4e9a-b3c3-a8405705ed1a; NHM_1137 (**holotype**) NHMUK ANEA 2019.7146, coll. 26 Feb. 2015, 12°06.93N, 117°09.87W, 4100 m http://data.nhm.ac.uk/object/11616c16-bdb5-4813-9d17-7170bb62702b; NHM_1309 (**paratype)** (SEM) NHMUK ANEA 2019.7147, coll. 01 Mar. 2015, 12°15.44N, 117°18.13W, 4302 m http://data.nhm.ac.uk/object/1ff41b52-9801-4b2e-8e01-ea34597b708d.

##### Type locality.

Pacific Ocean, CCZ, 12°06.93N, 117°09.87W, depth 4100 m, in mud between polymetallic nodules.

##### Description.

**Figure 12.**
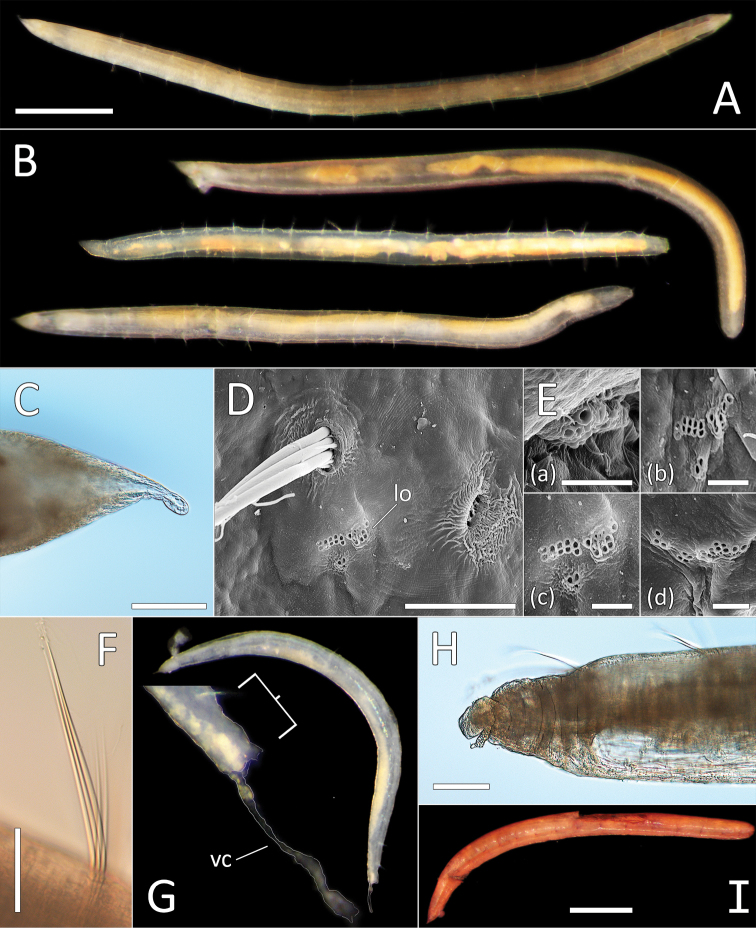
*Ophelina ganae* sp. nov. **A** Lab image, whole specimen (holotype [specimen NHM_1137] **B** Live images, whole specimens (holotype [bottom], paratype NHM_598 [middle], paratype NHM_1309 [top]) **C** Lab image, detail of palpode (holotype) **D** SEM image, second chaetiger with lateral organ (paratype NHM_1309, lo = lateral organ) **E** SEM images, lateral organs, (a) pre-chaetigerous segment, (b) chaetiger 1, (c) chaetiger 2, (d) chaetiger 17 (last chaetiger) (paratype NHM_1309) **F** Lab image, detail of capillary chaetae (holotype) **G** Live image, with detail of potential anal funnel (specimen NHM_473, vc = potential ventral cirrus). **H** Lab image, detail of posterior (holotype) **I** Lab image, whole specimen (paratype NHM_598, stained). Morphological features in plates **G, H** have been outlined with a fine white or black line to improve clarity of those features. Scale bars: 1 mm (**A**); 100 μm (**C**); 20 μm (**D**); 5 μm (**E**); 50 μm (**F**); 100 μm (**H**); 1 mm (**I**).

Small species (4.5–8 mm long without anal tube), represented by eight specimens; all preserved specimens without anal tube (likely missing due to damage). Holotype is 7 mm long and 0.3 mm wide for 17 chaetigers. Paratypes 7–8 mm long and 0.33–0.35 mm wide for 17 chaetigers. Body cylindrical and smooth without distinct annulation. Preserved specimen yellow in ethanol (Fig. 12A); live specimens translucent (Fig. 12B, G). Ventral and lateral grooves distinct along whole length of body. Anterior and posterior-most chaetigers slightly shorter than mid-body chaetigers.

Prostomium elongate, conical with small acute terminal palpode (Fig. 12C). Eyes not observed. Nuchal organs rounded, laterally on posterior part of prostomium. Peristomium indistinct. Anterior prechaetigerous region of body with three elongated achaetous segments, detected upon observation of three lateral organs under SEM; lateral organs detected in all chaetigers under SEM (Fig. 12D, E). Parapodia rudimentary, biramous, embedded in lateral grooves; no distinct pre- or postschaetal lobes.

Chaetae all slender, smooth capillaries (Fig. 12F), appear in small numbers in both rami, often broken off entirely. Notochaetae mostly longer than neurochaetae, extremely short chaetae observed in most fasciles under SEM.

Branchiae absent. Anal tube not observed in preserved specimens (e.g. Fig. 12H), but possibly captured in image of “live” specimen NHM_473 (Fig. 12G). Observed anal tube cylindrical, relatively short (only slightly longer than wide); with very long thin ventral cirrus attached subdistally on ventral side of anal funnel. Stained specimens without distinct pattern (Fig. 12I).

##### Genetic data.

GenBank MN217436-MN217442 for 16S, MN217503 for 18S and MN217521-MN217523 for COI. In our phylogenetic analyses, *Ophelina ganae* sp. nov. is sister to Ophelina cf. abranchiata (NHM_1769) and form a well-supported clade with this species and Ophelina cf. abranchiata (NHM_2017) together with at least two other abranchiate species (Fig. 23).

##### Remarks.

Molecular analysis of the UKSR-collected material revealed presence of three distinct small abranchiate species that morphologically resemble *Ophelina abranchiata*. Given the taxonomic problems of this species, the challenge is not only to morphologically distinguish these species from each other, but also from *O. abranchiata*. Here, we restrict the definition of *O. abranchiata* to that provided by Kongsrud et al. (2011) based on re-description of holotype and material from the North Sea as consistently possessing 18 chaetigers only. Therefore, the new species is differentiated from *O. abranchiata* by possessing 17 chaetigers only in all observed specimens. Furthermore, the photograph of live specimen (Fig. 12G) suggests possible presence of much shorter anal tube compared to elongated anal tubes observed in *O. abranchiata*. This would represent another distinguishing character from the known species. We believe this structure to represent anal tube as it follows the chaetigerous regions of the body (i.e. the body is not interrupted immediately after the last chaetiger as it is common in *O. abranchiata* when the anal funnels are missing). There also appear to be very long thin cirrus attached subdistally on ventral side and such structure is present in anal tubes of *Ophelina abranchiata*. However, this conclusion is only tentative given the lack of anal tube in all preserved specimens of the newly described species and it is based on observation from the photograph only. See further discussion on specimens identified as Ophelina cf. abranchiata later in this text.

##### Ecology.

Found in polymetallic nodule province of the eastern CCZ.

##### Etymology.

Named in honor of Bin Qi Gan, member of the science party of the ABYSSLINE AB02 cruise onboard the RV *Thomas G. Thompson*.

#### Ophelina juhazi sp. nov.

Taxon classificationAnimaliaCapitellidaCapitellidae

F8BCAA2C-3A78-58C9-A976-9AD20E29424F

http://zoobank.org/24EFA36C-06BA-443D-8388-02981BE73D71

Fig. 13A–G

##### Material examined.

NHM_1073 (**holotype**) NHMUK ANEA 2019.7132, coll. 26 Feb. 2015, 12°06.93N, 117°09.87W, 4100 m http://data.nhm.ac.uk/object/f7330230-224b-49e7-aa80-41e8654ea087.

##### Type locality.

Pacific Ocean, CCZ, 12°06.93N, 117°09.87W, depth 4100 m, in mud between polymetallic nodules.

##### Description.

**Figure 13.**
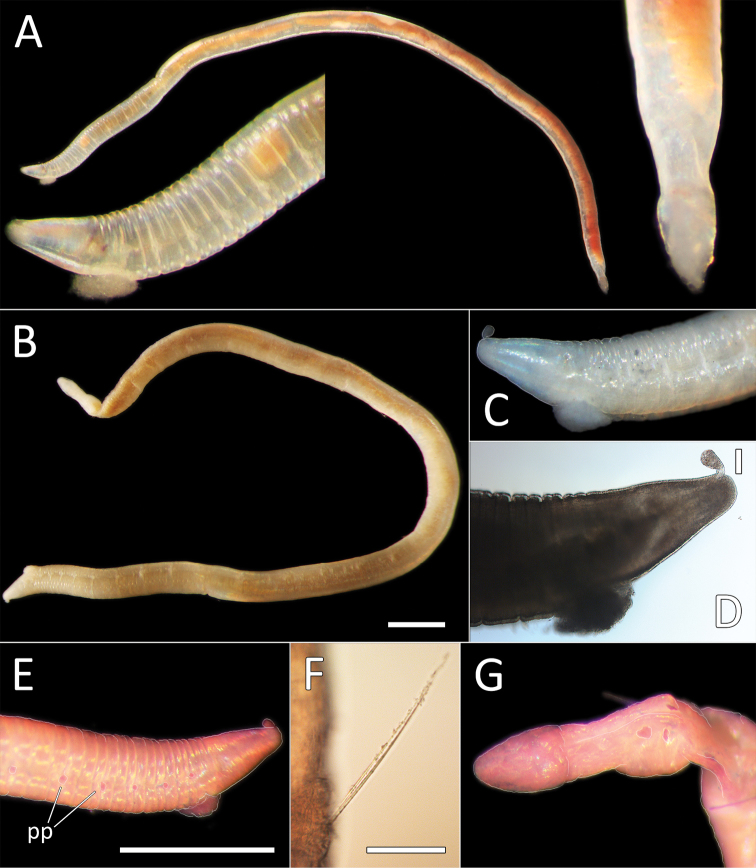
*Ophelina juhazi* sp. nov. holotype (specimen NHM_1073). **A** Live images, whole specimen (center) with detail of anterior (left) and posterior (right) **B** Lab image, whole specimen **C** Lab image, anterior and prostomium **D** Lab image, detail of palpode **E** Lab image, anterior and parapodia (stained, pp = parapodia) **F** Lab image, detail of capillary chaetae **G** Lab image, posterior and anal tube (stained). Morphological features in plates **C, E, G** have been outlined with a very fine white line to improve clarity of those features. Scale bars: 1 mm (**B**); 100 μm (**D**); 50 μm (**F**); 1 mm (**E**).

This species is represented by a single specimen 17 mm long and 0.5 mm wide for 29 chaetigers. Ventral and lateral grooves distinct along whole length of body. Live specimens translucent, with orange-red gut (Fig. 13A); colour in alcohol yellow to light tan (Fig. 13B). Body very smooth, iridescent, annulations indistinct (best observed in first five to seven anterior chaetigers). All chaetigers elongated, first five chaetigers only slightly crowded.

Prostomium conical (longer than wide), with distinct, tear-shaped terminal palpode (Fig. 13C, D). Eyes not observed. Nuchal organs observed as slits, laterally on posterior part of prostomium, without pigmentation.

Branchiae absent. Parapodia biramous, embedded in lateral grooves; parapodia small conical lobes, best observed on anterior seven chaetigers (Fig. 13E); no distinct pre- or postchaetal lobes observed. Chaetae all slender, smooth capillaries (Fig. 13F), very few in both rami. Notochaetae mostly longer than neurochaetae.

Anal tube attached; narrow, cylindrical structure, symmetrical, smooth (no cirri observed) and distally slightly narrowing (Fig. 13G).

##### Genetic data.

GenBank MN217443 for 16S and MN217504 for 18S. COI was unsuccessful for this specimen, no identical GenBank matches for 16S or 18S. In our phylogenetic analyses, *Ophelina juhazi* sp. nov. is sister to *O. curli* sp. nov. (Fig. 23).

##### Remarks.

Morphologically similar to *Ophelina curli* sp. nov. and to *Ophelina nematoides*; see Remarks under *Ophelina curli* sp. nov. for details.

##### Ecology.

Found in polymetallic nodule province of the eastern CCZ.

##### Etymology.

Named in honor of Bob Juhazi, Oiler onboard RV *Melville* on the AB01 ABYSSLINE cruise in 2013.

#### Ophelina martinezarbizui sp. nov.

Taxon classificationAnimaliaCapitellidaCapitellidae

656B5D8F-B784-5272-8ECB-263F67C9D3B4

http://zoobank.org/9D6E1A60-B5BB-48BC-A756-81845EFABFBC

Figs 14A–H , 15A–E

##### Material examined.

NHM_681 (**holotype**) NHMUK ANEA 2019.7116, coll. 20 Feb. 2015, 12°32.23N, 116°36.25W, 4425 m http://data.nhm.ac.uk/object/0de17415-a8bf-4461-a663-dea9a3e6a2b9; NHM_718 NHMUK ANEA 2019.7117, coll. 20 Feb. 2015, 12°32.23N, 116°36.25W, 4425 m http://data.nhm.ac.uk/object/f6e2fa9b-a479-4e0d-aec6-57efff6987b2; NHM_883 NHMUK ANEA 2019.7118, coll. 20 Feb. 2015, 12°34.28N, 116°36.63W, 4198 m http://data.nhm.ac.uk/object/d9a3a3b3-c16e-4359-8eb0-f09deed98401; NHM_994 NHMUK ANEA 2019.7119, coll. 24 Feb. 2015, 12°08.02N, 117°17.52W, 4122 m http://data.nhm.ac.uk/object/4f6d2b7a-169f-46a9-8b3b-5d91a021aa34; NHM_1066 NHMUK ANEA 2019.7120, coll. 26 Feb. 2015, 12°06.93N, 117°09.87W, 4100 m http://data.nhm.ac.uk/object/972f9cb1-79d7-4296-a6d6-e04543c9105c; NHM_1766 (**paratype**) NHMUK ANEA 2019.7121, coll. 11 Mar. 2015, 12°10.43N, 117°11.57W, 4045 m http://data.nhm.ac.uk/object/dc754b1c-e66b-4a58-a93e-796ebfd32f6a; NHM_1870 NHMUK ANEA 2019.7122, coll. 13 Mar. 2015, 12°02.49N, 117°13.03W, 4094 m, http://data.nhm.ac.uk/object/b6247e8d-d155-4646-87d7-e5358ada5352; NHM_2088 (SEM specimen) NHMUK ANEA 2019.7123, coll. 20 Mar. 2015-03-20, 19°27.874N, 120°01.525W, 4026 m, http://data.nhm.ac.uk/object/7dd04f2c-435b-44b1-a85f-3b05dd3014d7; NHM_2092 NHMUK ANEA 2019.7124, coll. 20 Mar. 2015, 19°27.874N, 120°01.525W, 4026 m, http://data.nhm.ac.uk/object/1a095836-fa97-48b8-ad4c-07ed28356ecb; NHM_2102 NHMUK ANEA 2019.7125, coll. 20 Mar. 2015, 19°27.874N, 120°01.525W, 4026 m, http://data.nhm.ac.uk/object/93e91313-61a3-4cd7-8221-66bf20232f14; NHM_2116 (**paratype**) NHMUK ANEA 2019.7126, coll. 20 Mar. 2015, 19°27.874N, 120°01.525W, 4026 m, http://data.nhm.ac.uk/object/d439156e-657d-4dd5-8bb5-3531e150961e; NHM_2144 NHMUK ANEA 2019.7127, coll. 20 Mar. 2015, 19°27.874N, 120°01.525W, 4026 m, http://data.nhm.ac.uk/object/79767cab-eb56-4ef1-acd0-5067ec3736de; NHM_2149 NHMUK ANEA 2019.7128, coll. 20 Mar. 2015, 19°27.874N, 120°01.525W, 4026 m, http://data.nhm.ac.uk/object/1caa9eb3-3281-4ed6-8424-dfaebcf1e20b; NHM_2150 NHMUK ANEA 2019.7129, coll. 20 Mar. 2015, 19°27.874N, 120°01.525W, 4026 m, http://data.nhm.ac.uk/object/993f577c-ee86-4660-b2d9-af0146606f92.

##### Type locality.

Pacific Ocean, CCZ, 12°32.23N, 116°36.25W, depth 4425 m, in mud between polymetallic nodules.

##### Description.

This is a medium-sized species (8–14 mm long), represented by 14 specimens.

**Figure 14.**
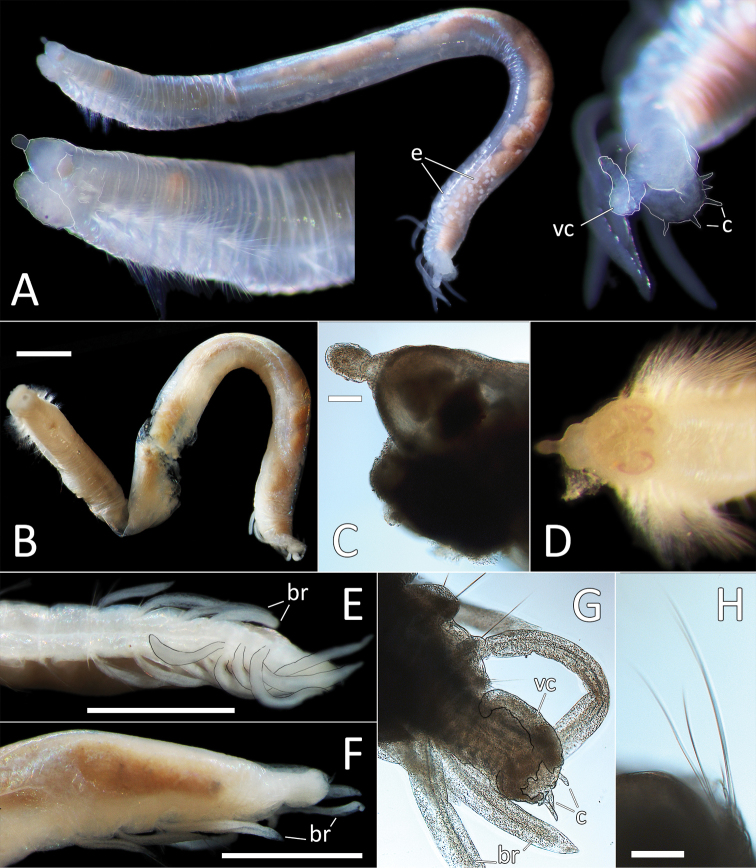
*Ophelina martinezarbizui* sp. nov. **A** Live images, whole specimen (center) with detail of anterior (left) and anal funnel (right) (holotype [specimen NHM_681], e = eggs, vc = enlarged ventral cirrus, c = cirri) **B** Lab image, whole specimen (holotype). **C** Lab image, detail of palpode (paratype NHM_2116) **D** Live image, prostomium, “royal crown” palpode (specimen NHM_2092) **E** Lab image, ventral posterior and branchiae (paratype NHM_1766, br = branchiae) **F** Lab image, lateral posterior and branchiae (paratype NHM_1766, br = branchiae) **G** Lab image, detail of anal funnel (paratype NHM_1766, vc = enlarged ventral cirri [folded over], c = cirri, br = branchiae **H** Lab image, detail of capillary chaetae (holotype). Morphological features in plates **A, E, G** have been outlined with a fine white or black line to improve clarity of those features. Scale bars: 1 mm (**B, E, F**); 100 μm (**C, H**).

**Figure 15.**
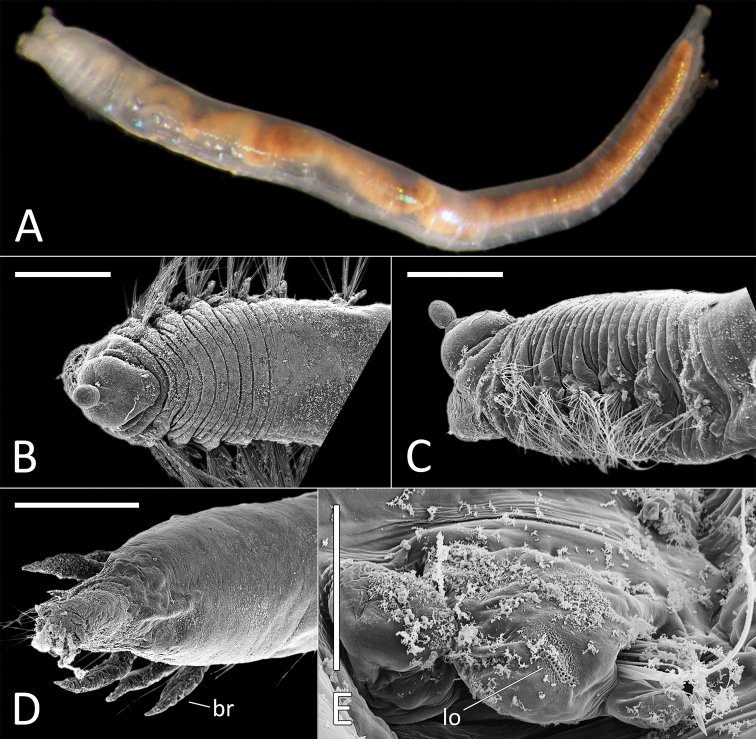
*Ophelina martinezarbizui* sp. nov. (specimen NHM_2088). **A** Live image, whole specimen **B** SEM image, dorsal anterior **C** SEM image, lateral anterior **D** SEM image, dorsal posterior, br = branchiae) **E** SEM image, detail of mid-body lateral organ (lo = lateral organ). Scale bars: 300 μm (**B, C, D**); 40 μm (**E**).

Body cylindrical, iridescent, some annulation detectable in first five to eight and last eight chaetigers, rest of body very smooth, no annulation detectable (Fig. 14A). Ventral and lateral grooves most distinct along the anterior half of the body, then less distinct. Preserved specimen yellow in ethanol (Fig. 14B); live specimens translucent, with orange gut (Figs 14A, 15A). Complete specimen with 31 chaetigers, first five to eight and last eight chaetigers crowded, chaetigers in between elongated, last six to eight chaetigers somewhat shifted ventrally.

Prostomium of all preserved specimens oval and broad (about as long as wide) and anteriorly bluntly rounded, somewhat truncated; bearing very distinct palpode, mostly short button-like sometimes distinctly bi-articulated with distal article oval in specimen NHM_2116 (Figs 14C, 15B, C). Similar form of prostomium (royal crown-shaped) can also be observed in live specimen NHM_2092 (Fig. 14D). Nuchal organs observed as slits laterally on posterior part of prostomium.

Branchiae present, with disjointed distribution in anterior and posterior chaetigers only, absent in mid-body chaetigers. Six very small (easily overlooked) branchial pairs observed consistently in chaetigers 2–7, with those on chaetigers 3–5 slightly longest. The number of attached posterior branchial pairs observed varied from one to eight pairs, with the most complete set observed in NHM_883 and NHM_1766, where eight pairs present in chaetigers 24–31 (the last chaetiger); first posterior pair small (1/2 the length of the subsequent pairs), others very long and robust in NHM_883, but all branchiae large in NHM_1766. All branchiae cirriform (Figs 14E–G, 15D).

Parapodia distinct, biramous; well developed in anterior part of the body, then becoming smaller in subsequent chaetigers. Parapodia with short rounded dorsal cirrus present; provided with a tongue-shaped lobe bearing lateral organs (observable under SEM) (Fig. 15E). Parapodia embedded in distinct lateral grooves in chaetiger 1–13, then grooves becoming less distinct. Chaetae are capillaries (Fig. 14H); first seven chaetigers with numerous chaetae in bundles, chaetae getting longer in chaetigers 2–4, being longest in chaetigers 3–5, then becoming shorter to chaetiger 13; in the posterior half of the body chaetae few and short, often missing (broken off) entirely.

Anal tube best preserved in specimen NHM_1766; anal tube relatively short (about the length of two posterior chaetigers) and thick distally asymmetrical with dorsal margin slightly longer than ventral one; distally with several short cirri, particularly on dorsal margin (Fig. 14A, G) and ventral margin with robust, short and thick ventral cirrus (Fig. 14A, G).

##### Reproductive information.

Holotype ovigerous, with eggs of roughly 100 mm size clearly observed in mid through to posterior part of the body (Fig. 14A).

##### Morphological variation.

This species is represented by the greatest number of specimens (*n* = 13) of Opheliidae species found in UKSR material. The features observed consistently are: the “royal crown”-like shape of prostomium (even in live specimens, Fig. 14D), 31 chaetigers, six pairs of tiny anterior branchiae in chaetigers 2–7. Number of attached posterior branchial pairs is variable as these are large and presumably more susceptible to damage, the exact number of posterior branchial pairs remains unknown, but the most complete observation was eight pairs in specimen NHM_883. The anal tube remains attached in all specimens, but the distal region is often damaged and ventral cirrus is often detached. The best-preserved anal tube has been observed in specimen NHM_1766 and can also be observed in the live image of the holotype.

##### Genetic data.

GenBank MN217444–MN217456 for 16S, MN217505 for 18S and MN217524–MN217531 for COI. This species is genetically identical or very similar to “*Ophelina* sp. 2” (Janssen et al. 2015), with K2P values ranging from 0.0–0.006 between *O. martinezarbizui* sp. nov. and the already published sequences with accession numbers KJ736369–KJ736370 and KJ736372–KJ736377. In our phylogenetic analyses this species is sister to *Ophelina meyerae* sp. nov. (Fig. 23).

##### Remarks.

This species superficially resembles *Ammotrypanella* species due to the presence of large branchiae in the posterior part of the body, but very small and easily overlooked branchiae are present in anterior chaetigers 2–7 in *Ophelina martinezarbizui* sp. nov. The presence of these very small branchiae easily distinguish this species from other *Ophelina* species encountered in UKSR-collected material, which are either abranchiate or branchiae are large (or at least easy to observe) in anterior chaetigers. *Ophelina martinezarbizui* sp. nov. represents a form with disjointed branchial distribution (see also comments under *Ophelina* sp. NHM_689 and NHM_1331), but it can be distinguished from these by the size of anterior branchiae, number of segments and form of anal funnel. *Ophelina martinezarbizui* sp. nov. also appears to have contrasting annulated and smooth body regions (Figs 14, 15).

Of the known *Ophelina* species, *O. ammotrypanella* Schüller, 2008 from the abyssal Southern Ocean shares the presence of small branchiae in anterior chaetigers and its “*Ammotrypanella*-like look” as the name suggests. However, in *O. ammotrypanella* the branchiae have a continuous distribution, being absent only in posterior quarter of the body.

##### Ecology.

Found in polymetallic nodule province of the eastern CCZ. This species is represented by 13 sequenced specimens, with potentially another 28 specimens available in material that has not been sequenced yet, making it the most abundant opheliid species in the UKSR samples.

##### Etymology.

Named in honor of Pedro Martinez Arbizu, member of the science party of the first ABYSSLINE cruise.

#### Ophelina meyerae sp. nov.

Taxon classificationAnimaliaCapitellidaCapitellidae

33D63C13-BC93-5DE9-9D27-64C713587569

http://zoobank.org/7F560FD4-73BF-4DEE-AD39-21FE5009FD90

Fig. 16A–G

##### Material examined.

NHM_1241(**holotype**) NHMUK ANEA 2019.7130, coll. 01 Mar. 2015, 12°15.44N, 117°18.13W, 4302 m http://data.nhm.ac.uk/object/920d8670-507e-4126-a42b-6e208bbe66d3.

##### Type locality.

Pacific Ocean, CCZ, 12°15.44N, 117°18.13W, depth 4302 m, in mud between polymetallic nodules.

##### Description.

**Figure 16.**
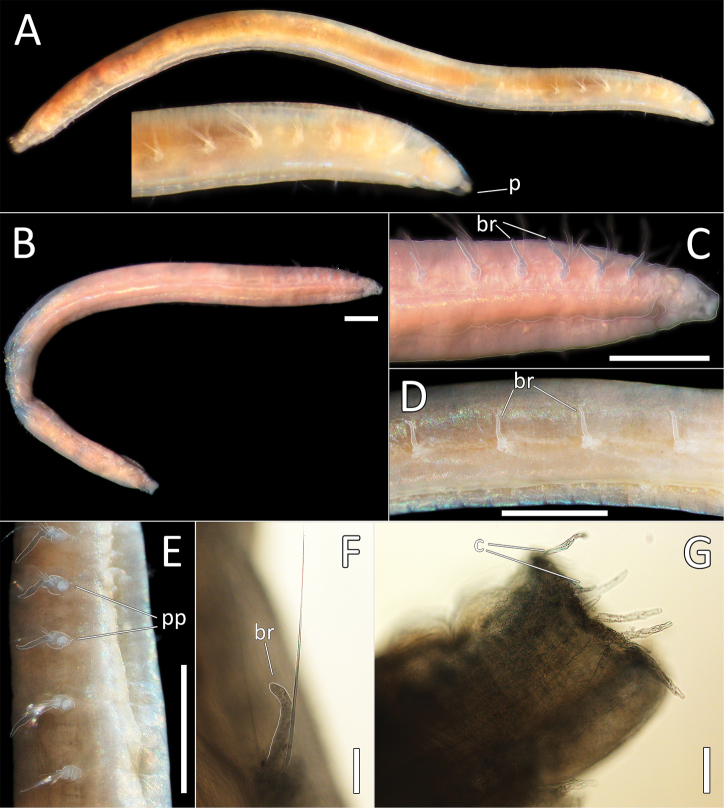
*Ophelina meyerae* sp. nov. holotype (specimen NHM_1241). **A** Live images, whole specimen with detail of anterior (p = palpode) **B** Lab image, whole specimen, lateral view (faded stain) **C** Lab image, anterior and branchiae (faded stain) (br = branchiae) **D** Lab image, mid-body branchiae (faded stain, br = branchiae) **E** Lab image, anterior parapodia (faded stain, pp = parapodia) **F** Lab image, detail of capillary chaetae (br = branchiae) **G** Lab image, detail of capillary chaetae (br = branchiae). Morphological features in plates **C–F** have been outlined with a fine white line to improve clarity of those features. Scale bars: 1 mm (**B–E**); 100 μm (**F, G**).

This is a medium-sized species represented by a single specimen. Body cylindrical, iridescent, some annulation detectable in first five and few posterior chaetigers, the rest of body smooth, no annulation detectable (Fig. 16A, B). Ventral groove distinct throughout the body. Live specimen semi-translucent, with orange gut (Fig. 16A). Complete specimen 20 mm long and 1.5 mm wide, with 29 chaetigers; anterior chaetigers not particularly crowded, degree of crowding observable in the posterior-most three to five chaetigers.

Prostomium of preserved specimen oval and broad (about as long as wide) and anteriorly bluntly rounded, somewhat truncated; bearing very distinct oval palpode (Fig. 16A). Nuchal organs observed as lightly pigmented slits laterally on posterior part of prostomium.

Branchiae present in all chaetigers, except for first chaetiger; branchiae remain attached in most chaetigers, including ch. 29, but are occasionally missing (lost) in some chaetigers. Branchiae easy to detect, although rather slender, best observed in anterior chaetigers (Fig. 16C), then getting progressively thinner and shorter and becoming more difficult to detect (Fig. 16D). All branchiae cirriform.

Parapodia distinct, biramous; with a broad lobe in chaetigers 2–10, becoming smaller in subsequent chaetigers; parapodia embedded in distinct lateral grooves (Fig. 16E). Chaetae are capillaries (Fig. 16F); not particularly numerous in any chaetigers, but most dense and longest in chaetigers 2–8, where of similar length, then becoming shorter, sometimes missing (broken off) entirely.

Anal tube well preserved; relatively short (about the length of two posterior chaetigers) and thick; distally symmetrical; distal opening with circlet of about 20 short, slender cirri with the exception of ventral part of the margin, which is smooth; ventral cirrus not observed (Fig. 16G).

##### Genetic data.

GenBank MN217457 for 16S and MN217506 for 18S. COI was unsuccessful for this specimen, no identical GenBank matches for 16S or 18S. In our phylogenetic analyses this species is sister to *Ophelina martinezarbizui* sp. nov. (Fig. 23).

##### Remarks.

Similar to *Ophelina martinezarbizui* sp. nov. in overall look and form of anal tube, but slender branchiae are present in all chaetigers, midbody and posterior branchiae are smaller than those of the anterior region.

##### Ecology.

Found in polymetallic nodule province of the eastern CCZ.

##### Etymology.

Named in honor of Kirstin Meyer-Kaiser, member of the science party onboard RV *Thomas G. Thompson* on the AB02 ABYSSLINE cruise in 2015.

#### Ophelina nunnallyi sp. nov.

Taxon classificationAnimaliaCapitellidaCapitellidae

60F9D08F-88AA-5BB6-801B-59948047605D

http://zoobank.org/3EE43467-76A5-4FFC-B19F-26C0063454CB

Fig. 17A–I

##### Material examined.

NHM_683 (**holotype**) NHMUK ANEA 2019.7133, coll. 20 Feb. 2015, 12°32.23N, 116°36.25W, 4425 m http://data.nhm.ac.uk/object/220fa671-4576-45b7-930d-efde148f223f; NHM_700 (**paratype**) NHMUK ANEA 2019.7134, coll. 20 Feb. 2015, 12°32.23N, 116°36.25W, 4425 m http://data.nhm.ac.uk/object/63115f48-bcf1-4b3b-9c2e-c339b97845bd; NHM_783F NHMUK ANEA 2019.7135, coll. 20 Feb. 2015, 12°32.23N, 116°36.25W, 4425 m http://data.nhm.ac.uk/object/376a42db-0497-4b4a-851b-c1d5e07bd2b6; NHM_1273 (**paratype**) NHMUK ANEA 2019.7136, coll. 01 Mar. 2015, 12°15.44N, 117°18.13W, 4302 m http://data.nhm.ac.uk/object/a3540563-8a0c-475b-96b5-12969fb8c2ba; NHM_1309A (SEM specimen) NHMUK ANEA 2019.7137, coll. 01 Mar. 2015, 12°15.44N, 117°18.13W, 4302 m http://data.nhm.ac.uk/object/25066e63-ecc9-439a-9907-eaeaeb72e78c.

##### Type locality.

Pacific Ocean, CCZ, 12°32.23N, 116°36.25W, depth 4425 m, in mud between polymetallic nodules.

##### Description.

This species is represented by five complete specimens, none in an excellent condition, anal tube damaged and not clearly observed. However, images of live specimens are available to observe some now missing or damaged features such as anal tube.

**Figure 17.**
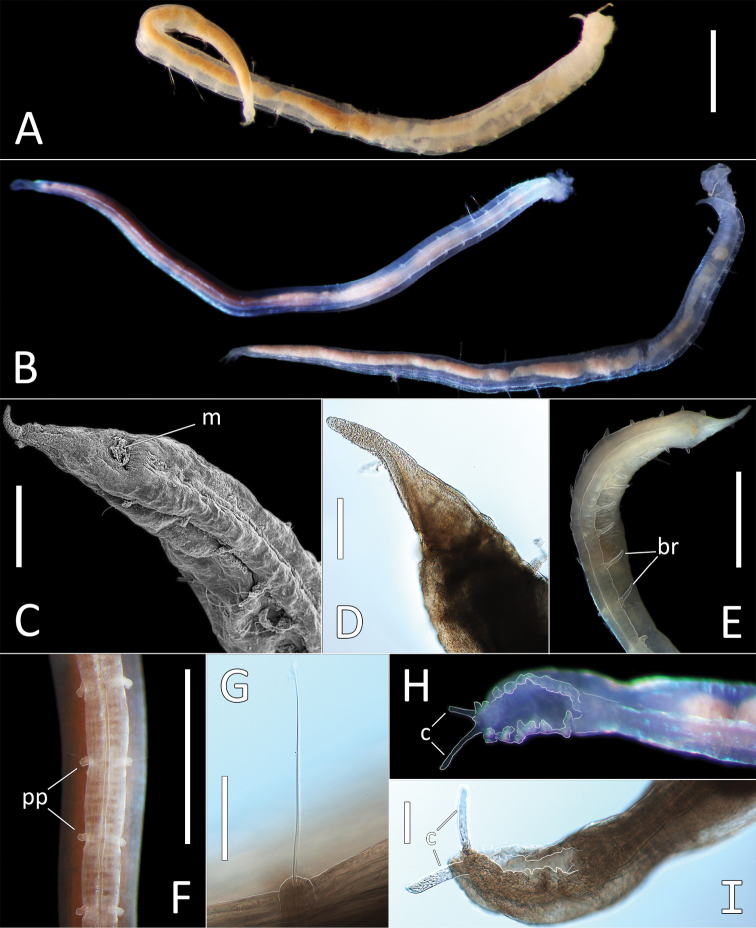
*Ophelina nunnallyi* sp. nov. **A** Lab image, whole specimen (paratype NHM_700) **B** Live images, whole specimens (holotype [specimen NHM_683] [left], paratype NHM_700 [right]) **C** SEM image, anterior and palpode (specimen NHM_1309A, m = mouth) **D** Lab image, detail of palpode, (paratype NHM_1273) **E** Lab image, anterior and branchiae (paratype NHM_1273, br = branchiae) **F** Lab image, parapodia (holotype, pp = parapodia) **G** Lab image, capillary chaeta (holotype) **H** Live image, anal funnel (holotype, c = cirri) **I** Lab image, detail of anal funnel (holotype, c = cirri). Morphological features in plates **E, F, H, I** have been outlined with a fine white line to improve clarity of those features. Scale bars: 1 mm (**A, E, F**); 200 μm (**C, D**); 100 μm (**G, I**).

Small to medium-sized species 9–14 mm long and 0.25–0.8 mm wide, for 33 chaetigers. Body cylindrical and smooth without distinct annulation. Preserved specimen yellow semi-translucent, iridescent in ethanol (Fig. 17A); live specimens translucent (Fig. 17B). Ventral and lateral grooves distinct along whole length of the body. Chaetigers elongated, only slightly crowded in first eight anterior chaetigers and towards posterior end.

Prostomium of preserved specimen conical (longer than wide), anteriorly pointed and extending into very long, thick palpode (Fig. 17C, D). Rounded, slightly pigmented nuchal organs everted in specimen NHM_683.

Branchiae observed in anterior chaetigers only, some missing (broken off), present from chaetigers 4–9 (Fig. 17E). All branchiae cirriform, conspicuous but relatively short, straight, distally blunt.

Parapodia biramous, embedded in lateral grooves; observed as distinct conical lobes throughout the body (Fig. 17F). Chaetae are capillaries only (Fig. 17G); often missing (broken off), most abundant in anterior eight chaetigers.

Anal tube attached (in holotype, NHM_783F and NHM_1309A), but now poorly preserved and variably damaged, always separated from the rest of the body by a shallow constriction (Fig. 17H, I). Anal tube of live specimen well-imaged in Fig. 17H, where anal tube scoop-shaped, ventrally with wide depression; distally with two elongate, slender cirri; no other cirri observed, but may be missing.

##### Additional morphological observations.

In addition to the material examined here, several more specimens consistent with this species have been found, but no DNA sequencing has been carried out on these and thus they are not included in this manuscript. Where the anal tube was observed, it is scoop-shaped, but the preservation of cirri is variable. In some specimens, short slender cirri can be detected on the lateral margins of the anal tube. Chaetiger counts consistent with 33 chaetigers. Branchiae were consistently observed on chaetigers 4–9. However, in the absence of DNA data we are reluctant to ascribe these specimens formally to *O. nunnallyi* sp. nov. until further analyses has been done.

##### Genetic data.

GenBank MN217458-MN217462 for 16S, MN217507 for 18S and MN217532–MN217534 for COI. This species is genetically identical to or very similar to “*Ophelina* sp. 1” (Janssen et al. 2015), with K2P values ranging from 0.002–0.006 between *O. nunnallyi* sp. nov. and the already published sequences with accession numbers KJ736582–KJ736588. *Ophelina nunnallyi* sp. nov. is sister to *Ophelina* sp. (NHM_1068) in our phylogenetic analyses (Fig. 23).

##### Remarks.

Other than sp. NHM_1068 (see Remarks under sp. NHM_1068), the DNA suggest similarity of *Ophelina nunnallyi* sp. nov. to *O. acuminata*, originally described from the shallow coast of Denmark, but frequently reported in all oceans (see references in Parapar et al. 2011). These frequent records likely constitute an error as similar, but unrecognized species were likely confused. Deep-sea records of *Ophelina aulogaster* (Rathke, 1843) by for example Hartman (1965), and Fauchald (1972, may refer to a similar, but unrecognized, species. Hartman (1965) recognized deep Atlantic specimens as a distinct species and later erected *Ophelina aulogastrella* (Hartman & Fauchald, 1971), which lacks branchiae in posterior region, with most of them present in chaetigers 4–10 (or 13) and anal tube is scoop-shaped with some (easily lost) cirri. Such morphology agrees well with UKSR specimens. However, Hartman (1965) reported variable number of segments (28–36) and wide bathyal distribution (196–5023 m) for *O. aulogastrella*, which may suggest that more than one species was in fact present in Hartman’s material. The type locality of *O. aulogastrella* is North Atlantic.

The first occurrence of branchiae from chaetiger 4 is very unusual in *Ophelina*, where branchiae appear from chaetiger 2. Branchiae are fragile and easily lost structures; therefore, we cannot exclude a possibility that branchiae prior to chaetiger 4 are present but lost in our specimens. Nevertheless, this distribution has been observed in all material examined (including additional specimens, no DNA available) as well as in very similar species *Ophelina* sp. (NHM_1068).

##### Ecology.

Found in polymetallic nodule province of the eastern CCZ.

##### Etymology.

Named in honor of Clifton Nunnally, member of the science party of both the ABYSSLINE cruises.

#### Ophelina cf. abranchiata

Taxon classificationAnimaliaCapitellidaCapitellidae

Støp-Bowitz, 1948

C6FAD490-14BD-5775-8C17-48EBF00DB765

##### General comments on Ophelina abranchiata and similar morphotypes.

Three small, abranchiate morphospecies found in the UKSR material, *Ophelina ganae* sp. nov., O. cf. abranchiata NHM_1769 and O. cf. abranchiata NHM_2017, are very similar to *Ophelina abranchiata* Støp-Bowitz, 1948. This species has its type locality as East Greenland, 200 m depth, but has subsequently been reported worldwide, from predominantly deep waters [see references in Kongsrud et al. (2011), Parapar et al. (2011)]. Sene-Silva (2007) proposed *Polyophthalmus translucens* Hartman, 1960 and *Ophelina farallonensis* Blake, 2000, both described from deep waters off California, as junior synonyms of *O. abranchiata*. Blake (2000b) had separated *O. farallonensis* from *O. abranchiata* due to presence of long anal tube, but he most likely did not realize that the original description of *O. abranchiata* by Støp-Bowitz (1948) was based on an incomplete specimen without an anal tube as already suggested by Sene-Silva (2007), Kongsrud et al. (2011) and Parapar et al. (2011). The absence of branchiae also likely led to confusion of *O. abranchiata* with the much larger (30 mm long, 30 chaetigers) abranchiate species *Ophelina nematoides* by some workers (e.g. Maciolek and Blake 2006).

Such confused taxonomic history is further complicated by the fact that published (Neal et al. 2018) and unpublished (Kongsrud pers. comm.) molecular data suggest presence of several species within an *O. abranchiata* complex. Kongsrud et al. (2011) provided a re-description of type material, together with report on material from North Sea and North Atlantic, but without providing genetic data. One possible, and previously overlooked, character may be the number of chaetigers as Kongsrud et al. (2011) reported an invariable number of 18 chaetigers for *O. abranchiata*, and considered specimens bearing17 chaetigers from Skagerrak in need of further evaluation, not ascribing these to *O. abranchiata*. In other reports, variation of 17–19 chaetigers (4–10 mm long) was given by Barroso and Paiva (2013) and Parapar and Moreira (2008) referred to 16–22 segments with detectable annulation, rather than chaetigers. Hartman (1960) reported 18 chaetigers for *P. translucens* and Blake (2000b) reported 18 or 19 chaetigers (5–7 mm long) for *O. farallonensis*.

The anal tube, an important feature upon which opheliid species have been differentiated in the past is mostly missing in these morphotypes even where hundreds of specimens are available (Neal pers. obs.). Where the anal tube has been observed (Blake 2000b; Parapar and Moreira 2008; Kongsrud et al. (2011); Parapar et al. 2011; Barroso and Paiva 2013; Neal pers. obs.) its form appears to be very similar.

Parapar and Moreira (2008) provided the first SEM examination of *O. abranchiata* morphotypes and reported presence of lateral organs between notopodia and neuropodia for Iberian specimens. Subsequently, examination of Icelandic morphotypes by Parapar et al. (2011) led to the report of presence of lateral organs in all chaetigerous segments as well as in the anterior segments of the body which are achaetigerous and devoid of parapodia, suggesting the presence of three such segments. However, this feature was not observed in SEM examination of bathyal material from Brazil by Barroso and Paiva (2013). Despite this variation, none of these authors suggested lateral organs as a useful taxonomic character and previously Purschke and Hausen (2007) only considered that they might be useful for higher level systematics of annelids. Although the presence and distribution of lateral organs was only investigated in non-type specimens (Parapar and Moreira 2008; Parapar et al. 2011; Barroso and Paiva 2013), their presence reported is some specimens and absence in others could possibly be result of interspecific variability. However, it is important to stress that these structures are extremely small (around 5 µm) and their observation depends on the quality of the specimens (i.e. in shriveled specimens, this feature may be impossible to observe). Two UKSR specimens were investigated under SEM for lateral organs. Lateral organs were confirmed in both specimens examined. Three lateral organs were associated with three prechaetigerous segments and then one observed between the noto- and neuropodium of each chaetiger. Such distribution is consistent with previous observations (Parapar and Moreira 2008; Parapar et al. 2011). The pattern which pore openings form in each morphotype appears to vary, but currently we cannot confirm consistency of such observation due to the low number of specimens examined so far.

Clearly, additional morphological characters are needed to distinguish small abranchiate species currently lumped under *O. abranchiata*, *P. translucens* and *O. farallonensis*.

#### Ophelina cf. abranchiata

Taxon classificationAnimaliaCapitellidaCapitellidae

(NHM_1769)

9FD40530-9F3F-580F-9098-10C03BF72F22

Fig. 18A–E

##### Material examined.

NHM_1769 NHMUK ANEA 2019.7148, coll. 11 Mar. 2015, 12°10.43N, 117°11.57W, 4045 m http://data.nhm.ac.uk/object/8a2cbe4f-277d-4355-a34f-0b53c797bef0.

##### Description.

This species is represented by a single specimen, in reasonable condition, but anal tube is missing. Small species, 5.3 mm long and 0.3 mm wide; the exact number of chaetigers is difficult to establish, but at least 16 counted, although 17 may be present.

**Figure 18.**
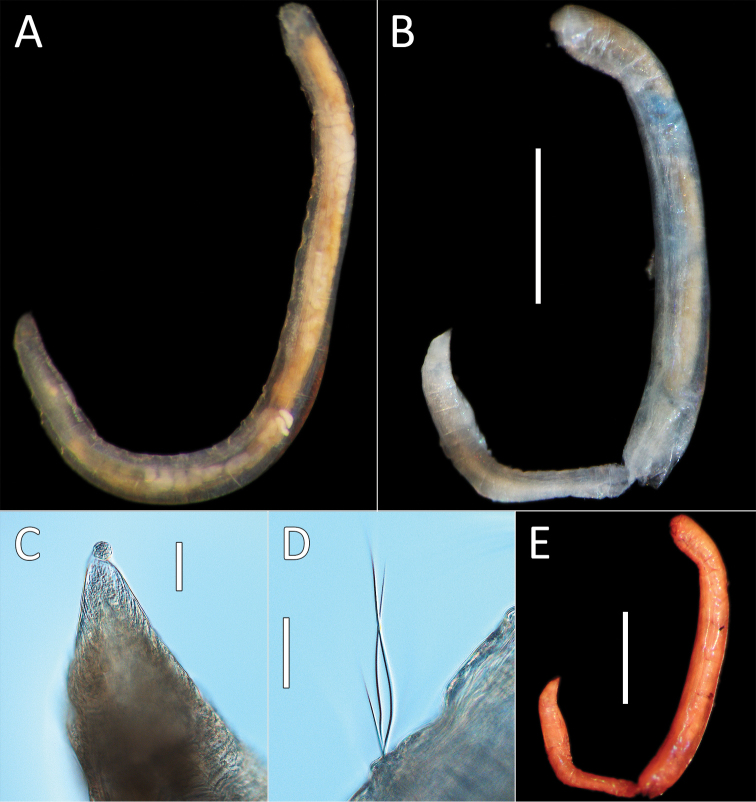
Ophelina cf. abranchiata sp. NHM_1769 (specimen NHM_1769). **A** Live image, whole specimen **B** Lab image, whole specimen (very faded post-stain,) **C** Lab image, detail of prostomium and palpode **D** Lab image, detail of chaetae **E** Lab image, whole specimen (stained). Scale bars: 1 mm (**B, E**); 50 μm (**C, D**).

Body cylindrical and smooth without distinct annulation (Fig. 18A, B). Preserved specimen yellow in ethanol; live specimen translucent, with yellow gut (Fig. 18A). Ventral and lateral grooves distinct along whole length of body. Anterior and posterior-most chaetigers slightly shorter than mid-body chaetigers.

Prostomium elongate, conical with small acute terminal palpode (Fig. 18C). Eyes not observed. Nuchal organs rounded, laterally on posterior part of prostomium. Peristomium indistinct. Anterior prechaetigerous region elongated, number of achaetous segments unknown (no SEM). Parapodia rudimentary, biramous, embedded in lateral grooves; no distinct pre- or postchaetal lobes.

Chaetae all slender, smooth capillaries (Fig. 18D), appear in small numbers in both rami, often broken off entirely. Notochaetae mostly longer than neurochaetae.

Branchiae absent. Anal tube not observed. Shirlastained specimens without distinct pattern (Fig. 18E).

##### Genetic data.

GenBank MN217433 for 16S, MN217501 for 18S and MN217520 for COI. In our phylogenetic analyses it is part of a well-supported clade with *Ophelina ganae* sp. nov., Ophelina cf. abranchiata (NHM_2017) and at least two other abranchiate opheliids (Fig. 23).

##### Remarks.

Please refer to section “General comments on *Ophelina abranchiata* and similar morphotypes” above and remarks for *Ophelina ganae* sp. nov.

#### Ophelina cf. abranchiata

Taxon classificationAnimaliaCapitellidaCapitellidae

sp. (NHM_2017)

17DD004E-DC3B-5850-8EDB-19FFE7ED573F

Fig. 19A–F

##### Material examined.

**NHM_2017** NHMUK ANEA 2019.7149, coll. 16 Mar. 2015, 12°03.03N, 117°24.28W, 4235 m http://data.nhm.ac.uk/object/9ebcd947-c53b-4616-81d4-da42afaeca03.

##### Description.

This species is represented by a single specimen, in reasonable condition, but anal tube is missing. Small species, 4 mm long and 0.35 mm wide; exact number of chaetigers difficult to establish, but at least 17 counted, although 18 may be present.

**Figure 19.**
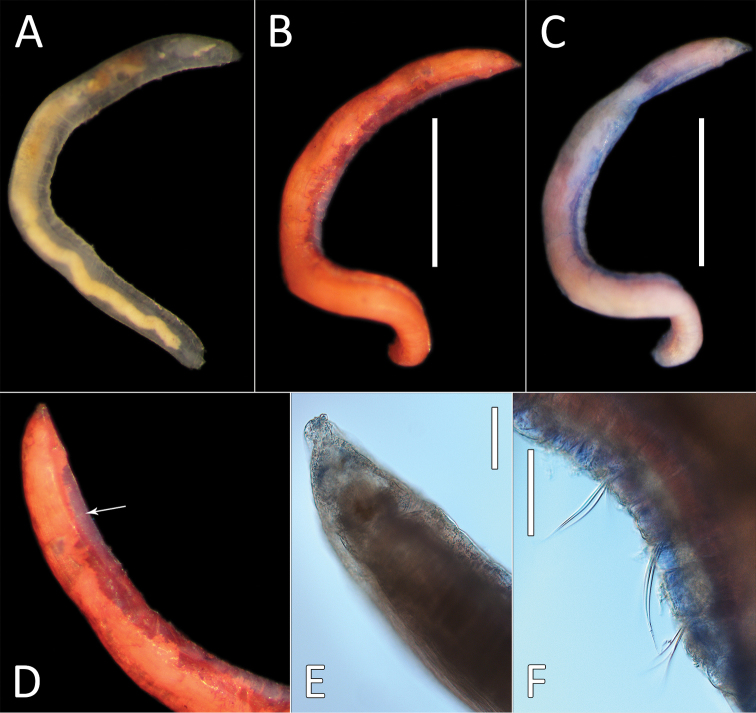
Ophelina cf. abranchiata sp. NHM_2017 (specimen NHM_2017). **A** Live image, whole specimen **B** Lab image, whole specimen (stained,) **C** Lab image, whole specimen (faded stain) **D** Lab image, anterior (stained, arrow highlighting dark banding) **E** Lab image, detail of anterior **F** Lab image, detail of capillary chaetae. Scale bars: 1 mm (**B, C**); 100 μm (**E, F**).

Body cylindrical and smooth without distinct annulation (Fig. 19A–C). Preserved specimen yellow in ethanol; live specimen translucent, with yellow gut (Fig. 19A). Ventral and lateral grooves distinct along whole length of body. Anterior and posterior-most chaetigers slightly shorter than mid-body chaetigers.

Prostomium elongate, conical with small acute terminal palpode (Fig. 19D, E). Eyes not observed. Nuchal organs rounded, laterally on posterior part of prostomium. Peristomium indistinct. Anterior prechaetigerous region elongated, number of achaetous segments unknown (no SEM). Parapodia rudimentary, biramous, embedded in lateral grooves; no distinct pre- or postschaetal lobes.

Chaetae all slender, smooth capillaries (Fig. 19F), appear in small numbers in both rami, often broken off entirely. Notochaetae mostly longer than neurochaetae.

Branchiae absent. Anal tube not observed. Shirlastained specimens with wide, dark red, strongly stained stripe on the dorsum (Fig. 19B, D), not observed in species *Ophelina ganae* sp. nov. and Ophelina cf. abranchiata (NHM_1769).

##### Genetic data.

GenBank MN217434 for 16S. In our phylogenetic analyses, Ophelina cf. abranchiata sp. (NHM_2017) is part of a well-supported clade including *Ophelina ganae* sp. nov., Ophelina cf. abranchiata (NHM_1769) and at least two other abranchiate opheliid species (Fig. 23).

##### Remarks.

Please refer to section “General comments on O*phelina abranchiata* and similar morphotypes” above and remarks for *Ophelina ganae* sp. nov.

#### Ophelina

Taxon classificationAnimaliaCapitellidaCapitellidae

sp. (NHM_689)

183CA00D-492C-5331-A06D-7C4DCF0034B4

Fig. 20A–H

##### Material examined.

NHM_689 NHMUK ANEA 2019.7114, coll. 20 Feb. 2015, 12°32.23N, 116°36.25W, 4425 m http://data.nhm.ac.uk/object/6755d584-a20a-4ce5-a4f1-32ce0965128e.

##### Description.

**Figure 20.**
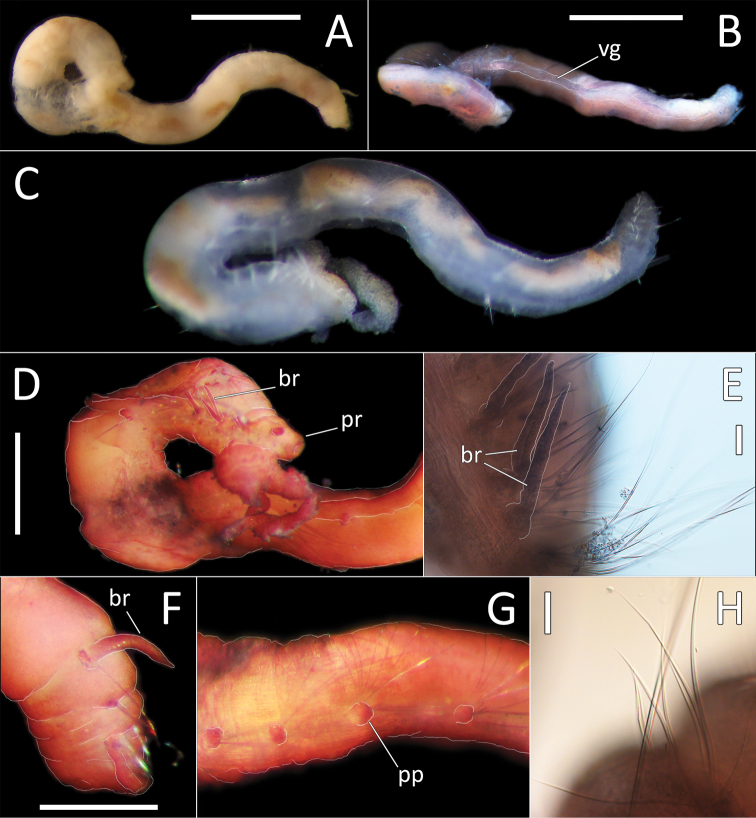
*Ophelina* sp. NHM_689 (specimen NHM_689). **A** Lab image, whole specimen, lateral view (pre-stain) **B** Lab image, whole specimen, ventral view (faded stain, vg = ventral groove) **C** Live image, whole specimen **D** Lab image, damaged anterior (stained, br = branchiae, pr = prostomium) **E** Lab image, detail of anterior branchiae (br = branchiae) **F** Lab image, posterior branchiae (stained, br = branchiae) **G** Lab image, mid-body parapodia (stained, pp = parapodia) **H** Lab image, detail of capillary chaetae. Morphological features in plates **B, D–G** have been outlined with a fine white line to improve clarity of those features. Scale bars: 1 mm (**A, B**); 0.5 mm (**D**); 50 μm (**E**); 0.25 (**F, H**).

This species is represented by a single specimen in poor condition, with anal tube and most of branchiae missing. Posteriorly incomplete specimen 4.7 mm long and 0.35 mm wide for at least 22 chaetigers (exact number of chaetigers is difficult to count in places). Body cylindrical and smooth without distinct annulation (Fig. 20A–C). Ventral and lateral grooves distinct throughout body. Preserved specimen yellow in ethanol (Fig. 20A); live specimen translucent, pale brown gut (Fig. 20C). Chaetigers crowded in anterior part of the body and posterior part of the body (the last four chaetigers), elongated in the mid-section of the body.

Prostomium of preserved specimen conical, broad (only slightly longer than wide), anteriorly bluntly rounded (but prostomium appears damaged) (Fig. 20D). Proboscis extended, damaged, soft inflated sack-like structure observed.

Branchiae present, but many are likely missing. Branchiae observed in chaetigers 2–4 (Fig. 20D, E) and then in posterior region, where only one branchia remains attached on the fourth before the last chaetiger (Fig. 20F); no branchiae observed in mid-body region.

Parapodia distinct, biramous; embedded in lateral grooves (Fig. 20G). Chaetae are capillaries only (Fig. 20H); all very long but longest on chaetiger 1 where they are nearly twice the length of chaetae of subsequent chaetigers. Anal tube likely missing (damaged).

##### Genetic data.

GenBank MN217463 for 16S and MN217508 for 18S. COI was unsuccessful for this specimen, no identical GenBank matches for 16S or 18S. In our phylogenetic tree, *Ophelina* sp. (NHM_689) is sister to *Ophelina cylindricaudata* (Hansen, 1879) from the Atlantic (New England) (Fig. 23).

##### Remarks.

Due to the condition of the single specimen representing this morphospecies, important diagnostic characters such as the structure of the anal tube and distribution of the branchiae cannot be determined. See Remarks under *Ophelina* sp. (NHM_1331) for more details.

#### Ophelina

Taxon classificationAnimaliaCapitellidaCapitellidae

sp. (NHM_1068)

6AEBB923-87EF-5FD2-B4C3-85E725A50CDE

Fig. 21A–H

##### Material examined.

NHM_1068 NHMUK ANEA 2019.7138, coll. 26 Feb. 2015, 12°06.93N, 117°09.87W, 4100 m http://data.nhm.ac.uk/object/b28fd52f-5717-45e3-b0cc-369172a690e5; NHM_1874 NHMUK ANEA 2019.7139, coll. 13 Mar. 2015, 12°02.49N, 117°13.03W, 4094 m, http://data.nhm.ac.uk/object/c3ffe5f4-6ca3-4816-966c-25ec98bbb003.

##### Description.

**Figure 21.**
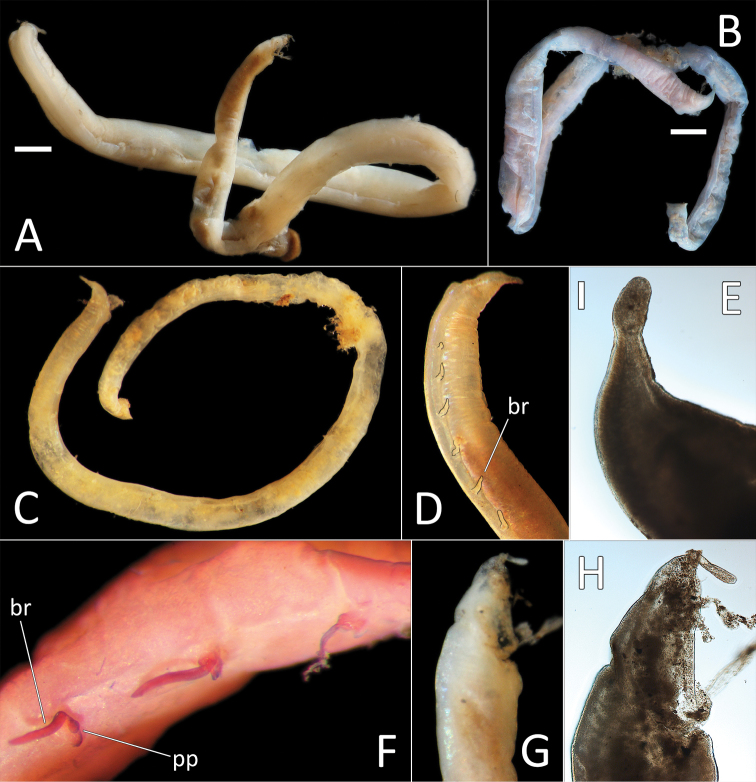
*Ophelina* sp. NHM_1068. **A** Lab image, whole specimen (specimen NHM_1068,) **B** Lab image, whole specimen (specimen NHM_1874, faded stain) **C** Live image, whole specimen (specimen NHM_1874) **D** Live image, anterior, with branchiae outlined in a fine black line (specimen NHM_1068, br = branchiae) **E** Lab image, detail of palpode (specimen NHM_1068) **F** Lab image, detail of anterior parapodia and branchiae (specimen NHM_1874, stained) **G** Lab image, posterior (specimen NHM_1068) **H** Lab image, detail of anal funnel (specimen NHM_1068). Scale bars: 1 mm (**A, B**); 100 μm (**E**).

This species is represented by two specimens, both in poor condition; specimen NHM_1874 posteriorly incomplete, specimen NHM_1068 mostly complete, but anal tube damaged. Large species 25–30 mm long and 0.8 mm wide, for minimum of 30 chaetigers (exact number of chaetigers cannot be established). Body cylindrical and smooth without distinct annulation (Fig. 21A–C). Preserved specimen yellow in ethanol (Fig. 21A); live specimens translucent, yellowish (Fig. 21C, D). Ventral and lateral grooves distinct along whole length of the available fragments. Chaetigers somewhat crowded in anterior part of the body, then elongated in the rest of the body, posterior-most chaetigers not observed.

Prostomium of preserved specimen conical (longer than wide), anteriorly pointed and extending into very large and long thick palpode (Fig. 21D, E). Branchiae observed in anterior chaetigers only, but many missing (broken off) and the exact distribution cannot be confirmed; mainly observed in chaetigers 4–13; branchiae conspicuous but rather short, straight, distally blunt (Fig. 21D, F).

Parapodia biramous, embedded in lateral grooves; parapodia small conical lobes, no distinct pre- or postchaetal lobes observed (Fig. 21F). Chaetae are capillaries only; often missing (broken off).

Anal tube missing in specimen NHM_1874; damaged in NHM_1068, but probably scooped-shaped (Fig. 21G-H).

##### Genetic data.

GenBank MN217464 and MN217466 for 16S and MN217509 for 18S. COI was unsuccessful for this specimen, no identical GenBank matches for 16S and 18S.

##### Remarks.

According to our molecular results, this species forms a clade with *Ophelina nunnallyi* sp. nov., which is sister to *O. acuminata* (Fig. 23), but due to specimen damage, meaningful morphological comparison cannot be currently provided. *Ophelina* sp. (NHM_1068) and *O. nunnallyi* sp. nov. share a similar prostomium shape and its associated robust palpode, branchiae occurring from chaetiger 4 that are limited to anterior part of the body (ch. 4–9). However, *Ophelina* sp. (NHM_1068) has a larger body size. See also Remarks under *Ophelina nunnallyi* sp. nov. above.

#### Ophelina

Taxon classificationAnimaliaCapitellidaCapitellidae

sp. (NHM_1331)

10F31301-A643-54F7-AED3-E67659321BCC

Fig. 22A–G

##### Material examined.

NHM_1331 NHMUK ANEA 2019.7115, coll. 01 Mar. 2015, 12°15.44N, 117°18.13W, 4302 m http://data.nhm.ac.uk/object/06d48d7f-7339-4cc5-8445-b51a980e4e0f.

##### Description.

**Figure 22.**
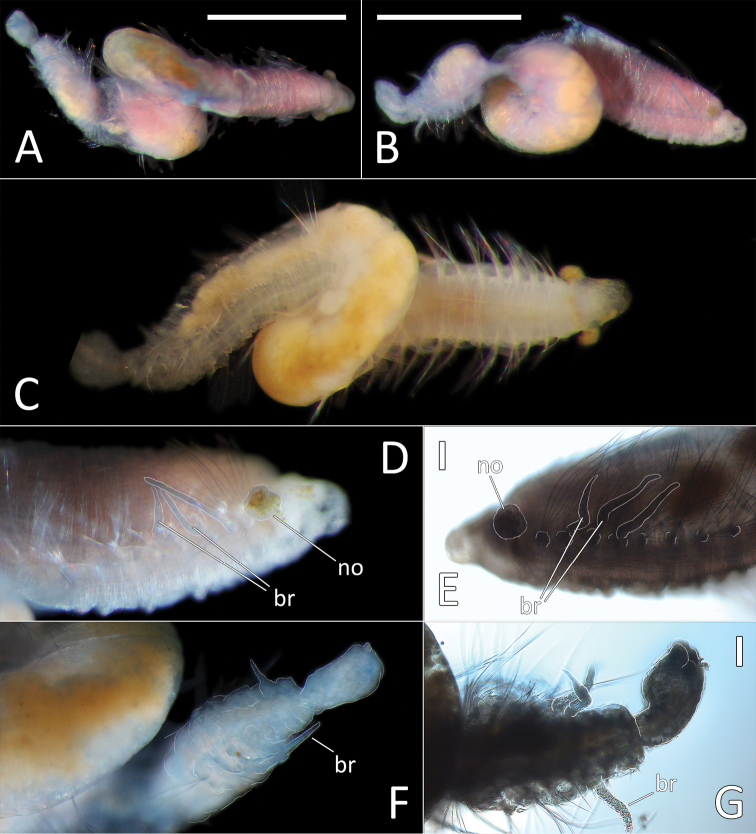
*Ophelina* sp. NHM_1331 (specimen NHM_1331). **A** Lab image, whole specimen, dorsal view (faded stain) **B** Lab image, whole specimen, lateral view (faded stain) **C** Live image, whole specimen **D** Lab image, anterior (faded stain, br = branchiae, no = nuchal organ) **E** Lab image, detail of anterior (br = branchiae, no = nuchal organ) **F** Lab image, posterior, anal funnel (br = branchiae) **G** Lab image, detail of posterior and anal funnel (br = branchiae). Morphological features in plates **B, D–G** have been outlined with a fine white line to improve clarity of those features. Scale bars: 1 mm(**A, B**); 100 μm (**E, G**).

This species is represented by a single complete specimen in relatively good condition. Specimen about 4.5 mm long and 0.5 mm wide for about 28 chaetigers. Body cylindrical and smooth with some annulation detectable (Fig. 22A–C). Ventral and lateral grooves distinct throughout body. Preserved specimen yellow in ethanol; live specimen translucent, with yellow gut (Fig. 22C); everted nuchal organs with golden brown pigment (Fig. 22A–D). Chaetigers crowded in anterior part of the body and posterior part of the body (the last four chaetigers), elongated in the mid-section of the body.

Prostomium of preserved specimen conical, broad (only slightly longer than wide) and anteriorly bluntly rounded; palpode not observed (Fig. 22D, E). Nuchal organs laterally on posterior part of prostomium, everted, round with golden brown pigment observable even in preserved specimen.

Branchiae present; with disjointed distribution, with three pairs on chaetigers 2–4 (Fig. 22D, E) and three pairs in posterior region on chaetigers 21–24 (Fig. 22F, G); branchiae in other chaetigers not observed, branchiae considered absent in the last four crowded chaetigers. All branchiae cirriform, of similar length.

Parapodia distinct, biramous; embedded in lateral grooves on chaetigers 1–24; no distinct pre- or postchaetal lobes. Chaetae are capillaries only; all very long but longest on chaetiger 1 where they are nearly twice the length of chaetae of subsequent chaetigers.

Anal tube attached, but not well preserved; cylindrical; appears distally asymmetrical with dorsal lobe overlapping the ventral lobe (but this may be an artefact of poor preservation) (Fig. 22F, G); cirrus not observed.

##### Genetic data.

**Figure 23.**
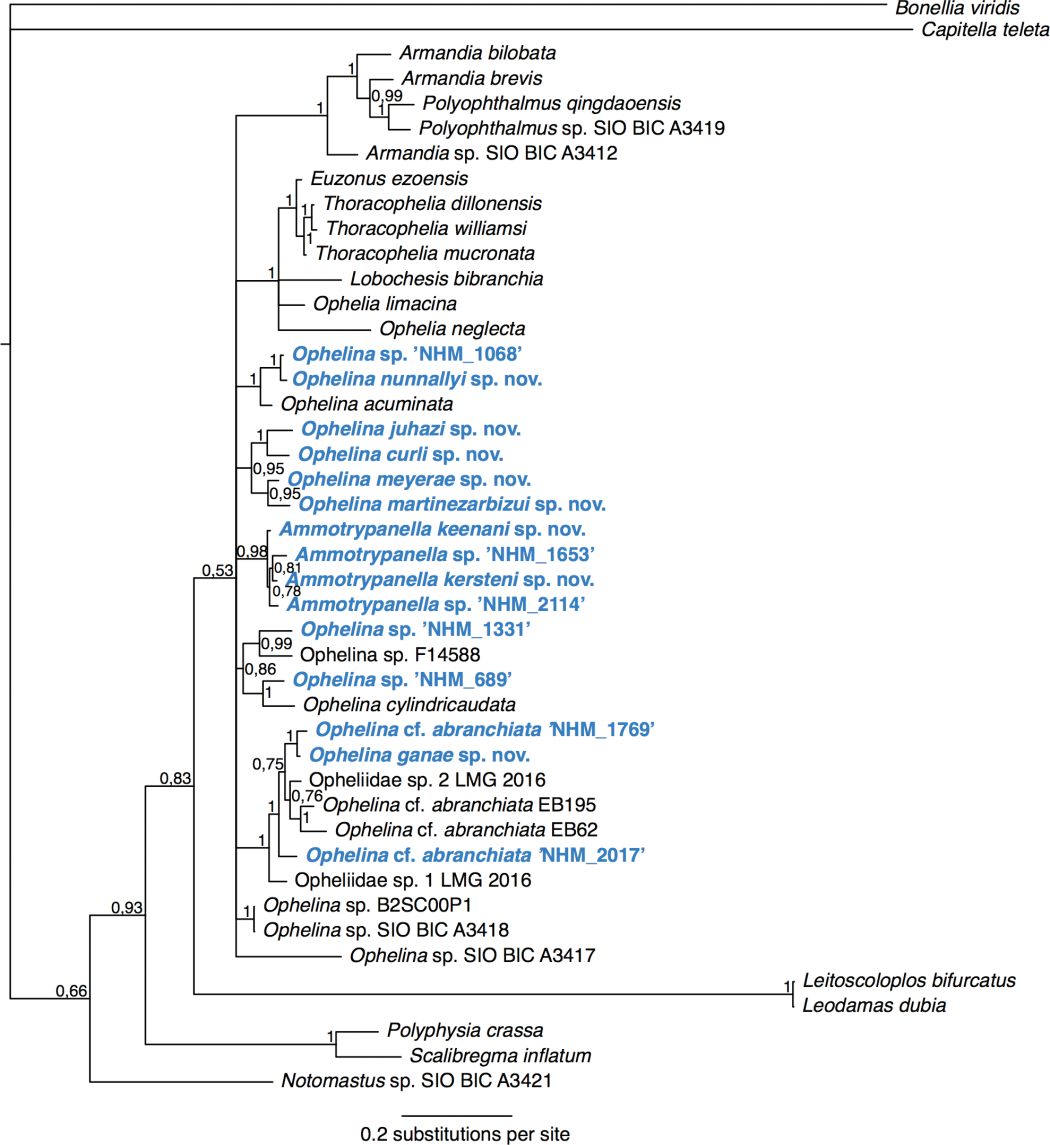
Phylogenetic analysis of Opheliidae. 50% majority rule tree from the Bayesian analyses using 18S and 16S, with posterior probability values on nodes. Twenty-nine taxa from GenBank were included, and Capitellidae and Echiura was chosen as outgroup following the annelid phylogeny of Weigert and Bleidorn (2016).

GenBank MN217465 for 16S and MN217510 for 18S. COI was unsuccessful for this specimen, no identical GenBank matches for 16S or 18S. In our phylogenetic tree, *Ophelina* sp. (NHM_1331) is sister to “*Ophelina* sp. F14588” forming a clade with the taxa *Ophelina cylindricaudata* from the Atlantic (New England) and *Ophelina* sp. (NHM_689) (Fig. 23).

##### Remarks.

Morphologically, *Ophelina* sp. (NHM_1331) is similar to *Ophelina* sp. (NHM_689) in having a broad prostomium and very long chaetae on chaetiger 1. Their branchiae appear to be arranged in a similar pattern (three pairs are present in chaetigers 2–5 and then few pairs present in posterior chaetigers). Nuchal organs are not everted in *O.* sp. (NHM_689). They may differ in number of chaetigers, although this is difficult to establish due to damage of *O.* sp. (NHM_689), anal tube has not been observed in *O.* sp. (NHM_689) (assumed missing) and cannot be compared.

Of known species of *Ophelina*, *O.* sp. (NHM_1331) is similar to a group with 28 chaetigers and four posterior parapodia crowded: *O. cylindricaudata*, *O. breviata* (Ehlers, 1913) and *O. brattegardi*Kongsrud et al., 2011.

The lack of branchiae in midbody has been described in some of these species, but for *O. cylindricaudata* this has been clarified as a mistake in the original description. Kongsrud et al. (2011) reported that branchiae in the mid-body region may be present in *O. cylindricaudata* but are often reduced in size and when they are lacking there is no consistent pattern. The absence of branchiae is considered “true” in *Ophelina chaetifera* (Hartman 1965), described from the deep Atlantic (1330–5007 m) and *O. brattegardi* described from Iceland Sea (off East Greenland) in 1600 m depth. The UKSR species differs from both in having broad, anteriorly blunt prostomium. *Ophelina chaetifera* further differs in having only 26 chaetigers. However, with only single specimen available for examination, we cannot establish “true” branchial distribution in UKSR samples.

#### Scalibregmatidae

Taxon classificationAnimaliaCapitellidaCapitellidae

Malmgren, 1867

E0D17068-E072-50D9-AE40-4864940CCC19

##### Notes.

The family Scalibregmatidae was established by Malmgren (1867) to accommodate annelids with rugose appearance of the cuticle and either elongate (arenicoliform) or maggot-shaped body form, with often anteriorly inflated bodies such as *Scalibregma inflatum* Rathke, 1843 and *Polyphysia crassa* (Ørsted, 1843). Currently, there are over 50 valid scalibregmatid species (Read and Fauchald 2018a) but see also discussion about *Travisia*.

The characters used to differentiate genera are the prostomial shape, presence and development of branchiae, presence of spines in anterior notopodia (and sometimes also in neuropodia), presence and development of branchiae and development of dorsal and ventral cirri, particularly in posterior part of the body (e.g. Blake 1981, 2000c). However, these characters are considered problematic due to their overlap between genera (Blake 2000c). Additionally, characters such as the form of the prostomium and presence of branchiae depend on the stage of ontogeny (Blake 2015). Recently, Blake (2015) reported previously overlooked characters that he considers species specific such as development of ventral part of the peristomium into complex upper and lower lips surrounding the mouth; form of short, spinous chaetae anterior to capillaries in parapodia preceding lyrate chaetae and development of internal glands within parapodia. Blake (2015) also concluded that small specimens cannot be reliably referred to a species or genus without a growth sequence and previously described species should be re-examined for characters such as presence of spinous chaetae in anterior parapodia and development of internal glands.

Although Scalibregmatidae range from the intertidal to the deep sea, most species occur below 1000 m (Blake 2015). Four scalibregmatid species were encountered in the UKSR-collected material. Three of these are assigned to the genus *Oligobregma* Kudenov & Blake, 1978, while one species could not be assigned to a genus based on morphology due to its poor condition. Generic assignment of ABYSSLINE species to *Oligobregma* is based on the presence of the following characters: elongate arenicoliform body, prostomium with prominent frontal horns, absence of branchiae, presence of spines in anterior chaetigers and presence of well-developed dorsal and ventral cirri in posterior chaetigers.

The diagnosis of *Oligobregma* presented here is amended from that given by Blake (2017), mainly to take into account a more posterior appearance of furcate chaetae, which Blake (2017) considered to appear prior to chaetigers 2–4.

##### Diagnosis.

Body elongate and arenicoliform. Prostomium T-shaped with two prominent frontal horns. Eyes present or absent, nuchal organs present. Peristomium achaetous, surrounding prostomium dorsally and forming upper and lower lips of mouth ventrally. Branchiae absent. Parapodia well developed, with dorsal and ventral cirri on posterior chaetigers; interramal papillae present or absent. Large acicular spines present on anterior chaetigers. Capillaries present in all parapodia; lyrate chaeta present. Some species with short, slender, blunt or pointed spinous chaetae anterior to capillaries of chaetigers 1, 2 or 3, representing homologues of lyrate chaetae. Pygidium with anal cirri.

#### Oligobregma brasierae sp. nov.

Taxon classificationAnimaliaCapitellidaCapitellidae

553830CF-ABB9-572E-93EC-A8BA7DB544E5

http://zoobank.org/2FC2E16E-1463-4D6A-B3C1-90FEDFB222BC

Figs 24A–J , 25A–C

##### Material examined.

NHM_032 NHMUK ANEA 2019.7150, coll. 09 Oct. 2013, 13°50.232N, 116°33.506W, 4336 m http://data.nhm.ac.uk/object/43545746-b8ad-43a8-92b7-53637dd131d6; NHM_404 NHMUK ANEA 2019.7151, coll. 20 Oct. 2013, 13°51.797N, 116°32.931W, 4050 m http://data.nhm.ac.uk/object/5fda0cac-0a77-4ec7-a2fa-5cd529548a19; NHM_684 (**paratype**) NHMUK ANEA 2019.7152, coll. 20 Feb. 2015, 12°32.23N, 116°36.25W, 4425 m http://data.nhm.ac.uk/object/d84c37ed-138e-4064-a11d-a11a2470dfdf; NHM_823 **(holotype)** NHMUK ANEA 2019.7153, coll. 20 Feb. 2015, 12°32.23N, 116°36.25W, 4425 m http://data.nhm.ac.uk/object/74781dbb-1f65-4839-a766-24d6cde63ed0; NHM_1423 (**paratype**) NHMUK ANEA 2019.7154, coll. 03 Mar. 2015, 12°27.26N, 116°36.77W, 4137 m http://data.nhm.ac.uk/object/d949e987-6e03-4092-8492-c51dd7fcf4d7; NHM_1895 NHMUK ANEA 2019.7155, coll. 13 Mar. 2015, 12°02.49N, 117°13.03W, 4094 m, http://data.nhm.ac.uk/object/02aaa9c0-837a-4836-8b34-5e68296c958e.

##### Type locality.

Pacific Ocean, CCZ, 12°32.23N, 116°36.25W, depth 4425 m, in mud between polymetallic nodules.

##### Description.

**Figure 24.**
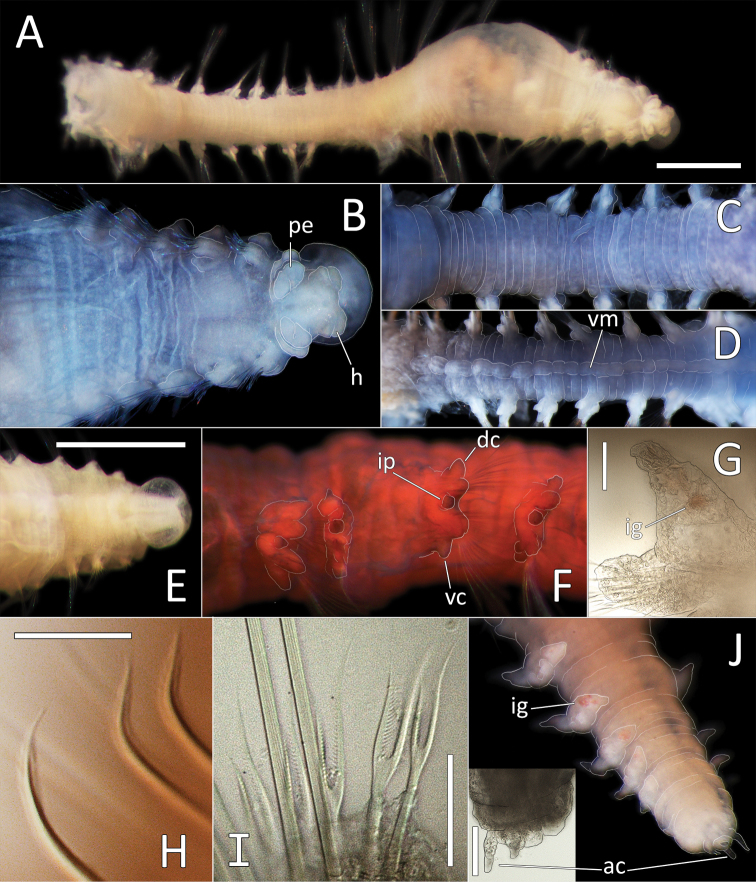
*Oligobregma brasierae* sp. nov. **A** Lab image, whole specimen (holotype [specimen NHM_823], pre-staining) **B** Lab image, dorsal anterior (holotype, faded stain, h = prostomial horns, pe = peristomial ring) **C** Lab image, dorsal segments, quadriannulate chaetigers (holotype, faded stain) **D** Lab image, ventral segments (holotype, faded stain, vm = ventral midline) **E** Lab image, ventral anterior, (holotype, pre-staining) **F** Lab image, mid-body parapodia (holotype, shirlastained, dc = dorsal cirrus, vc = ventral cirrus, ip = interramal papilla) **G** Lab image, detail of dorsal cirrus (paratype NHM_684, ig = internal gland) **H** Lab image, detail of hirstute notopodial spines on chaetiger 1 (paratype NHM_684, ig = internal gland) **I** Lab image, detail of capillary and lyrate chaetae (paratype NHM_684) **J** Lab images, posterior (paratype NHM_684, [bottom-left panel], ac = anal cirri, ig = internal gland). Morphological features in plates **B–D, F, G, H** have been outlined with a fine white or black line to improve clarity of those features. Scale bars: 1 mm (**A, E**); 50 μm (**G**); 25 μm (**H, I**); 100 μm (**J**).

**Figure 25.**
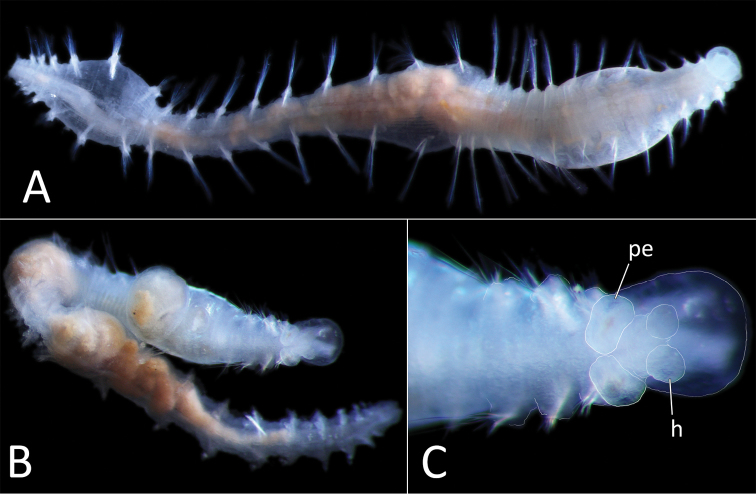
*Oligobregma brasierae* sp. nov. **A** Live image, whole specimen, ventral view (holotype [specimen NHM_823]) **B** Live image, whole specimen, dorsal view (paratype NHM_684) **C** Live image, dorsal anterior, with prostomial features outlined in a fine white line(paratype NHM_684, h = prostomial horns, pe = peristomial ring).

Small species, represented by six specimens. Holotype posteriorly incomplete, but otherwise in good condition, 9 mm long and 1 mm wide at the widest point for 24 chaetigers; paratypes complete, 6.0–6.5 mm long and 0.5–0.7 mm wide for 26 chaetigers. Body most expanded (inflated) through chaetigers 5–9, thereafter narrowing to posterior end. Colour in alcohol creamy white, without body pigment (Fig. 24A); live specimens translucent (Fig. 25)

Anterior body segments smooth, no obvious annulation of raised pads detected (even after staining) (Fig. 24B); annulation becomes most distinct in narrow, posterior part of the body, where segments quadriannulate (Fig. 24C). Venter with prominent ventral midline from chaetiger 2 composed of a row of large pads within a groove (Fig. 24D). Branchiae absent.

Prostomium broadly rounded anteriorly, weakly expanded laterally, narrowing posteriorly; with two short, rounded lobes (horns) emerging anterolaterally from anterior prostomial margin (Figs 24B, 25C). Eyes absent. Proboscis observed as a soft, smooth sac-like structure (Fig. 24E). Peristomium forming a smooth large ring around prostomium dorso-laterally, interrupted middorsally (Figs 24B, 25C), ventrally obscured by extended proboscis in holotype.

Parapodia biramous; inconspicuous in chaetigers 1–7, becoming longer posteriorly and prominent from around chaetiger 14. Tiny dorsal cirri detectable from chaetigers 14 in holotype, whereas ventral cirri occur from chaetiger 15 where well developed; both cirri large on subsequent segments; conical with broad base (Fig. 24F); without pigmentation; both dorsal and ventral cirri with detectable gold-pigmented internal glands (Fig. 24G). Interramal papilla present, inconspicuous in anterior parapodia (only observed upon staining), well developed in posterior parapodia Fig. 24F).

Curved acicular spines present in notopodia and neuropodia on chaetigers 1–4 (Fig. 24H). Notopodia with about 20 spines arranged in two rows in chaetigers 1 and 2, and with about 10 spines arranged in one row in chaetigers 3 and 4, spines accompanied posteriorly by single row of capillaries; neuropodial spines fewer in numbers arranged irregularly. Spines slightly curved, narrowing to slender elongated tip (Fig. 24H). Short spinous chaetae anterior to spines not observed. Subsequent chaetigers with long thin capillaries in both rami. Lyrate chaetae from chaetiger 5, in both rami, positioned anteriorly to capillaries. Lyrate chaetae short, with unequal tynes bearing short bristles (Fig. 24I), numbering two or three per noto- and neuropodium in anterior segments and up to six in posterior segments.

Single achaetigerous ring subsequent to the last chaetiger. Pygidium missing in holotype, but observed in paratypes; broad, triannulated, distally broadly rounded lobe; with few terminal, short anal cirri still attached in paratype NHM_684 (Fig. 24J).

Morphological variation: Some variability was noticed between different sized specimens. In the slightly bigger holotype (NHM_823) the spines can be observed on chaetigers 1–4 in both rami, and the dorsal cirri can be detected from chaetiger 14. In the smaller paratype (NHM_684), the spines cannot be unambiguously confirmed in ch. 4, particularly in neuropodia and dorsal cirrus can be detected from chaetiger 13.

##### Genetic data.

GenBank MN217422-MN217427 for 16S, MN217498 for 18S and MN217517 for COI. This species is genetically identical or very similar to sequences published in Janssen et al. (2015), with K2P values ranging from 0.0–0.003 between *O. brasierae* and the already published sequences with accession numbers KJ736359–KJ736363. The three *Oligobregma* species in this study form a well-supported clade in our phylogenetic analyses, with *Oligobregma brasierae* sp. nov. as sister to *Oligobregma tani* sp. nov. (Fig. 32).

##### Remarks.

Currently, there are nine valid species assigned to the genus *Oligobregma* (Read and Fauchald 2018b), with *O. blakei* Schüller & Hilbig, 2007 considered a nomen dubium. All three *Oligobregma* species from the ABYSSLINE material can be easily distinguished from those that have acicular spines in two (*O. pseudocollare* Schüller & Hilbig, 2007, *O. oculata* Kudenov & Blake, 1978) or three (*O. mucronata* Blake, 2015, *O. aciculata* (Hartman, 1965), *O. collare* (Levenstein, 1975), *O. notiale* Blake, 1981) anterior chaetigers only.

More specifically, *Oligobregma simplex* Kudenov & Blake, 1978, *O. lonchochaeta* Detinova, 1985 and *O. quadrispinosa* Schüller & Hilbig, 2007 share the presence of spines in chaetigers 1–4 with *O. brasieri* sp. nov., as well as having relatively large posterior dorsal and ventral cirri. *Oligobregma simplex* is a shallow water species (Western Port, Victoria, Australia, 11 m) and, while similar in size (5 mm long), it has a greater number of chaetigers (43 versus 26 in UKSR species) and more posterior appearance of dorsal and ventral cirri (on ch. 20–22 versus ch. 13–15). *Oligobregma lonchochaeta* has been described from a single, incomplete specimen from the abyssal North Atlantic, but its description is brief, not including the observation on the appearance of dorsal and ventral cirri, and there are no DNA data. Detinova (1985) differentiated her species from *O. simplex* by having first four chaetigers triannulate rather than uniannulate. However, there appears to be a typographical mistake in description of *O. simplex* by Kudenov and Blake (1978), as the authors state: “Body segments are annulated as follows: chaetigers 1–12 are uniannulate; 3–4 biannulate; 5–12 (? or 15) quadriannulate.” It is likely that chaetigers 1 and 2 not 1 to 12 are uniannulate. *Oligobregma quadrispinosa* has been described from the lower bathyal and abyssal Southern Ocean (Scotia and Weddell Seas, in 2258–4069 m) and is most similar to UKSR species in possessing similar number of chaetigers (*n* = 28) and podial cirri can be also detected from around chaetiger 13 and 14 [(estimated from the drawing provided in the original description by Schüller and Hilbig (2007)]. However, the new species possess spines in both rami of chaetigers 1–4, while *O. quadrispinosa* has spines in notopodia only according to Schüller and Hilbig (2007).

##### Ecology.

Found in polymetallic nodule province of the eastern CCZ.

##### Etymology.

Named in honor of Madeleine Brasier, member of the science party of the ABYSSLINE AB02 cruise onboard the RV *Thomas G. Thompson*.

#### Oligobregma tani sp. nov.

Taxon classificationAnimaliaCapitellidaCapitellidae

30598BA5-EF78-5686-9C12-AF76FA98A389

http://zoobank.org/EB95F031-2A2D-449D-8F2A-114EC628C9D2

Fig. 26A–J

##### Material examined.

NHM_773A (**paratype**) NHMUK ANEA 2019.7156, coll. 20 Feb. 2015, 12°32.23N, 116°36.25W, 4425 m http://data.nhm.ac.uk/object/4b673a6a-9090-4c24-a4eb-231190507b60; NHM_1454 **(holotype)** NHMUK ANEA 2019.7157, coll. 03 Mar. 2015, 12°27.26N, 116°36.77W, 4137 m http://data.nhm.ac.uk/object/67d3f58a-9c13-423e-93b7-3ddcf98a361e; NHM_1480J NHMUK ANEA 2019.7158, coll. 03 Mar. 2015, 12°27.26N, 116°36.77W, 4137 m http://data.nhm.ac.uk/object/d47f17aa-c0c1-44f0-a448-d3f3c395fc47; NHM_1665 (**paratype**) NHMUK ANEA 2019.7159, coll. 10 Mar. 2015, 12°21.81N, 116°40.86W, 4233 m http://data.nhm.ac.uk/object/eca166ae-3fe0-4367-860f-08c7410165dd.

##### Type locality.

Pacific Ocean, CCZ, 12°27.26N, 116°36.77W, depth 4137 m, in mud between polymetallic nodules.

##### Description.

**Figure 26.**
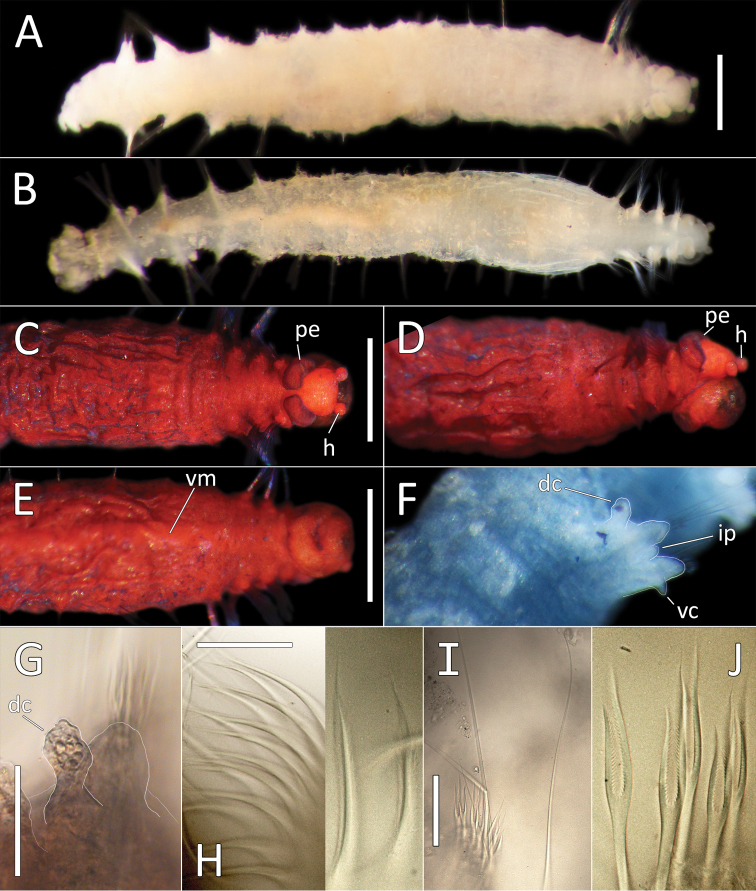
*Oligobregma tani* sp. nov. holotype (specimen NHM_1454). **A** Lab image, whole specimen (pre-stain,) **B** Live image, whole specimen **C** Lab image, dorsal anterior, (stained, h = prostomial horns, pe = peristomial ring) **D** Lab image, lateral anterior, (stained, h = prostomial horns, pe = peristomial ring) **E** Lab image, ventral anterior, (stained, vm = ventral midline) **F** Lab image, mid-body parapodia (faded stain, dc = dorsal cirrus, vc = ventral cirrus, ip = interramal papilla) **G** Lab image, detail of dorsal cirrus, with no internal gland visible (dc = dorsal cirrus) **H** Lab image, detail of hirsute spines on notopodia on chaetiger 1 ([left panel]) **I** Lab image, detail of capillary and lyrate chaetae on chaetiger 12 **J** Lab image, detail lyrate chaetae. Morphological features in plates **F, G** have been outlined with a fine white line to improve clarity of those features. Scale bars: 0.5 mm (**A, C, E**); 50 μm (**G–I**).

Small species, represented by four posteriorly incomplete specimens, 4–4.5 mm long and 0.4–0.7 mm wide. Holotype posteriorly incomplete, but otherwise in good condition, 4.5 mm long and 0.7 mm wide at the widest point for 18 chaetigers long fragment. Colour in alcohol creamy white, without body pigment (Fig. 26A); live specimens semi-translucent (Fig. 26B). Anterior body segments appears smooth, annulation of raised pads detected best upon staining (Fig. 26C–E) revealing chaetigers 1–4 with two transverse rows of relatively large lobes; subsequent chaetigers may be tri-annulate, but epithelium with mostly with wrinkled appearance till end of fragment (chaetiger 18) (Fig. 26B–E).Ventral midline on venter not too prominent, from chaetiger 2, composed of a row of large pads within a groove (Fig. 26E). Branchiae absent.

Prostomium broad (wider than long), nearly oval; with two very prominent, distinctly rounded lobes (horns) emerging from anterior prostomial margin (Fig. 26C, D). Eyes absent. Peristomium forming a large smooth ring around prostomium dorso-laterally, with narrow interruption middorsally (Fig. 26C, D). Mouth obscured by everted proboscis in holotype, which is observed as soft inflated sac (Fig. 26E).

Parapodia biramous; inconspicuous in chaetigers 1–14, becoming conical and prominent from around chaetiger 15. Tiny dorsal cirri detectable from chaetiger 13 in holotype, whereas ventral cirri occur from chaetiger 15; both cirri best developed from chaetigers 16 and 17, remaining small and conical (less than 1/2 the size of corresponding podial lobes) (Fig. 26F); without pigmentation; internal glands not detected in few cirri present (Fig. 26G). Interramal papilla present, well developed from chaetiger 15 (Fig. 26).

Curved acicular spines present in notopodia only on chaetigers 1‒4 (Fig. 26H); spines in chaetiger 4 transitional between distinct spines and capillaries. Notopodia with about 14 spines arranged in two rows in chaetigers 1and 2 and with about five spines arranged in one row in chaetigers 3 and 4; spines accompanied posteriorly by single row of capillaries. Spines in chaetigers 1–3 curved, straw-coloured, with hirsute shaft, narrowing to slender, elongated and hirsute tip (Fig. 26H); spines in chaetiger 4 transitional, more slender and straighter than in chaetigers 1–3, but with hirsute shafts and shorter unlike accompanying capillaries. Short spinous chaetae anterior to spines not observed. Subsequent chaetigers with long thin capillaries in both rami, but very few present (Fig. 26I). Lyrate chaetae likely from chaetiger 5 in both rami, where very short and difficult to observe; best observed from chaetiger 8; accompanied by very few capillaries. Lyrate chaetae initially short but becoming longer and very prominent from around chaetiger 8; with unequal tynes bearing short bristles (Fig. 26I, J), numbering up to 5 or 6 in each ramus. The rest of the body and pygidium unknown.

##### Genetic data.

GenBank MN217428–MN217431 for 16S, MN217499 for 18S and MN217518–MN217519 for COI. This species is genetically very similar to one sequence published in Janssen et al. (2015), with a K2P value of 0.008 between *O. tani* and the already published sequence with accession number KJ736365. The three *Oligobregma* species in this study form a well-supported clade, with *Oligobregma tani* sp. nov. as sister to *Oligobregma brasierae* sp. nov. in our phylogenetic analyses (Fig. 32).

##### Remarks.

The UKSR-collected species is most similar to *Oligobregma quadrispinosa* described from abyssal Southern Ocean (Schüller and Hilbig 2007) in having the first four notopodia with acicular spines, lyrate chaetae from chaetiger 5 and podial cirri arising from around chaetiger 13–15. However, *O. quadrispinosa* differs in the following characters; spines in chaetiger 4 are prominent, stout and not hirstue, while the median and posterior chaetigers bear much larger ventral cirri. The UKSR species also has very prominent round “Mickey Mouse”-like anterior prostomial lobes (observed in all four specimens examined). For comparison with other *Oligobregma* species see Table 3.

**Table 3.** Comparison of *Oligobregma* species with spines in chaetigers 1–4, including the new UKSR-collected species. Information collected from the literature.

	Distribution of spines in chaetigers 1–4	Annulation of chaetigers	Appearance of podial cirri	No. of chaetigers	Presence of furcate chaetae
*O. simplex*	In both rami	1–2 uni-; 3–4 bi-; 5–12 or 15 quadriannulate	Chaetigers 20–22	43 (complete)	From chaetiger 6
*O. lonchochaeta*	In both rami	1–4 triannulate	In posterior chaetigers, detail not given	22+ (incomplete)	In mid and posterior chaetigers
*O. quadrispinosa*	In notopodia only	Anterior quadriannulate; posterior with 5 annuli	Chaetigers 13–14	28 (complete)	From chaetiger 5
*O. brasierae* sp. nov.	In both rami, spines hirsute	Anterior smooth, posterior quadriannulate	Chaetigers 13–15	26 (complete)	From chaetiger 5
*O. tani* sp. nov.	In notopodia only, spines hirsute, transitional in ch. 4	Not observed	Chaetigers 13–15	18 (incomplete)	From chaetiger 5
*O. whaleyi* sp. nov.	In both rami	Anterior smooth, midbody quadriannulate	Chaetiger 14	26 (incomplete)	First observed from chaetiger 11

##### Ecology.

Found in polymetallic nodule province of the eastern CCZ.

##### Etymology.

Named in honor of Koh Siang Tan, member of the science party of the ABYSSLINE AB02 cruise onboard the RV *Thomas G. Thompson*.

#### Oligobregma whaleyi sp. nov.

Taxon classificationAnimaliaCapitellidaCapitellidae

5706DC61-D68E-5111-B6A7-F7AAE946678E

http://zoobank.org/6856E564-D7EC-42B0-8DED-9CCB95B8ABFE

Fig. 27A–I

##### Material examined.

NHM_822 (**holotype**) NHMUK ANEA 2019.7160, coll. 20 Feb. 2015, 12°32.23N, 116°36.25W, 4425 m http://data.nhm.ac.uk/object/dde1c8f9-f87a-430b-be9d-5e34685772bb.

##### Type locality.

Pacific Ocean, CCZ, 12°32.23N, 116°36.25W, depth 4425 m, in mud between polymetallic nodules.

##### Description.

**Figure 27.**
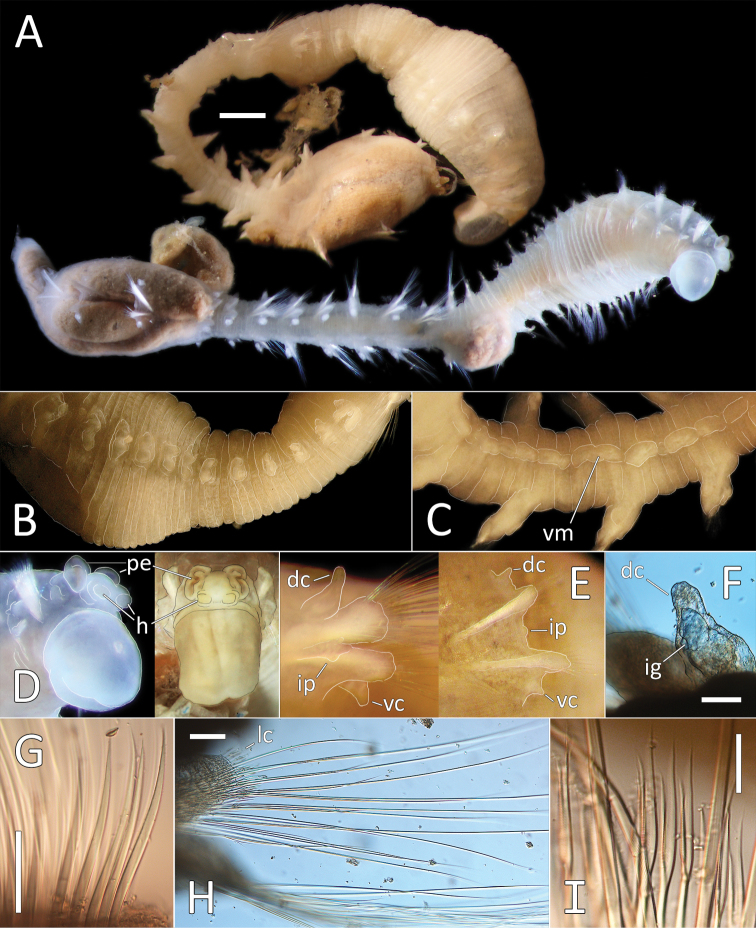
*Oligobregma whaleyi* sp. nov. holotype (specimen NHM_822). **A** Lab (upper) and live (lower) images, whole specimens [lab image] **B** Lab image, mid-body segments and annulation **C** Lab image, ventral midbody (vm = ventral midline) **D** Live (left) and lab (right) images of prostomium (pe = peristomial ring, h = prostomial horns) **E** Lab images, midbody and posterior parapodia, chaetigers 15 (left) and 24 (right) (dc = dorsal cirrus, vc = ventral cirrus, ip = interramal papilla) **F** Lab image, detail of dorsal cirrus and internal gland (dc = dorsal cirrus, ig = internal gland) **G** Lab image, detail of notopodial spines on chaetiger 1 **H** Lab image, detail of capillary chaetae (lc = lyrate chaetae) **I** Lab image, detail of lyrate chaetae. Morphological features in plates **B–D, F, G, H** have been outlined with a fine white or black line to improve clarity of those features. Scale bars: 1 mm (**A**); 100 μm (**F–H**); 50 μm (**I**).

Large species, represented by a single, posteriorly incomplete specimen, with 26 chaetigers, 16 mm long and about 2 mm wide at widest (inflated) region (first eight chaetigers, particularly chaetigers 3–8), with another widening of the body in chaetigers 23–26, likely due to sediment ingestion. Colour in alcohol creamy white, without body pigment, live specimens semi-translucent (Fig. 27A). Anterior body segments appear smooth, without distinct annulation, chaetigers 1–5 with three transverse rows of weakly developed lobes; subsequent segments quadriannulated until the end of the fragment (chaetiger 26) (Fig. 27B, C). Ventral midline on venter prominent, from chaetiger 2, composed of a row of large pads within a groove (Fig. 27C). Branchiae absent.

Prostomium broadly rounded anteriorly, weakly expanded laterally, narrowing posteriorly; with two well-developed, anterior rounded lobes (horns) emerging from anterior prostomial margin (Fig. 27D). Eyes absent. Proboscis observed as a soft, smooth sac-like structure. Peristomium forming smooth figure-of-8-like loops laterally to prostomium (Fig. 27D), dorsally interrupted, ventrally obscured by extended proboscis; with faint light-brown pigmentation.

Parapodia biramous; conspicuous even in anterior-most segments (Fig. 27E), becoming longer and prominent from around chaetiger 10. Dorsal and ventral cirri appear abruptly from chaetiger 14, where similar to those on subsequent segment; relatively small (about 1/2 the size of associated podial lobes) in posterior chaetiger; all conical with broad base (Fig. 27E), without pigmentation; some dorsal and ventral cirri with gold-pigmented internal glands, now bluish upon uptake of Shirlastain (Fig. 27F). Interramal papilla present, inconspicuous in anterior parapodia, well developed from chaetiger 10 (Fig. 27E).

Curved acicular spines present in notopodia and neuropodia on chaetigers 1‒4. Notopodia with about 15 spines arranged in irregular row, accompanied posteriorly by single row of capillaries; neuropodial spines fewer in numbers arranged irregularly. Spines slightly curved, narrowing to slender elongated tip (Fig. 27G). Short spinous chaetae anterior to spines not observed. Subsequent chaetigers with long thin capillaries in both rami (Fig. 27H). Lyrate chaetae at least from chaetiger 11, in both rami, positioned anteriorly to capillaries. Lyrate chaetae short, with unequal tynes bearing short bristles (Fig. 27I), numbering 12–20 per noto- and neuropodium. The rest of the body and pygidium unknown.

##### Genetic data.

GenBank MN217432 for 16S and MN217500 for 18S. The three *Oligobregma* species in this study form a well-supported clade. *Oligobregma whaleyi* sp. nov. is sister to a clade consisting of *Oligobregma tani* sp. nov. and *Oligobregma brasierae* sp. nov. in our phylogenetic analyses (Fig. 32).

##### Remarks.

The UKSR-collected species *O. whaleyi* sp. nov. differs from other *Oligobregma* species bearing spines on the first four chaetigers in having a peristomial ring forming a figure-of-8 loops laterally to prostomium and in furcate chaetae appearing more posteriorly (first observed on chaetiger 11 although due to its large size the specimen was difficult to manipulate and removal of several parapodia would have damaged the single specimen significantly), while in other species the furcate chaetae are present from chaetiger 6. In *O. lonchochaeta*Detinova (1985) described the furcate chaetae as occurring only in mid- and posterior chaetigers but without specifically stating on which chaetiger they were first observed. Therefore, the newly described species can be distinguished from *O. lonchochaeta* by having the anterior chaetigers smooth, rather than triannulated. For further comparison see Table 3.

##### Ecology.

Found in polymetallic nodule province of the eastern CCZ.

##### Etymology.

Named in honor of Jeremy Whaley, Able Seaman onboard RV *Melville* on the ABYSSLINE cruise AB01 in 2013.

#### Scalibregmatidae

Taxon classificationAnimaliaCapitellidaCapitellidae

sp. (NHM_2308)

C3E1567D-51A2-56BA-A577-ED6767B05E9E

Fig. 28A–F

##### Material examined.

NHM_2308 NHMUK ANEA 2019.7161, coll. 01 Mar. 2015, 12°15.44N, 117°18.13W, 4302 m http://data.nhm.ac.uk/object/7b9d4ab8-4b7b-45c4-9cf4-6fd6b1229f48.

##### Description.

**Figure 28.**
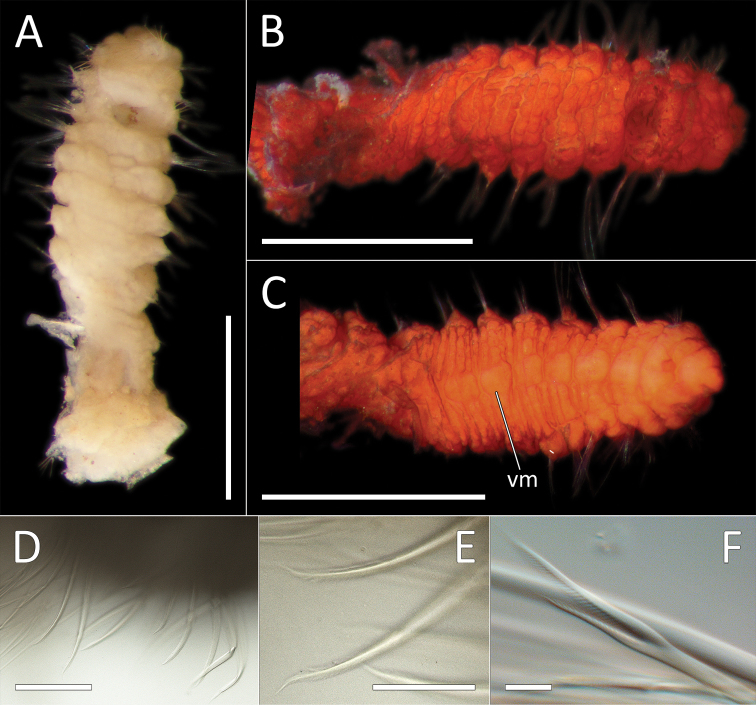
Scalibregmatidae sp. NHM_2308 (specimen NHM_2308). **A** Lab image, whole specimen, dorsal view (pre-stain) **B** Lab image, dorsal anterior (stained) **C** Lab image, ventral anterior, (shirla stained, vm = ventral midline) **D** Lab image, detail of hirsute spines on chaetigers 1 and 2 **E** Lab image, detail of hirsute spine tips **F** Lab image, detail of lyrate chaeta. Scale bars: 1 mm (**A–C**); 100 μm (**D**); 50 μm (**E**); 10 μm (**F**).

This species is represented by a single, small, posteriorly incomplete specimen, 2.5 mm long and 0.4 mm wide for about 12 chaetigers, in poor condition. Colour of preserved specimen creamy yellow (Fig. 28A). Anterior part of the body rugose, chaetigers 1–4 dorsally with two? (some damaged) transverse rows of tightly packed squarish lobes; subsequent chaetigers with three such row or damaged (Fig. 28B). Venter with prominent ventral midline from chaetiger 2 composed of a row of large pads within a groove (Fig. 28C).

Prostomium small, broadly rounded anteriorly, weakly expanded laterally, narrowing posteriorly; with two short, rounded lobes (horns) emerging anterolaterally from anterior prostomial margin (Fig. 28D). Eyes absent. Branchiae absent.

Heavily curved acicular spines present in notopodia only on chaetigers 1 and 2 (Fig. 28D). Notopodia of chaetigers 1, bearing 10 very prominent spines arranged in two rows, accompanied posteriorly by single row of capillaries; notopodia of chaetiger 2 with five spines arranged in a single row, accompanied posteriorly by single row of capillaries. Spines relatively straight, stout, straw-coloured, distally narrowing to slender, elongated and somewhat hairy tip (Fig. 28E). Short spinous chaetae anterior to heavy spines not observed. Neuropodia of chaetiger 1 and 2 with long slender capillaries; subsequent chaetigers with long thin capillaries in both rami. Lyrate chaetae from chaetiger 3 in both rami, positioned anteriorly to capillaries. Lyrate chaetae short, with unequal tynes bearing short bristles (Fig. 28F), around 4 or 5 per ramus.

Parapodia biramous, podial lobes not developed in 12 chaetigers long fragment. Dorsal and ventral cirri not observed in 12 chaetiger long fragment. Rest of the body unknown.

##### Genetic data.

GenBank MN217467 for 16S. Scalibregmatidae (NHM_2308) does not cluster convincingly with any other Scalibregmatidae species available on GenBank (Fig. 32).

##### Remarks.

Poor preservation of mid body and missing posterior part prevents reliable identification to genus level. Observations from the anterior part (the absence of branchiae and presence of acicular spines) suggest that this may be yet another representative of genus *Oligobregma* in the UKSR-collected material. It can be distinguished from other scalibregmatid species in this study by having spines in notopodia of chaetigers 1 and 2 only.

### Travisiidae Hartmann-Schröder, 1971

#### Travisia

Taxon classificationAnimaliaCapitellidaCapitellidae

Johnston, 1840

9CF64C2F-3D8D-5761-B29E-7FB9777AAF51

##### Notes.

These distinctive, grub-like polychaetes with rugose epidermis were first described by Johnston (1840) with the discovery of *Travisia forbesii* Johnston, 1840. Later, Kinberg (1866) established the genus *Dindymenes* and Chamberlin (1919) established the genus *Kesun*, which he differentiated from *Travisia* by the complete absence of branchiae. Following a cladistic analysis of morphological characters, Dauvin and Bellan (1994) synonymized *Kesun* and *Dindymenides* with *Travisia* and recognized at least 27 species. Important species-level characters include the presence of lobes, the position and relative size of the nephridiopores, and the total number of chaetigers, which appears to be stable in most, but not all, species (Dauvin and Bellan 1994).

The higher taxonomic position of *Travisia* has been in dispute for some time. While usually placed in Opheliidae, its relationship with Scalibregmatidae has also been long suggested (Ashworth 1902), mainly due to possession of rugose epidermis. Hartmann-Schröder (1971) created a subfamily, Travisiinae, in Opheliidae to accommodate *Travisia*. More recently, phylogenetic analyses were employed to answer this question. Persson and Pleijel (2005) used molecular data to recover *Travisia* nested within the Scalibregmatidae, and molecular analysis of Paul et al. (2010) rejected affinity with Opheliidae and found strong support sister-group relationship of *Travisia* and Scalibregmatidae. Law et al. (2014) again placed *Travisia* within Scalibregmatidae using molecular data. However, Blake and Maciolek (2016) proposed a new family, Travisiidae, to accommodate *Travisia*.

*Travisia* species have predominantly deep-water distribution (Blake and Maciolek 2016) and two species, one of them very abundant, were found in UKSR material.

#### Travisia zieglerae sp. nov.

Taxon classificationAnimaliaCapitellidaCapitellidae

9E363687-CBEF-5922-8712-4845F06AF354

http://zoobank.org/74877AF0-D607-4C62-BD07-6844D90A2806

Fig. 29A–G

##### Material examined.

NHM_140 (**paratype**) NHMUK ANEA 2019.7162, coll. 11 Oct. 2013, 13°45.50N, 116°41.91W, 4080 m http://data.nhm.ac.uk/object/ed10356b-32a0-4b45-9fe3-c56fbc696e87; NHM_188 NHMUK ANEA 2019.7170, coll. 14 Oct. 2013, 13°57.43N, 116°30.10W, 4130 m http://data.nhm.ac.uk/object/c8a0ef70-e7f7-4605-bf78-dc54ed9151eb; NHM_241 NHMUK ANEA 2019.7163, coll. 16 Oct. 2013, 13°48.70N, 116°42.60W, 4076 m http://data.nhm.ac.uk/object/5c0ac0b7-60cc-473e-a23b-2f49a40540f4; NHM_356 NHMUK ANEA 2019.7164, coll. 17 Oct. 2013, 13°45.21N, 116°29.12W, 4128 m http://data.nhm.ac.uk/object/8d2cbf0e-6522-403d-a58a-905fb13c70d6; NHM_364 NHMUK ANEA 2019.7165, coll. 19 Oct. 2013, 13°55.98N, 116°42.977W, 4182 m http://data.nhm.ac.uk/object/ef6e520f-7ef5-4ff9-87b5-985b8576271f; NHM_748B (**paratype**) NHMUK ANEA 2019.7166, coll. 20 Feb. 2015, 12°32.23N, 116°36.25W, 4425 m http://data.nhm.ac.uk/object/db527676-1030-4bf0-b28d-2382825bc6bf; NHM_753 NHMUK ANEA 2019.7167, coll. 20 Feb. 2015, 12°32.23N, 116°36.25W, 4425 m http://data.nhm.ac.uk/object/393203b1-cb80-4185-9e40-fca6e1b6fe34; NHM_760 NHMUK ANEA 2019.7168, coll. 20 Feb. 2015, 12°32.23N, 116°36.25W, 4425 m http://data.nhm.ac.uk/object/d3e8ec3c-d7f3-4908-b315-84f3758aecc1; NHM_792 NHMUK ANEA 2019.7169, coll. 20 Feb. 2015, 12°32.23N, 116°36.25W, 4425 m http://data.nhm.ac.uk/object/5d30a61b-5894-484f-b79a-df1cd4268ec1; NHM_909 (**paratype**) NHMUK ANEA 2019.7171, coll. 23 Feb. 2015, 12°34.28N, 116°36.63W, 4198 m http://data.nhm.ac.uk/object/5f570dab-4b56-4f74-b126-ed6ceab344e3; NHM_970 NHMUK ANEA 2019.7172, coll. 23 Feb. 2015, 12°34.28N, 116°36.63W, 4198 m http://data.nhm.ac.uk/object/4ccb364c-35f4-458c-9c71-6f77e71493ca; NHM_1097 NHMUK ANEA 2019.7173, coll. 26 Feb. 2015, 12°06.93N, 117°09.87W, 4100 m, http://data.nhm.ac.uk/object/939ba16d-b844-49ca-a740-bb42f039cc11; NHM_1310 NHMUK ANEA 2019.71745,coll. 01 Mar. 2015, 12°15.44N, 117°18.13W, 4302 m http://data.nhm.ac.uk/object/16844478-de27-448c-9acb-057835026447; NHM_1311 NHMUK ANEA 2019.7175, coll. 01 Mar. 2015, 12°15.44N, 117°18.13W, 4302 m http://data.nhm.ac.uk/object/192cbbb3-680b-4bcd-9cc4-a420f42af578; NHM_1431 (**holotype**) NHMUK ANEA 2019.7176, coll. 03 Mar. 2015, 12°27.26N, 116°36.77W, 4137 m http://data.nhm.ac.uk/object/fd6bab0e-0cda-4b42-808f-a6006d409535; NHM_1543 (**paratype**) NHMUK ANEA 2019.7177, coll. 06 Mar. 2015, 12°30.38N, 116°29.07W, 4244 m http://data.nhm.ac.uk/object/c78cc5fd-ca98-43b0-a0fb-8804fb606c71; NHM_1873 NHMUK ANEA 2019.7178, coll. 13 Mar. 2015, 12°02.496N, 117°13.03W, 4094m, http://data.nhm.ac.uk/object/24409a12-2a50-4689-80dc-902cdeb5af69; NHM_1883 NHMUK ANEA 2019.7179, coll. 13 Mar. 2015, 12°02.49N, 117°13.03W, 4094 m, http://data.nhm.ac.uk/object/9e8c22f7-a94b-45ed-a1d0-cae287a7ac2d; NHM_1911 NHMUK ANEA 2019.7180, coll. 13 Mar. 2015 12°02.49N, 117°13.03W, 4094 m http://data.nhm.ac.uk/object/489dd5a6-2c68-416b-9a06-ed773d4791d6; NHM_2019 NHMUK ANEA 2019.7181, coll. 16 Mar. 2015, 12°03.03N, 117°24.28W, 4235 m http://data.nhm.ac.uk/object/2684a5f8-b4d4-4bcb-b386-65775506cf87; NHM_2024 NHMUK ANEA 2019.7182, coll. 16 Mar. 2015, 12°03.03N, 117°24.28W, 4235 m http://data.nhm.ac.uk/object/cf54f81e-5836-4684-94dc-151f589ebab4.

##### Type locality.

Pacific Ocean, CCZ, 12°27.26’N 116°36.77’W, depth 4137 m, in mud between polymetallic nodules.

##### Additional material examined.

*Travisia glandulosa* McIntosh, 1879, holotype BMNH 1921.5.1.2431 and specimen of Monro (1930), *Travisia gravieri* McIntosh, 1908, holotype BMNH 1921.5.1.2429.

##### Description.

**Figure 29.**
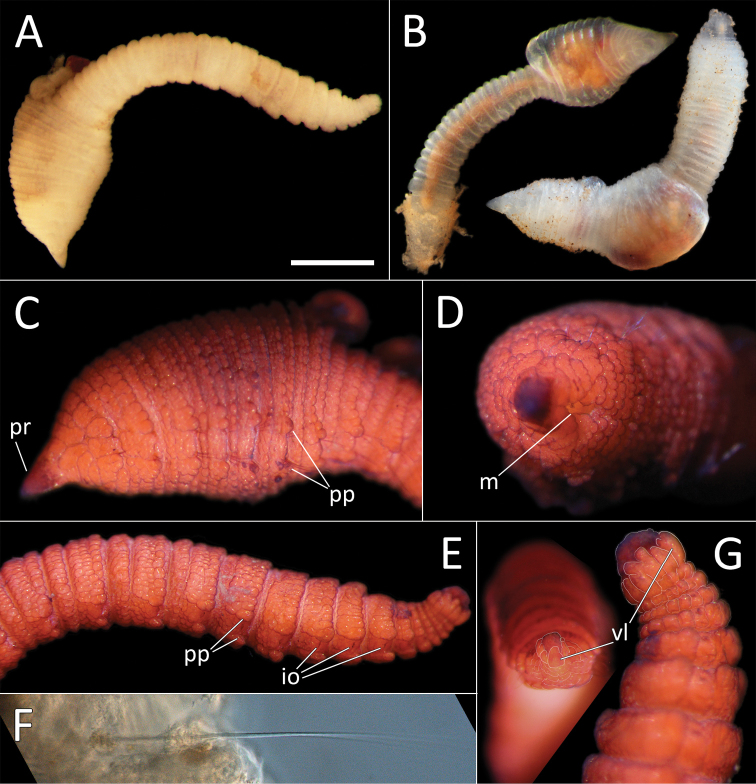
*Travisia zieglerae* sp. nov. **A** Lab image, whole specimen, pre-stain (holotype [specimen NHM_1431]) **B** Live images, whole specimens (specimen NHM_1911 [left], specimen NHM_188 [right]) **C** Lab image, lateral anterior, (holotype, stained, pr = prostomium, pp = parapodial lappets) **D** Lab image, distal anterior, (holotype, stained, m = mouth) **E** Lab image, lateral posterior, (holotype, stained, pp = parapodia, io = interramal organs) **F** Detail of capillary chaeta (paratype NHM_140) **G** Lab image, pygidium, distal view (left) and lateral view (right), with pygidial features outlined in a fine white line (holotype, stained, vl = ventral lobe). Scale bar: 1 mm (**A**).

This species is represented by 21 specimens. It is a small species 1.2–7.5 mm long and 0.25–0.8 mm wide for 21–24 segments, 19 or 20 of which chaetigerous and 2–4 posterior-most achaetigerous. Preserved specimens pale yellow (Fig. 29A), live specimens translucent (Fig. 29B)

Holotype in good condition, 6 mm long and 0.8 mm wide (at the widest point). Body robust, compact, grub like, anteriorly (commonly on chaetigers 1–7) somewhat enlarged then tapering posteriorly and relatively slender. Body surface rugose, with transverse rows of small squarish lobes.

Prostomium short, smooth, conical (Fig. 29C). Peristomium trapezoidal, rugose, with squarish papillae larger and then in subsequent segments, two transverse rows observed using Shirlastain A (Fig. 29C). Mouth as a broad transverse slit extending to chaetiger 1 (Fig. 29D).

Branchiae absent. Parapodia biramous, located on row with largest lobes, both rami well separated (Fig. 29C, E). Parapodial lappets present, observable from chaetiger 2 and well developed from chaetiger 8. Chaetigers in anterior (inflated) half distinctly triannulate, with three transverse rows of small, squarish lobes, subsequent segments becoming less distinctly annulated, with the last four achaetigerous segments uniannulate; lobes always largest on the ventral most row. Interramal sense organs present, best observed on stained specimen (Fig. 29E). Chaetae all long, smooth, slender capillaries (Fig. 29F).

Pygidium short, thick (only slightly longer wide), ventrally with keel-like very thick lobe. In distal view (Fig. 29G) with circlet of about 10 smaller, thinner lobes located dorsally to large ventral keel-like lobe.

##### Shirlastain pattern.

Prostomium stains strongly and stain is retained even after one week. Interramal sense organs observed as darkly red stained spots (Fig. 29E).

##### Morphological variation.

Number of segments is slightly variably and appears to be linked to size, with the smallest specimens possessing 21 segments (19 of which chaetigerous), while the largest specimen possessed 24 segments (20 of which chaetigerous). Body shape remains mainly consistent, although some specimens were slightly thinner or thicker. Thick, keel-like ventral lobe on pygidium observed consistently, but the detection of slenderer lobes differs (probably an artefact of preservation) and some occasionally appear inflated (Fig. 29G).

##### Remarks.

Differences between the known *Travisia* species and the species delineated herein are discussed in the Remarks section for *Travisia* sp. (NHM_1244), see below.

##### Genetic data.

GenBank MN217470–MN217490 for 16S and MN217512 for 18S. *Travisia zieglerae* sp. nov. fall within a clade consisting of the other *Travisia* species in this study as well as other *Travisia* species on GenBank and the taxon *Neolipobranchus* sp., a result similar to Martinez et al. (2014), suggesting a paraphyletic genus *Travisia* (Fig. 32).

##### Ecology.

Found in polymetallic nodule province of the eastern CCZ.

##### Etymology.

Named in honor of Amanda Ziegler, member of the science party of the ABYSSLINE AB02 cruise onboard the RV *Thomas G. Thompson*.

#### Travisia

Taxon classificationAnimaliaCapitellidaCapitellidae

sp. (NHM_1244)

FD955417-26C6-5985-843A-7A845E9095F4

Fig. 30A–G

##### Material examined.

NHM_1244 NHMUK ANEA 2019.7183, coll. 01 Mar. 2015, 12°15.44N, 117°18.13W, 4302 m http://data.nhm.ac.uk/object/f6906eae-67ec-4d37-83c6-590f3c53df76; NHM_1863 NHMUK ANEA 2019.7184, coll. 13 Mar. 2015, 12°02.49N, 117°13.03W, 4094 m, http://data.nhm.ac.uk/object/fa708aca-6dd1-4b53-8d54-c76a93f43363.

##### Description.

**Figure 30.**
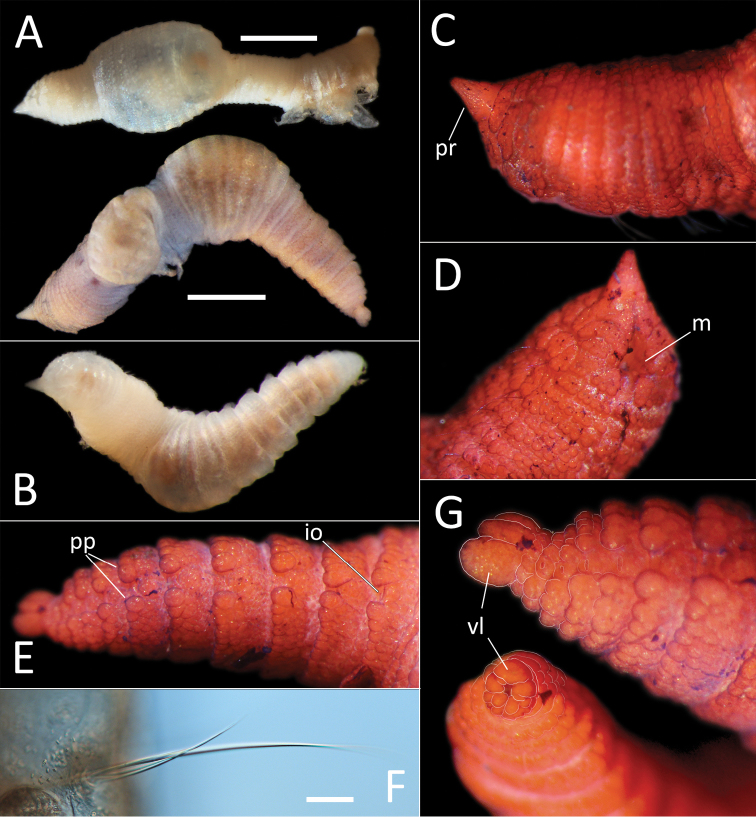
*Travisia* sp. NHM_1244. **A** Lab images, whole specimens (specimen NHM_1244, unstained [top], specimen NHM_1863, faded stain [bottom]) **B** Live image, whole specimen (specimen NHM_1863) **C** Lab image, dorsal anterior (specimen NHM_1863, stained, pr = prostomium) **D** Lab image, ventral anterior, (stained) (specimen NHM_1863, m = mouth) **E** Lab image, lateral posterior (specimen NHM_1863, stained, pp = parapodia, io = interramal organs) **F** Lab image, detail of capillary chaetae (specimen NHM_1244) **G** Lab image, pygidium, distal view (lower left) and lateral view (upper right), with pygidial features outlined in a fine white line (specimen NHM_1863, stained, vl = ventral lobe). Scale bars: 1 mm (**A**); 50 μm (**F**).

This species is represented by two specimens only. It is a small species 5.5–6 mm long and 0.7 mm wide for 24 segments, 20 of which chaetigerous and four posterior-most achaetigerous. Preserved specimens pale yellow (Fig. 30A), live specimens milky, semi-transparent (Fig. 30B). Body robust, compact, grub like, anteriorly half enlarged, particularly over chaetigers 11–13, tapering posteriorly, but remaining relatively thick. Body surface rugose, with transverse rows of small, tightly packed squarish lobes.

Prostomium short, smooth, conical (Fig. 30C, D). Peristomium trapezoidal, rugose, with small, tightly packed squarish papillae, appearing in two rows dorsally. Mouth as a transverse slit between chaetigers 1 and 2 (Fig. 30D).

Branchiae absent. Parapodia biramous, located on row with largest lobes, both rami well separated (Fig. 30E). Parapodial lappets present, observable from chaetiger 1 and well developed from chaetiger 6. Chaetigers in anterior half distinctly triannulate, with three transverse rows of small, squarish lobes, subsequent segments becoming less distinctly annulated, with the last four achaetigerous segments uniannulate; lobes always largest on the ventral most row. Interramal sense organs present, best observed on stained specimen (Fig. 30E). Chaetae all long, smooth, slender capillaries (Fig. 30F).

Pygidium very short, thick (slightly longer wide); in distal view with a tightly packed circlet of around 11 lobes (Fig. 30G), of these five large, thick and tightly packed with the ventral most single lobe thickest, dorso-laterally bordered by about six much smaller lobes (Fig. 30G).

##### Shirlastain pattern.

Stain retained uniformly. Interramal sense organs observed as darkly red stained spots (Fig. 30E). Specimen stain within few (about 5) days.

##### Genetic data.

GenBank MN217468–MN217469 for 16S and MN217511 for 18S. *Travisia* sp. (NHM_1244) is sister to *Neolipobranchus* sp. and fall within a clade consisting of *Travisia zieglerae* sp. nov. as well as other *Travisia* species from GenBank (Fig. 32).

##### Remarks.

**Figure 31.**
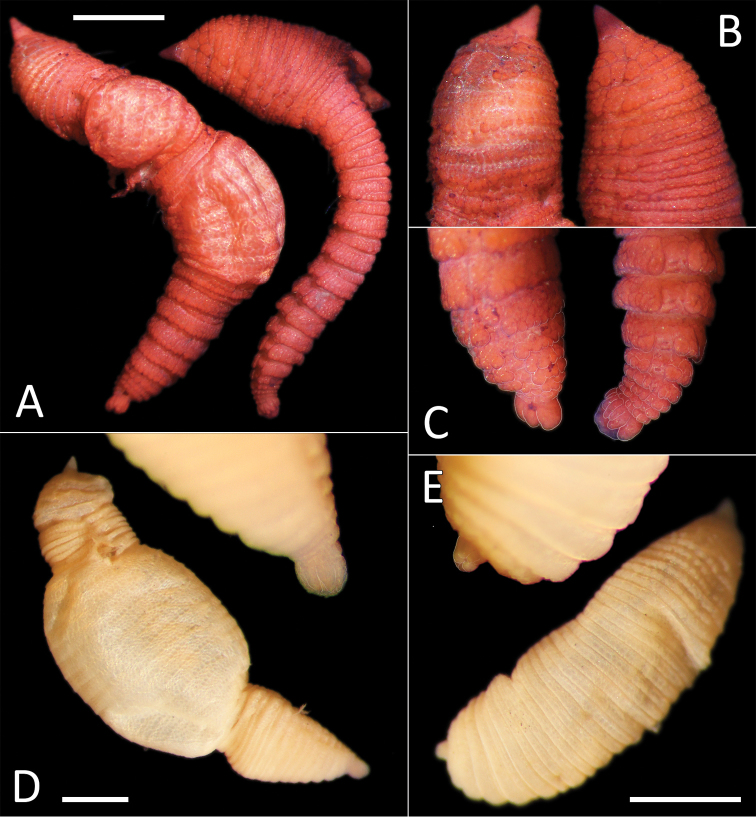
Comparison between *Travisia* sp. NHM_1244 and *Travisia zieglerae* sp. nov., and holotypes of *Travisia glandulosa* (BMNH 1921.5.1.2431) and *Travisia gravieri* (BMNH 1921.5.1.2429). **A** Lab images, whole specimens (*Travisia* sp. NHM_1244 specimen NHM_1863 [left], *Travisia zieglerae* sp. nov. holotype [specimen NHM_1431] [right], stained,) **B** Lab images, comparison of prostomia (*Travisia* sp. NHM_1244 specimen NHM_1863 [left], *Travisia zieglerae* sp. nov. holotype [right], stained) **C** Lab images, comparison of pygidia (*Travisia* sp. NHM_1244, specimen NHM_1863 [left], *Travisia zieglerae* sp. nov. holotype [right], stained) **D***Travisia glandulosa* holotype, with detail of pygidium **E***Travisia gravieri* holotype, with detail of pygidium. Morphological features in plates **C–E** have been outlined with a very fine white line to improve clarity of those features. Scale bars: 1 mm (**A, D, E**).

Both UKSR-collected species are morphologically very similar, in having a similar number of segments and in being abranchiate. They can be distinguished by a suite of subtle characters, which in case of *Travisia* sp. NHM_1224 is represented by only two specimens, so caution is needed. The two species differ somewhat in body shape as *Travisia zieglerae* sp. nov. is more slender in the posterior half, while Travisia sp. NHM_1224 is thicker (Fig. 31A). The rugosity of transverse rows differs, as the lobes are more tightly packed in *Travisia* sp. NHM_1244 and looser in *Travisia zieglerae* sp. nov., at least in the anterior region (Fig. 31B). Another difference is the arrangement of pygidial lobes (Fig. 31C). Finally, difference can be detected upon staining with Shirlastain, where prostomium of *Travisia zieglerae* sp. nov. stains darkly unlike that of *Travisia* sp. NHM_1244 (Fig. 31B) and the stain is retained after even 5 days since staining.

Of the known species of *Travisia*, only five were described as completely abranchiate, with four of these currently valid: *T. glandulosa* McIntosh, 1879; *T. gravieri* McIntosh, 1908; *T. nigrocincta* Ehlers, 1913 and *T. fusus* (Chamberlin, 1919) with *T. abyssorum* (Monro, 1930) considered a subjective synonym of *T. glandulosa*. Type specimens of McIntosh (1879; 1908) and the specimen of Monro (1930) were examined as part of this study. These can be distinguished from the UKSR species as follows:

*T. fusus*: has a larger body size of 14 mm and 28 chaetigers. Pygidium is divided into 12–14 inconspicuous lobes. Type locality: Pacific Ocean, towards the Marquesas Islands, 0°60’N, 137°54’W, 4504 m.

*T. glandulosa* (Fig. 31D): similar number of chaetiger to UKSR species (tentatively around 20 chaetigers counted), posteriorly thick, pygidium with circlet of about 12 small lobes; of these inner 4 or 5 also small. Original description of not much help, and McIntosh (1879) expressed doubt about naming the species due to its poor condition. Type locality: Arctic Ocean, Davis Strait, Greenland, 3264 m.

*T. gravieri* (Fig. 31E): very compact, grub-like, not thinner in posterior half; tentatively about 16 or 17 chaetigers observed. Type locality: North Atlantic, 986 m.

*T. nigrocincta*: much larger species, up to 34 mm long and 6 mm wide for 25 segments (the smallest specimen reported by Ehlers was 6 mm long and about 2 mm wide for 17 chaetigers), with dark transverse bands; pygidium not described in detail. Type locality: Southern Ocean, Wilhelm II Coast, 2725 m.

**Figure 32.**
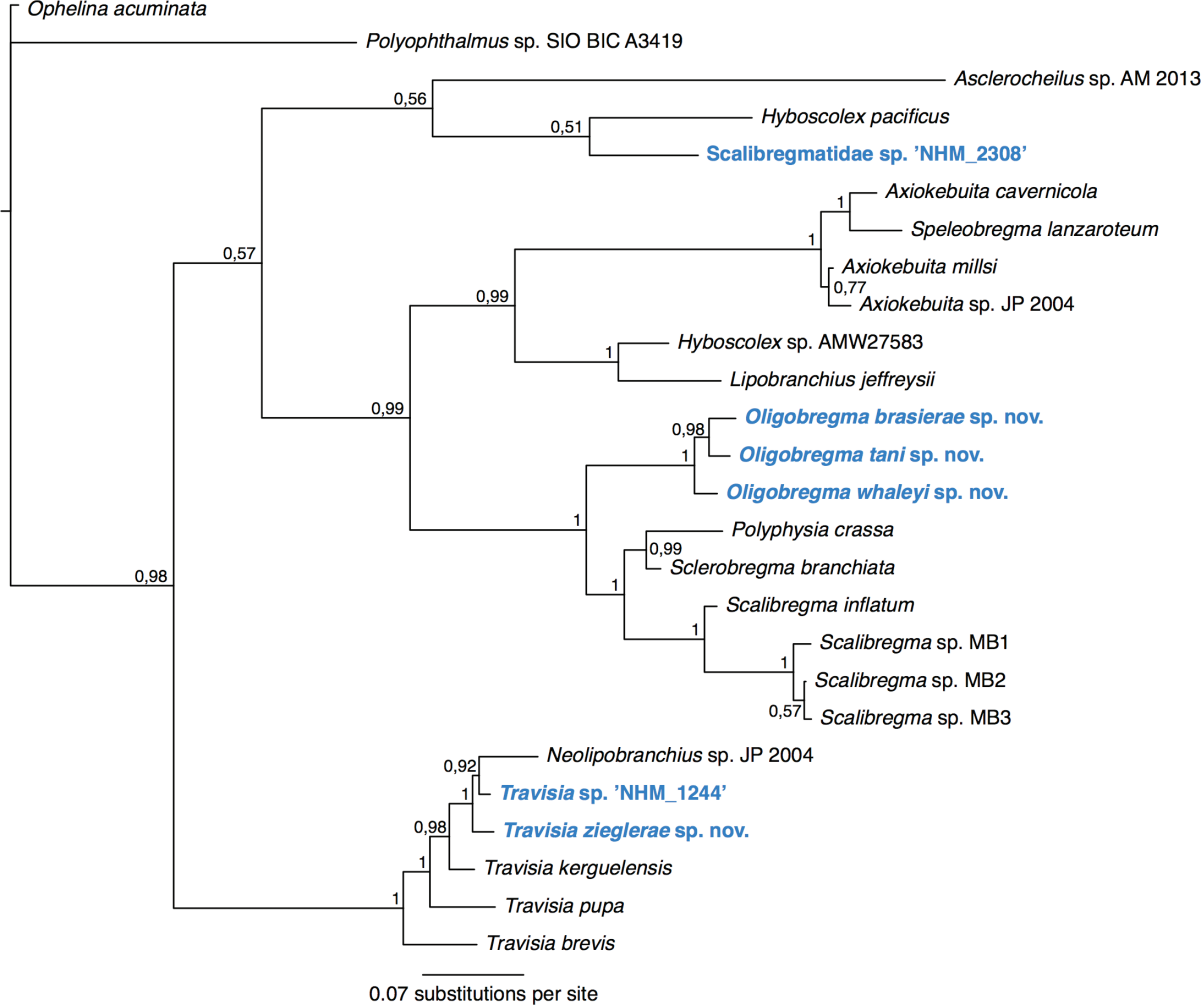
Phylogenetic analysis of Scalibregmatidae and Travisiidae. 50% majority rule tree from the Bayesian analyses using 18S and 16S, with posterior probability values on nodes. Twenty taxa from GenBank were included, and Opheliidae was chosen as outgroup following the annelid phylogeny of Weigert and Bleidorn (2016).

## Discussion

We have added 23 annelid species and 85 records to the total available knowledge of the benthic macrofauna of the CCZ. While this is certainly less than 10% of the estimated annelid diversity (based on the around 350 DNA-delineated species that are present in the UKSR collections), it represents a substantial increase in the published taxonomic knowledge linked to accessible voucher material, online genetic data and imagery of morphological features. Several of the taxa we report on are likely to be common and may have wide distributions across at least the eastern CCZ.

In terms of comparison to other studies, there are few sequences from just a few benthic faunal groups from the CCZ available on GenBank, for example echinoderms (Glover et al. 2016), cnidarians (Dahlgren et al. 2016), molluscs (Wiklund et al. 2017), polychaetes (Bonifácio and Menot 2018, Janssen et al. 2015), Porifera (Lim et al. 2017) and crustaceans (Janssen et al. 2015). With our study including both morphological and molecular data, we add to the knowledge of genetic information in the CCZ and aim to improve the taxonomic understanding of benthic fauna to provide a better picture of the distribution of taxa in the area, essential data for the establishment of conservation strategies in the light of potential future mineral extraction. These data are also the critical first step towards useful, practical identification guides to the fauna of this region.

## Supplementary Material

XML Treatment for Capitellidae

XML Treatment for Capitellidae

XML Treatment for Notomastus

XML Treatment for Opheliidae

XML Treatment for Ammotrypanella

XML Treatment for Ammotrypanella keenani

XML Treatment for Ammotrypanella kersteni

XML Treatment for Ammotrypanella

XML Treatment for Ammotrypanella

XML Treatment for Ophelina

XML Treatment for Ophelina curli

XML Treatment for Ophelina ganae

XML Treatment for Ophelina juhazi

XML Treatment for Ophelina martinezarbizui

XML Treatment for Ophelina meyerae

XML Treatment for Ophelina nunnallyi

XML Treatment for Ophelina cf. abranchiata

XML Treatment for Ophelina cf. abranchiata

XML Treatment for Ophelina cf. abranchiata

XML Treatment for Ophelina

XML Treatment for Ophelina

XML Treatment for Ophelina

XML Treatment for Scalibregmatidae

XML Treatment for Oligobregma brasierae

XML Treatment for Oligobregma tani

XML Treatment for Oligobregma whaleyi

XML Treatment for Scalibregmatidae

XML Treatment for Travisia

XML Treatment for Travisia zieglerae

XML Treatment for Travisia
